# Chemotranscriptomic profiling with a thiamine monophosphate photoaffinity probe†

**DOI:** 10.1039/d4sc06189f

**Published:** 2025-02-11

**Authors:** Stefan Crielaard, Casper F. M. Peters, Alexandar Slivkov, Daphne A. L. van den Homberg, Willem A. Velema

**Affiliations:** a Institute of Molecules and Materials, Radboud University Heyendaalseweg 135 Nijmegen 6525 AJ The Netherlands Willem.Velema@ru.nl

## Abstract

RNA is a multifaceted biomolecule with numerous biological functions and can interact with small molecule metabolites as exemplified by riboswitches. Here, we profile the *Escherichia coli* transcriptome on interactions with the metabolite Thiamine Monophosphate (TMP). We designed and synthesized a photoaffinity probe based on the scaffold of TMP and applied it to chemotranscriptomic profiling. Using next-generation RNA sequencing, several potential interactions between bacterial transcripts and the probe were identified. A remarkable interaction between the TMP probe and the well-characterized Flavin Mononucleotide (FMN) riboswitch was validated by RT-qPCR, and further verified with competition assays. Localization of the photocrosslinked nucleotides using reverse transcription and docking predictions of the probe suggested binding to the riboswitch aptamer. After examining binding of unmodified TMP to the riboswitch using SHAPE, we found selective yet moderate binding interactions, potentially mediated by the phosphate group of TMP. Lastly, TMP appeared to enhance gene expression of a reporter gene that is under riboswitch control, while the natural ligand FMN displayed an inhibitory effect, hinting at a potential biological role of TMP. This work showcases the possibility of chemotranscriptomic profiling to identify new RNA-small molecule interactions.

## Abstract 2

Photoaffinity labeling on *Escherichia coli* RNA extracts reveals an interaction between Thiamine Monophosphate (TMP) and the Flavin Mononucleotide (FMN) riboswitch.

## Graphical abstract

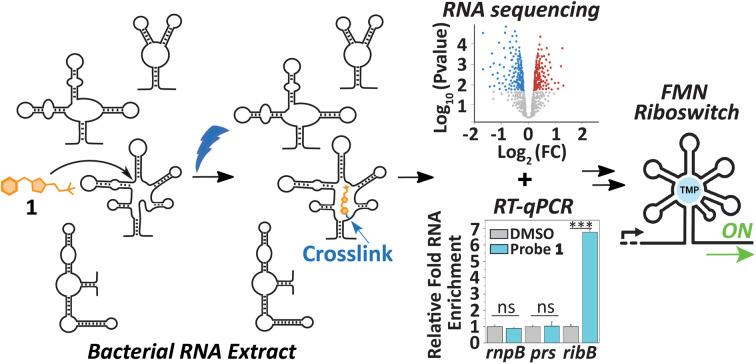

## Introduction

RNA is a versatile biomolecule that is crucial for living organisms.^1–3^ Besides its function as information carrier, RNA regulates a variety of critical cellular processes under both physiological and pathological conditions.^4–10^ Many RNAs can form intricate tertiary structures that selectively bind small molecule ligands and this interaction is essential for the RNA's biological function as exemplified by riboswitches and ribozymes.^11–19^

Riboswitches are fascinating biosensors that are used by many bacteria to control gene expression.^11–13,20–23^ These non-coding RNA elements generally interact with cellular small molecule metabolites that often contain flat heteroaromatic structures and charged groups, such as preQ_1_, Flavin Mononucleotide (FMN), *S*-Adenosyl Methionine, Tetrahydrofolate and Thiamine Pyrophosphate (TPP) among others.^15,24–32^

**Fig. 1:**
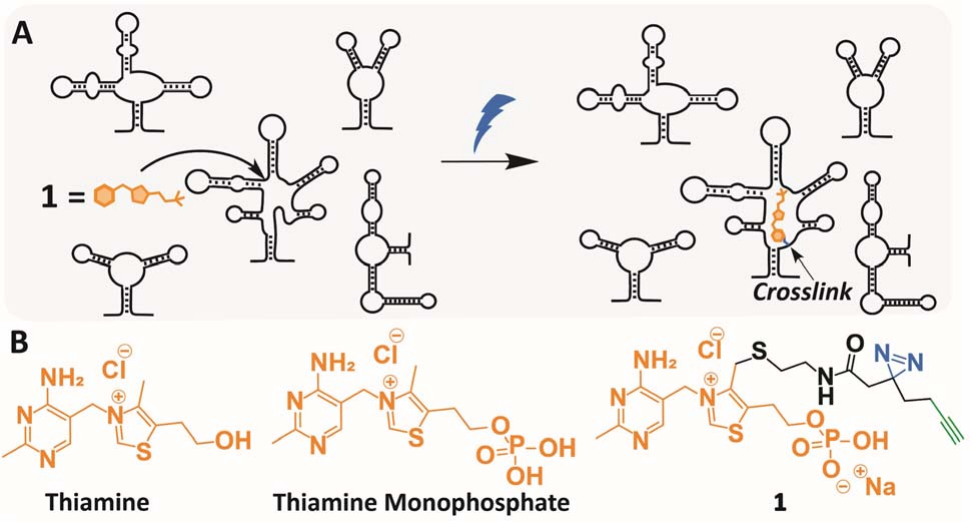
Transcriptome-wide crosslinking using a TMP photoaffinity probe. (A) Schematic illustration of chemotranscriptomic profiling using photocrosslinking. (B) Molecular structures of thiamine, thiamine monophosphate (TMP) and TMP probe 1.

To study interactions of small molecule ligands and RNA, several recent studies have demonstrated the great potential of (photo)chemical methods to capture these binding events, including pioneering work by the groups of Schultz and Johnson,^33^ Myers,^34^ Disney (CHEM-Clip),^35–37^ Petter (PEARL-seq),^38^ Schneekloth^39^ and Kool (RBRP).^40^ Since these approaches covalently capture interactions between small molecules and RNA, they allow for discovering RNA binding sites within a collection of RNAs (Fig. 1A), such as the transcriptome, when applied in conjunction with RNAseq.^33,34,38,39,41,42^

Inspired by these studies, we set out to identify potential binding pockets within the *Escherichia coli* (*E. coli*) transcriptome for Thiamine Monophosphate (TMP) (Fig. 1B). Notably, this small molecule metabolite is an intermediate in the TPP biosynthesis, but has no identified physiological function of its own.^43,44^ Since its molecular structure contains many features that are found in RNA binding ligands and its doubly phosphorylated counterpart TPP binds to a well-characterized riboswitch in *E. coli*, we postulated that TMP might interact with specific binding sites within the transcriptome as well.^25,45–47^

To test this, we designed and synthesized a photoaffinity probe based on the scaffold of TMP. We applied the probe to chemotranscriptomic profiling in *E. coli* using RNAseq, resulting in a set of enriched genes.^39,42^ Surprisingly, after analysis a transcript containing the *ribB* FMN riboswitch was obtained as a potential hit, which was further validated using RT-qPCR and competition experiments with the natural ligand. Localization of the photocrosslinking sites in the RNA were determined with nucleotide resolution using a reverse transcription termination assay, and implied selective binding. Subsequently, the interactions of unmodified TMP and the FMN riboswitch were examined using SHAPE analysis and an *in vitro* transcription/translation assay. Remarkably, TMP appeared to enhance gene expression of a luciferase reporter gene that is under riboswitch control. We believe these results demonstrate the potential power of chemotranscriptomic profiling and warrant further exploration into the biological role of TMP.

## Results and discussion

### Design and synthesis of a TMP photoaffinity probe

**Fig. 2:**
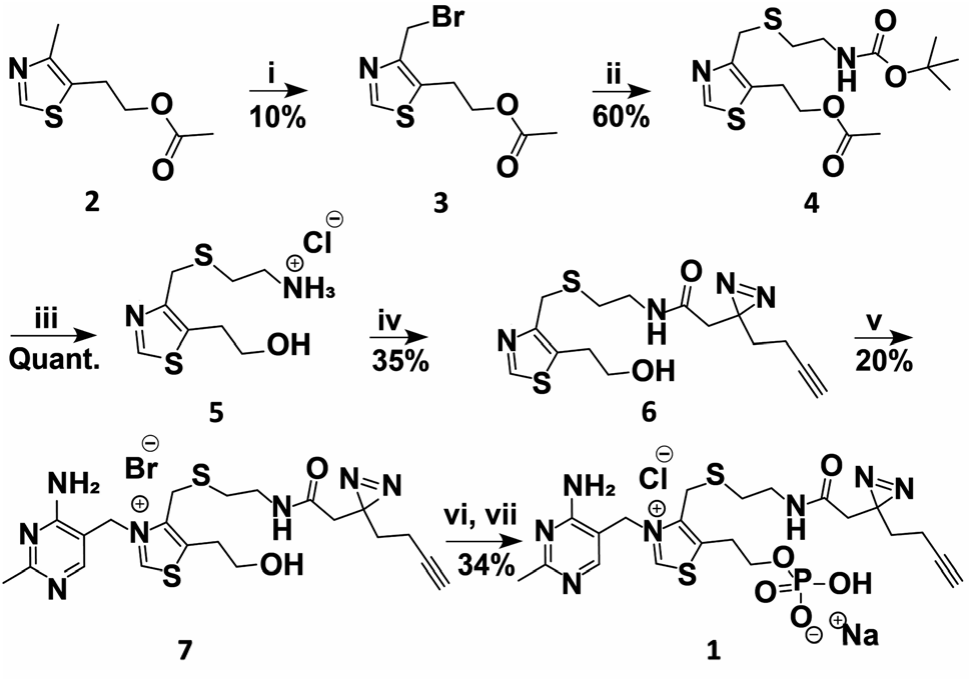
Synthesis of TMP photoaffinity probe 1. Reactions and conditions: (i) NBS, DCM, 370 nm light, rt, 15 min. (ii) 2-(Boc-amino)ethanethiol (9), K_2_CO_3_, ACN, rt, 3 h. (iii) 2 M HCl in MeOH/1,4-dioxane, rt, 2 h. (iv) 2-(3-(But-3-yn-1-yl)-3*H*-diazirin-3-yl)acetic acid (10), DIPEA, DMF, EDC·HCl, HOBt, rt, 16 h. (v) 4-Amino-5-bromomethyl-2-methylpyrimidine hydrobromide (11), DMF, 30 °C, 16 h. (vi) POCl_3_, TMP, 0 °C, 3 h (vii) NaClO_4_, acetone, 0 °C, 30 min. See ESI† for details.

A TMP photoaffinity probe was designed by attaching photoaffinity linker 10, containing a diazirine photocrosslinking moiety and an alkyne ligation handle, to the methyl group of the thiazolium ring at position 4 (Fig. 1B and 2). It was hypothesized that this methyl group is least likely to interact with potential RNA binding pockets, whereas the 2-methyl-4-aminopyrimidine, the anionic phosphate group and the cationic thiazolium ring all show potential to interact with a structured RNA.^16^ To this end, a bromide leaving group was introduced by bromination of 2 using *N*-bromosuccinimide and UV irradiation in dichloromethane. The bromide was substituted by 2-(boc-amino)ethanethiol 9 to obtain thioether 4. This compound was deprotected under acidic conditions and photoaffinity linker 10 was coupled using EDC, HOBt and DIPEA in DMF. Compound 6 was reacted with 11 to create thiamine analogue 7, which was phosphorylated, and salt exchanged to obtain TMP probe 1 (Fig. 2).

### Transcriptome-wide profiling using TMP probe 1

**Fig. 3:**
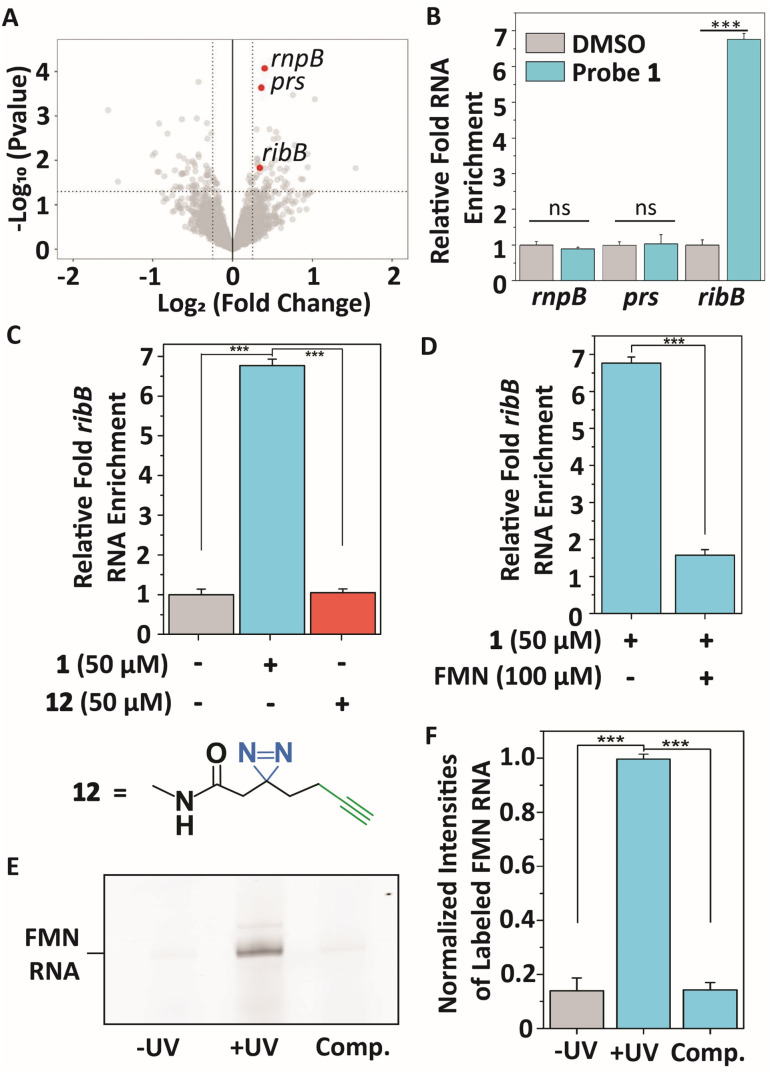
Transcriptome-wide profiling of interactions of TMP probe 1 with *E. coli* RNA extracts. (A) Volcano plot showing genes that were enriched after photocrosslinking with probe 1 compared to DMSO control samples. Significant enrichment limits were indicated by −Log10 (*P* value >1.3) and a Log2 (fold change) > 0.5. (B) Validating the interactions of the highlighted genes in (A) with probe 1 compared to DMSO control samples using RT-qPCR. Error bars indicate standard deviations of three biological replicates, each consisting of two technical replicates. (C) Relative enrichment of *ribB* RNA with probe 1 compared to DMSO control and control diazirine linker 12. (D) Relative enrichment of *ribB* RNA in the presence of FMN (100 μM). Error bars represent standard deviations of three biological triplicates, each consisting of two technical replicates. (E) Denaturing RNA gel showing selective photoaffinity labeling when TMP probe 1 was incubated with FMN riboswitch RNA. ‘−UV’ indicates no UV irradiation was performed during the experiment. ‘Comp’. indicates labeling in the presence of 100 μM FMN. RNA gels, corresponding RNA stained gels and an oligo ladder are included in Fig. S1.† (F) Quantification of gel band intensities observed in (E) and Fig. S1† in triplicate. ‘−UV’ indicates no UV irradiation was performed during the experiment. ‘Comp’. indicates labeling in the presence of 100 μM FMN. Error bars indicate standard deviations of triplicate measurements. Statistical significance was defined using an unpaired two-tailed Student's *t*-test (ns indicates non-significant (*p* > 0.05), ***p < 0.001).

To profile interactions between TMP probe 1 and a bacterial transcriptome, total RNA extract was isolated from *E. coli* (see ESI† for details), incubated with 50 μM 1 at 37 °C for 30 minutes to ensure all potential interactions can be captured, and exposed to 365 nm light to initiate covalent labeling. Next, a biotin group was attached to the alkyne handle of the probe-RNA complexes using copper(i)-catalyzed azide–alkyne cycloaddition (CuAAC). Biotinylated RNA-probe complexes were isolated using Streptavidin magnetic beads and purified for library preparation and next-generation sequencing (Fig. 3A). Reads were aligned to the *E. coli* K12 MG1655 genome using Bowtie2,^48^ summarized using FeatureCounts^49^ and RNA enrichments over DMSO control samples were analyzed using EdgeR.^50^ This analysis showed 55 potential significantly enriched genes that might interact with probe 1 (Table S1†). Next, we analyzed these hits on biological function (Table S1†) and size of untranslated region since most metabolite binding pockets reside in the 5′ UTR and are >50 nts.^2,12,51^ Based on these restrictions, we first focused on 3 bacterial transcripts: *rnpB*, *prs* and *ribB*. *rnpB* contains the essential catalytic RNA component of RNase P, *prs* encodes for a ribose-phosphate diphosphokinase and comprises a 5′ UTR of 301 nts, and *ribB* encodes a synthase involved in flavin biosynthesis and is controlled by a well-reported riboswitch. These 3 genes were further validated on interacting with probe 1 using RT-qPCR (Fig. 3A and B).

An approximate 6.7-fold enrichment of *ribB* was found when using probe 1 as compared to a DMSO control, whereas *rnpB* and *prs* showed no significant enrichment (Fig. 3B). Remarkably, TMP is not a known ligand for the FMN riboswitch. To further verify this result, control photoaffinity linker 12 was synthesized and tested (Fig. 3C), and showed no *ribB* enrichment, indicating that probe 1 selectively interacts with the FMN riboswitch-encoding transcript. To assess if TMP probe 1 interacts selectively with the aptamer of the FMN riboswitch, a competing amount of the FMN natural ligand (100 μM) was incubated with total *E. coli* RNA. A stark reduction in enrichment for *ribB* was observed in the presence of FMN (Fig. 3D), suggesting that 1 selectively binds to the FMN aptamer.

These results were further confirmed by incubating 25 μM probe 1 with 1 μM *in vitro* transcribed FMN riboswitch aptamer at 37 °C for 30 minutes. After UV irradiation, a fluorescein fluorophore was attached to the alkyne ligation handle using CuAAC and the probe-RNA complexes were analyzed by denaturing polyacrylamide gel electrophoresis (PAGE) (Fig. 3E, F and S1A, B†). This probe concentration was sufficient to observe labeling of the FMN riboswitch when both UV irradiation and fluorophore ligation steps were included. Selective photocrosslinking of the aptamer was demonstrated by pre-incubating with 100 μM FMN as competitor. The observed labeling signal disappeared, indicating that FMN and TMP probe 1 likely occupy the same binding pocket within the riboswitch (Fig. 3E, F and S1A, B†).

To further show selectivity of the interaction with the FMN riboswitch, the *in vitro* labeling experiment using 1 was repeated with the TPP riboswitch. This riboswitch has high affinity for TPP, which is structurally similar to TMP.^25,55^ A competition experiment with 100 μM TPP as competitor was also included. No selective binding to the TPP riboswitch aptamer was observed upon UV irradiation and fluorescent labeling (Fig. S2A and B†), thereby further confirming the selectivity of probe 1 for the FMN riboswitch compared to other RNAs (Fig. 3A and Table S1†).

### Localizing the TMP binding site

**Fig. 4:**
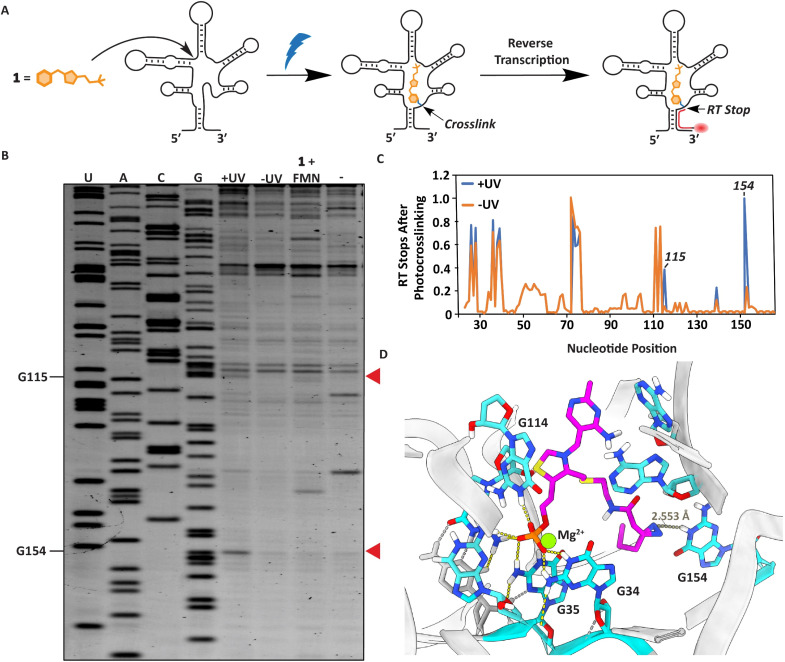
Localization of the photocrosslinks between probe 1 and the RNA aptamer. (A) Schematic illustration of the strategy to determine the photocrosslink sites in the RNA with nucleotide resolution. (B) PAGE gel after performing the RT stop assay. Red arrows indicate RT stops induced by UV irradiation compared to the shown controls. (C) Quantification of gel lane intensities observed in the PAGE gel. Gel lane intensities of all nucleotide bands were measured for the samples with (+) and without (−) UV irradiation. The quantification was performed using triplicate measurements of the RT stop assay (Fig. S3†). (D) Docking predictions of how TMP probe 1 can fit in the conserved FMN riboswitch aptamer of *Fusobacterium nucleatum* (PDB:3F2Q)^52,53^ with highlighted nucleotides annotated according to FMN riboswitch numbering of *ribD* from *Bacillus subtilis.*^54^

We next sought to localize the exact binding site of 1 within the FMN riboswitch using a reverse transcription termination assay (Fig. 4A).^38,56^ Defining the photocrosslinked nucleotides was performed by incubating 50 μM 1 with 1 μM RNA at 37 °C for 30 minutes, after which the interactions were captured by UV irradiation. This probe concentration was used to ensure a sufficient amount can covalently bind the FMN riboswitch upon light exposure and photocrosslinked nucleotides can be detected. The RNA was purified, and reverse transcription was performed using a fluorescently labeled primer, which stops where the probe was crosslinked to the RNA (Fig. 4A, see ESI† for details). The exact photocrosslinked nucleotides were deduced by sequence analysis using gel electrophoresis (Fig. 4B, C and S3†). New stops appeared at G115 and G154 after photocrosslinking. These stops were only observed after photocrosslinking 1 to the RNA. When 100 μM FMN was added in addition to probe 1 to compete for RNA binding, the RT stops disappeared, implying that these RT stops are indeed specific and originate from photocrosslinking of the probe to the RNA (Fig. 4B, C and S3†). The photocrosslinked nucleotides are reported to be in aptamer regions that are important for ligand binding. Additionally, molecular docking of probe 1 in the conserved riboswitch aptamer predicted a similar interaction pattern at the phosphate side as observed with FMN, whereas the diazirine is pointed in the direction of G154 where we observe a stop (Fig. 4D and S4A–C†).

### RNA SHAPE analysis

**Fig. 5:**
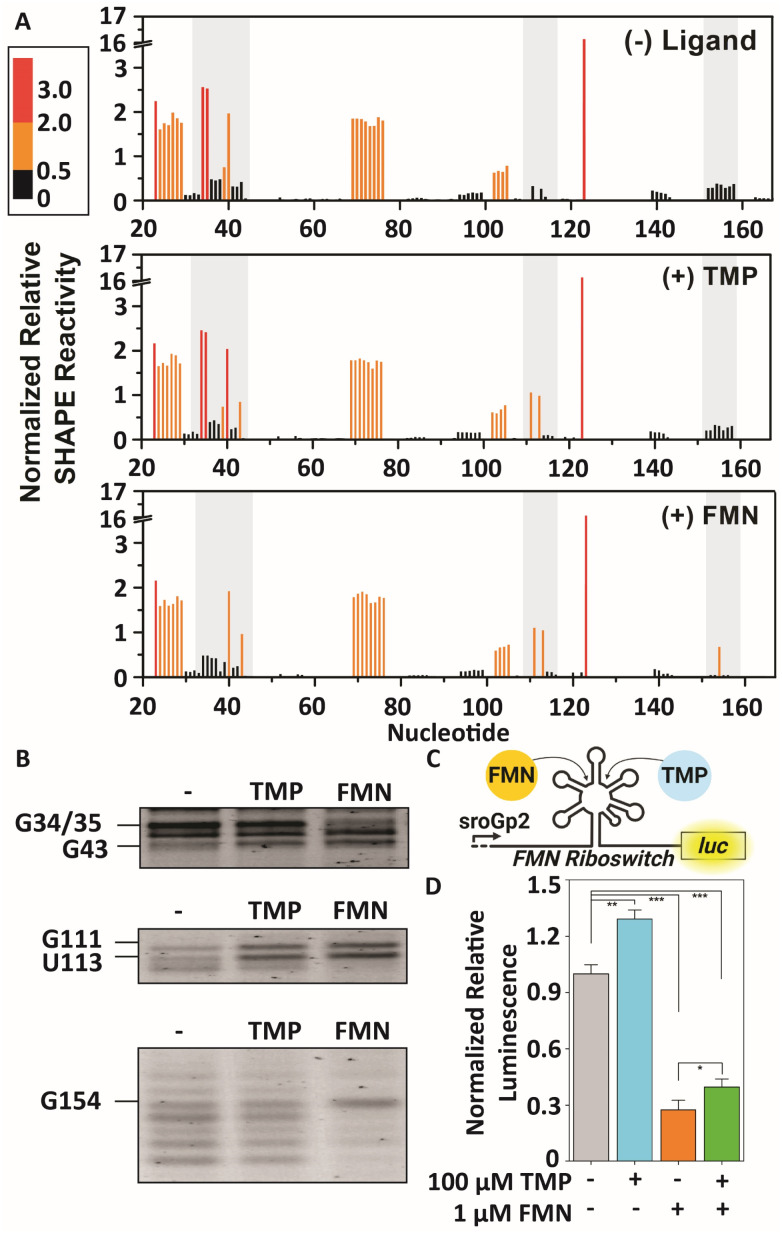
TMP binding to the FMN riboswitch RNA. (A) Quantification of SHAPE reactivity of the FMN riboswitch in the absence (−) or presence (+) of TMP or FMN. Quantified signals were based on triplicate measurements (Fig. S5†) and corrected for loading differences. Columns are colored by nucleotide SHAPE reactivities (see scale). Black bars are considered not reactive (<0.5), whereas red bars are considered highly reactive (>2.0). (B) Regions of the PAGE of the SHAPE experiment highlighted in (A) that show ligand-induced reactivity changes. (C) Schematic illustration of the IVTT assay using FMN and TPP as natural ligands. (D) Normalized relative luminescence signals observed when psroGp2-FMN-luc and psroGp2-luc were incubated with indicated ligands in IVTT assays. Error bars represent standard deviations based on triplicate measurements. Statistical significance was examined using an unpaired two-tailed Student's *t*-test (*p < 0.05; **p < 0.01; ***p < 0.001).

After verifying binding of TMP probe 1 to the FMN riboswitch, we proceeded to examine interactions between unmodified metabolite TMP and the riboswitch. Therefore, TMP binding was studied with Selective 2′-Hydroxyl Acylation analyzed by Primer Extension (SHAPE) analysis and compared to FMN.^3,56–58^ FMN and TMP were separately incubated at 100 μM or 500 μM respectively with the riboswitch at temperatures between 4–37 °C for 30 minutes, after which 1M7 (ref. 59) was used to acylate the RNA. The concentration of FMN was based on previous experiments,^56^ whereas the chosen concentration of TMP ensured capturing potential interactions with the FMN RNA. The RNA was purified by precipitation, reverse transcribed using a fluorescently labeled primer and analyzed using PAGE. Only at lower incubation temperatures significant SHAPE differences were observed for TMP, hinting that the observed TMP interactions are likely weaker and more dynamic than the interactions of FMN (Fig. 5A, B and S5, S6A, B†). Similar enhanced nucleotide reactivity patterns were observed for FMN and TMP at nucleotides in the region at G43 and U113 (Fig. 5A, B and S5†). These interactions are reported to be important for recognition of the phosphate group of FMN by the RNA aptamer.^54,60^ Additionally, G34 became less reactive and U153 more reactive upon binding of FMN compared to binding of TMP. U153 is reported to be expulsed out of the aptamer upon binding of the isoalloxazine ring of FMN. This is a critical conformational change in the J6/1 joining region for ligand binding and turning off expression of the downstream gene,^54,60^ which is notably not observed for TMP (Fig. 5A and B).

### Effect of TMP on gene expression using an *in vitro* transcription/translation system

To examine if the metabolite TMP exerts a potential effect on gene expression through interacting with the FMN riboswitch, we constructed an *in vitro* transcription/translation (IVTT) assay using an *E. coli* extract system based on earlier work from Pedrolli *et al.*^61^ (Fig. 5C). A plasmid DNA template was constructed that encodes for a firefly luciferase gene that is under control of the natural promotor sroGp2 and FMN riboswitch sequence of *ribB* from *E. coli* (psroGp2-FMN-luc). As a control, a plasmid encoding for the same promotor and firefly luciferase gene was produced without the riboswitch to exclude other effects (psroGp2-luc).^61^ IVTT reactions were performed using 7.5 nM plasmid and luminescence signals were measured for 2 hours (Fig. 5C, D and S7A–D†).

At 1 μM FMN the luminescence signal was inhibited by approximately 74% ± 5.1% (Fig. 5D and S7A–C†). To test the effect of TMP a markedly higher concentration of 100 μM was used to compensate for the potentially weaker binding observed by SHAPE. Surprisingly, TMP increased the luminescence signal with approximately 29% ± 4.8% (Fig. 5D and S7A–C†) instead of reducing it like FMN. The lack in signal reduction is in line with the SHAPE data that showed that TMP seems to interact with the phosphate binding site of the FMN riboswitch, but does not induce a conformational change of the riboswitch that results in inhibition of gene expression. To examine if TMP can compete with FMN for riboswitch binding, both ligands were incubated simultaneously. Interestingly, in this case the luminescent signal was only inhibited by 60% ± 3.9% (Fig. 5D and S7A–C†). This reduced inhibition compared to incubation with FMN only implies that TMP and FMN both compete for binding to the FMN riboswitch and have inverse effects on the expression level. As a further control, the experiment was repeated with 100 μM thiamine, the non-phosphorylated counterpart of TMP (Fig. 1B). In this case, no effect on luciferase expression was observed, hinting at the importance of the predicted phosphate interaction in the riboswitch aptamer (Fig. S7D†).

All data combined, it appears that TMP can selectively interact with the earlier well-characterized FMN riboswitch,^15,54,60,61^ albeit with lower affinity than its known cognate ligand FMN (Fig. S6A and B†).

## Conclusions

In summary, a TMP photoaffinity probe was synthesized and used to perform chemotranscriptomic profiling of TMP on total RNA from bacteria. After enrichment and RNA sequencing, TMP probe 1 appeared to bind to the *ribB* transcript that includes the FMN riboswitch. Validation with RT-qPCR and competition experiments suggest that probe 1 selectively binds in the aptamer domain. Using a termination assay, the bindings sites were obtained with nucleotide resolution. Subsequently, the unmodified metabolite TMP was investigated on interacting with the FMN riboswitch using SHAPE and IVTT. TMP induced a conformational change in the FMN riboswitch aptamer and increased gene expression levels in an IVTT assay. These results hint at a possible biological role of TMP that needs to be investigated further, considering the high micromolar concentration present in bacteria,^62^ in particular, when compared to FMN, which is reported to be present in low micromolar concentrations.^63^ TMP probe 1 appeared to interact with other transcripts as well, of which the significance needs to be further explored. Moreover, we believe that this study emphasizes the potential power of photoaffinity labeling to identify small molecule binding sites within the transcriptome.

## Data availability

ESI data have been included in the ESI. RNA sequencing data for this article are at Gene Expression Omnibus (GEO) and can be obtained by using the article title.†

## Author contributions

S. C. synthesized all compounds and performed the biochemical assays. C. F. M. optimized part of the synthesis route and performed the docking study. A. S. assisted in synthesizing the compounds and visualized the docking study results. D. A. L. v. d. H. analysed the sequencing data. W. A. V. designed the project and supervised experiments. S. C. and W. A. V. wrote the paper with input from all authors.

## Conflicts of interest

There are no conflicts to declare.

## Supplementary Material

SC-OLF-D4SC06189F-s001
