# Supplementary material for: Chemotranscriptomic profiling with a thiamine monophosphate photoaffinity probe
Source: Chem Sci. 2025 Feb 11;16(11):4725–31. doi: 10.1039/d4sc06189f (PMC11831223; doi:10.1039/d4sc06189f)
Supplement: SC-016-D4SC06189F-s001 [file SC-016-D4SC06189F-s001.pdf]

Supplementary Information (SI) for Chemical Science.
This journal is © The Royal Society of Chemistry 2025

Supplementary Information

Chemotranscriptomic Profiling with a Thiamine Monophosphate Photoaffinity Probe

Stefan Crielaard, Casper F. M. Peters, Alexandar Slivkov, Daphne A. L. van den Homberg and Willem A. Velema*
Institute for Molecules and Materials, Radboud University Nijmegen, Nijmegen, The Netherlands
*To whom correspondence should be addressed: willem.velema@ru.nl

Table of Contents

SUPPLEMENTARY TABLE S1 ..... **2**

SUPPLEMENTARY FIGURES **4**

Supplementary Figure S1: Photoaffinity Labeling of the FMN Riboswitch with probe **1** using PAGE 4

Supplementary Figure S2: Photoaffinity Labeling of the TPP Riboswitch with probe **1** using PAGE 5

Supplementary Figure S3: Reverse Transcription Termination Assay using **1** and FMN Riboswitch RNA 6

Supplementary Figure S4: Molecular Docking Predictions of FMN and Probe **1** in the FMN Riboswitch 7

Supplementary Figure S5: Denaturing PAGE after SHAPE with FMN or TMP in the FMN Riboswitch 8

Supplementary Figure S6: Concentration-dependent differences in SHAPE patterns of the FMN riboswitch after incubating with different concentrations of TMP or FMN. 9

Supplementary Figure S7: IVTT assay with FMN, TMP, Both Ligands or Thiamine 10

EXPERIMENTAL SECTION **11**

BIOCHEMICAL EXPERIMENTS **11**

Culturing Bacteria and RNA extraction 11

Photoaffinity Labeling in Total RNA Extract 11

Polymerase Chain Reaction and *In Vitro* Transcription of FMN Riboswitch RNA 12

Photoaffinity labeling of the FMN and TPP Riboswitch *in vitro* 12

Reverse Transcription Termination Assay 13

Molecular Docking 13

Selective 2' Hydroxyl Acylation analyzed by Primer Extension 14

Construction of psroGp2-FMN-luc and psroGp2-luc 14

In Vitro Transcription/Translation Assay 16

SYNTHESIS, PURIFICATION AND ANALYSIS OF COMPOUNDS **17**

SPECTRA **22**

REFERENCES **80**

SUPPLEMENTARY TABLE S1

**Genes:**	**Biological function:**
rnpB	RNase P catalytic RNA component
prs	Ribose-phosphate diphosphokinase
ribB	3,4-dihydroxy-2-butanone 4-phosphate, a precursor in the biosynthesis of riboflavin (FMN riboswitch in the 5′ UTR)
yihD	DUF1040 domain-containing protein YihD
yejA	Periplasmic binding component of a putative ATP-dependent oligopeptide uptake system
rpmF	50S ribosomal subunit protein L32
pepN	Aminopeptidase N
dppA	Periplasmic binding protein of a dipeptide ABC transport system
rrfF	5S ribosomal RNA
rrfD	5S ribosomal RNA
kefB	Glutathionine-regulated potassium efflux system
aldA	Aldehyde dehydrogenase A
ykgG	DUF162 domain-containing lactate utilization protein
treF	Cytoplasmic trehalase
glnH	Periplasmic binding component of an L-glutamine ABC transport system
hypB	Hydrogenase isoenzymes nickel incorporation protein
trpE	Anthranilate synthase subunit TrpE
sdhC	One of two membrane proteins part of the four subunit succinate dehydrogenase enzyme
rrfG	5S ribosomal RNA
lrp	DNA-binding transcriptional dual regulator Lrp
psd	Phosphatidylserine decarboxylase proenzyme
mug	Stationary phase mismatch/uracil DNA glycolase
ushA	Periplasmic UDP-sugar hydrolase (5′-nucleotidase)
yiiX	Putative lipid-binding hydrolase
rrfH	5S ribosomal RNA
nrfC	Nitrite reductase
rsxD	Reducer of SoxR
metI	Bifunctional aspartokinase/homoserine dehydrogenase 2
purL	Phosphoribosylformylglycinamidine syntase
yfeO	Putative ion-transporter protein
ssrS	6S RNA involved in stationary phase regulation of transcription by the sigma70-holoenzyme
parE	DNA topoisomerase 4 subunit B
sdhD	Succinate dehydrogenase hydrophobic membrane anchor subunit
frdD	Fumarate reductase
plsY	Putative glycerol-3-phosphate acyltransferase
trmO	tRNA m6t6A37 methyltransferase
rrfA	5S ribosomal RNA

Continuing Supplementary Table S1:

Genes:	Biological function:
purL	Phosphoribosylformylglycinamidine syntase
yfeO	Putative ion-transporter protein
ssrS	6S RNA involved in stationary phase regulation of transcription by the sigma70-holoenzyme
parE	DNA topoisomerase 4 subunit B
sdhD	Succinate dehydrogenase hydrophobic membrane anchor subunit
frdD	Fumarate reductase
plsY	Putative glycerol-3-phosphate acyltransferase
rrfA	5S ribosomal RNA
trmO	tRNA m^6t^6A37 methyltransferase
fadB	Multifunctional enoyl-CoA hydratase, 3-hydroxyacyl-CoA epimerase, Δ^3-*cis*- Δ^2-*trans*-enoyl-CoA isomerase, L-3-hydroxyacyl-CoA dehydrogenase
yeiQ	Putative oxidoreductase YeiQ
corA	$Nickel^{2+}$/$Cobalt^{2+}$/$Magnesium^{2+}$ transporter
rrfB	5S ribosomal RNA
frmA	S-(Hydroxymethyl)glutathione dehydrogenase
yggX	Putative Fe^{2+}-trafficking protein
fau	Putative 5-formyltetrahydrofolate cyclo-ligase
ynfM	Putative transporter
ymcF	Cold shock protein
yfbR	dCMP phoshohydrolase
sgrR	DNA-binding transcriptional dual regulator
insB5	IS1 protein InsB
insB6	IS1 protein InsB
rssA	Putative patatin-like phospholipase
prkB	Putative phosphoribulokinase
ilvB	Acetohydroxy acid synthase I subunit
hybA	Hydrogenase 2 iron-sulfur protein
yeeJ	Inverse autotransporter adhesin

Supplementary Table S1. List of all significantly enriched genes from the sequencing results displayed in **Figure 3A** with biological function included. Biological functions were obtained from RegulonDB v12.0[1].

SUPPLEMENTARY FIGURES

Supplementary Figure S1: Photoaffinity Labeling of the FMN Riboswitch with probe 1 using PAGE

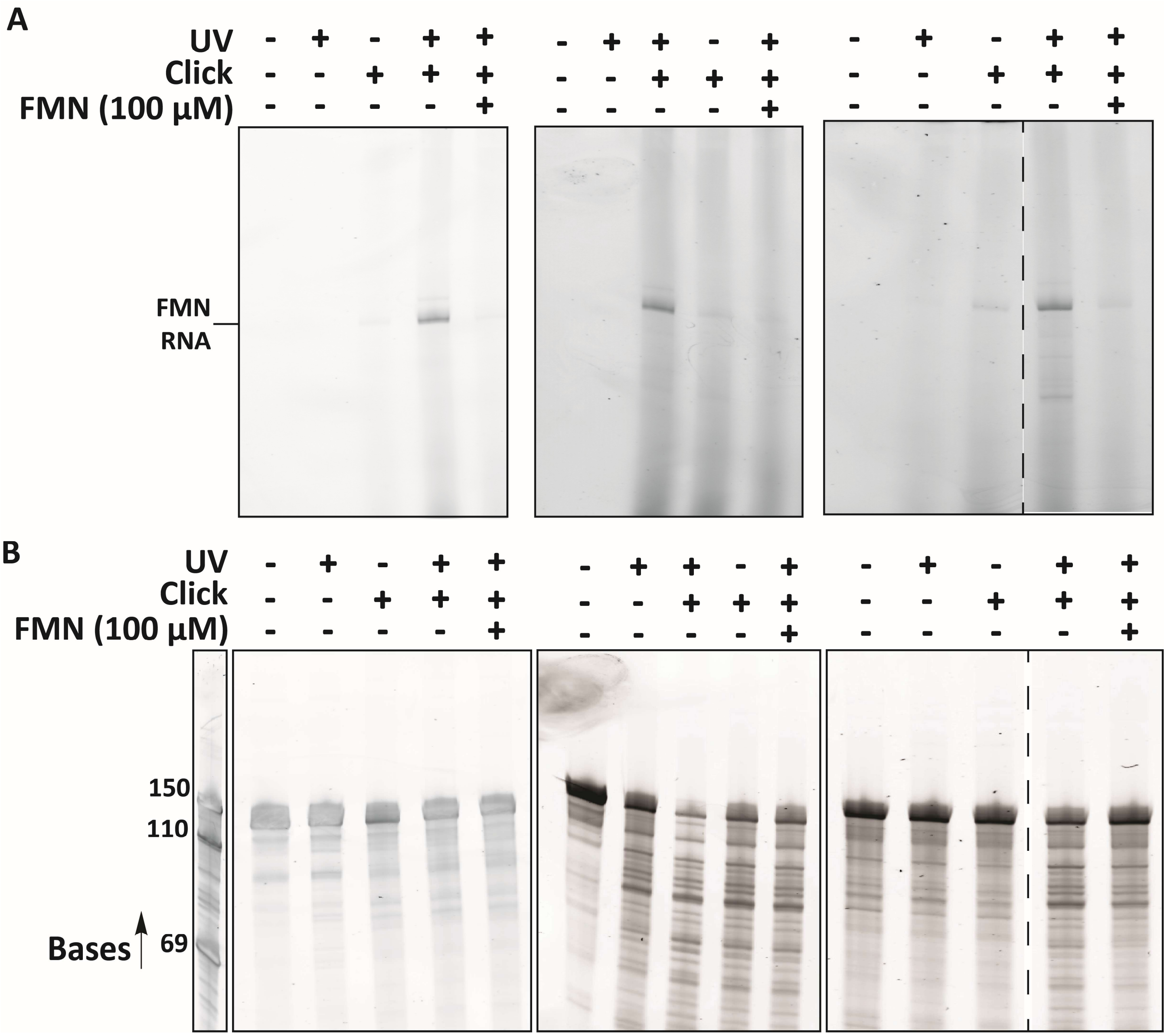

Supplementary Figure S1. The efficiency and competition experiment of 25 µM TMP probe **1**. (A) Fluorescence imaging of denaturing PAGE gels to show RNA labeling of *in vitro* transcribed FMN riboswitch RNA. (B) SYBR Gold RNA staining of the same denaturing PAGE gels to show presence of equal amounts of RNA in every sample. On the left side, an RNA ladder is included to further indicate labeling of RNA of the proper size. The FMN RNA is expected to be 147 nucleotides.

Supplementary Figure S2: Photoaffinity Labeling of the TPP Riboswitch with probe 1 using PAGE

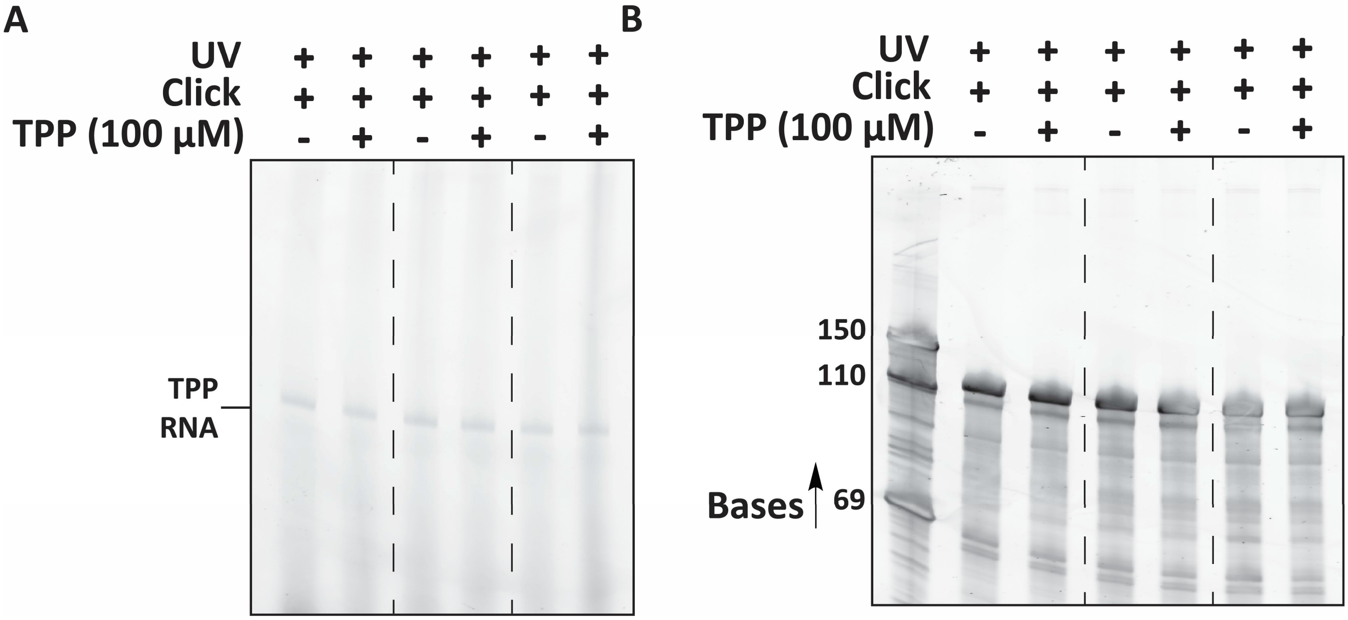

Supplementary Figure S2. Non-selective labeling of the TPP riboswitch aptamer with 25 µM TMP probe **1**. (A) Fluorescence imaging of denaturing PAGE gels to show non-selective RNA labeling of *in vitro* transcribed TPP riboswitch RNA. (B) SYBR Gold RNA staining of the same denaturing PAGE gels to show presence of equal amounts of RNA in every sample. On the left side, an RNA ladder is included to further indicate labeling of RNA of the proper size. The *in vitro* labeling and competition experiment were both performed in triplicates. The TPP RNA is expected to be 138 nucleotides.

Supplementary Figure S3: Reverse Transcription Termination Assay using 1 and FMN Riboswitch RNA

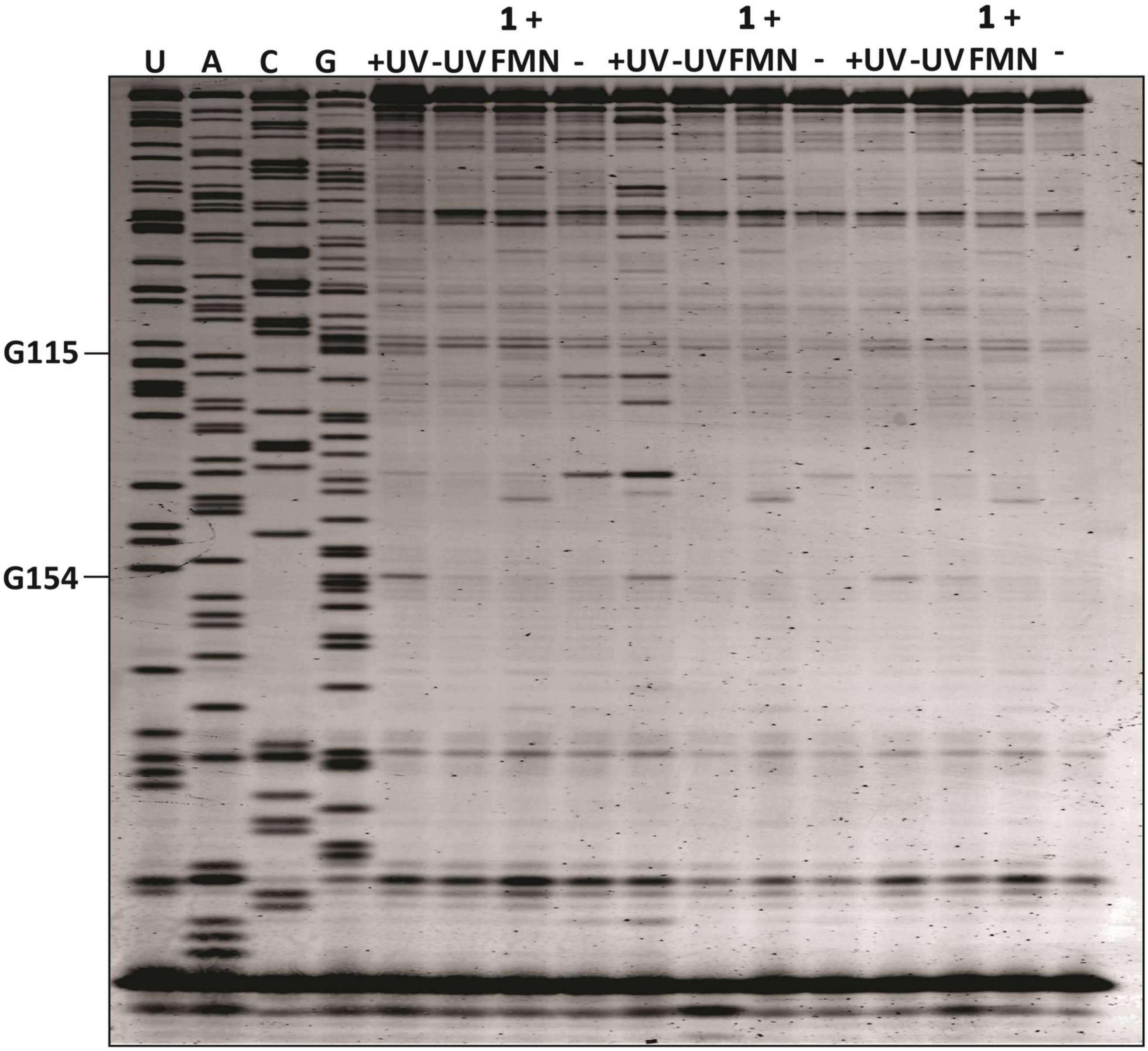

Supplementary Figure S3. Reverse Transcription (RT) Termination Assay to localize photocrosslinks of **1** in FMN Riboswitch RNA. Fluorescence imaging of the denaturing sequencing PAGE gel showing triplicate measurements per indicated condition. Additional stops that are observed in the '+UV' samples were compared to control samples. Gel lanes of indicated conditions were quantified, averaged and corrected for loading differences observed in the 5'-SHAPE cassette regions. On the left side, sequencing lanes were included by using dideoxynucleotides. '+UV' shows results when probe was incubated with RNA and UV irradiated. '-UV' shows results when the probe was incubated with RNA without UV irradiation. '**1** + FMN' shows results when **1** and 100 µM of FMN are competing for binding the FMN riboswitch, after which UV irradiation is performed. '-' means that no probe was included in the experiment, but the RNA was UV irradiated. On the side of the gel are nucleotides indicated that are in proximity to reverse transcription stops. Further experimental details can be found in the section 'Reverse Transcription Termination Assay'.

Supplementary Figure S4: Molecular Docking Predictions of FMN and Probe 1 in the FMN Riboswitch

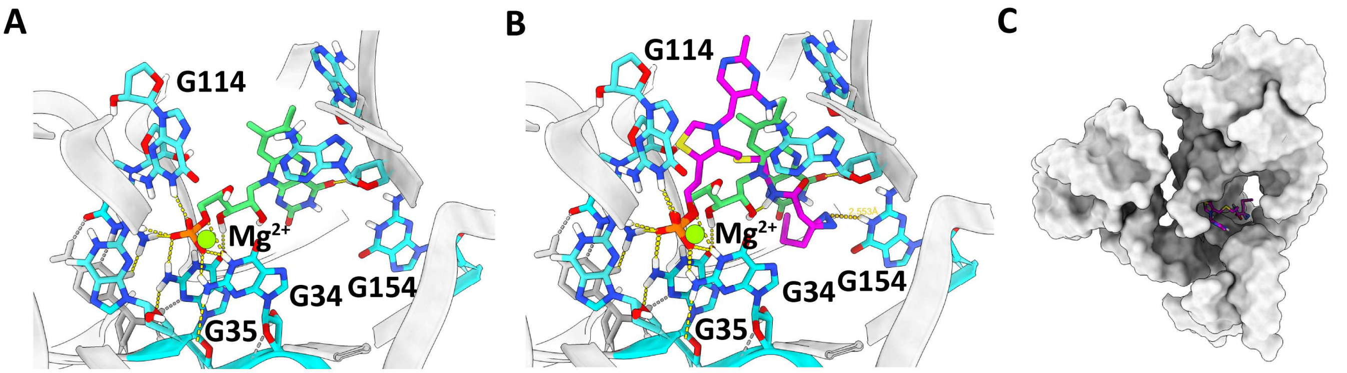

Supplementary Figure S4. Molecular docking predictions of FMN and TMP in the FMN riboswitch of *F. Nucleatum* (PDB ID: 3F2Q)[2,3]. (A) Docked binding site where FMN binds in the FMN riboswitch. FMN is indicated in green. Nucleotides of the RNA are indicated in grey or highlighted in blue. (B) Overlay of the docked binding sites where probe **1** and FMN bind in the FMN riboswitch. The probe is indicated in pink, FMN in green. Nucleotides of the RNA are indicated in grey or highlighted in blue. The phosphate functional group of TMP probe **1** and FMN are predicted to be positioned in a similar manner. Highlighted nucleotides in (A) and (B) are annotated according to FMN riboswitch numbering of *ribD* from *Bacillus Subtilis.*[2] (C) Docking of TMP probe **1** in the FMN riboswitch. Probe **1** is indicated in pink, the RNA in grey.

Supplementary Figure S5: Denaturing PAGE after SHAPE with FMN or TMP in the FMN Riboswitch

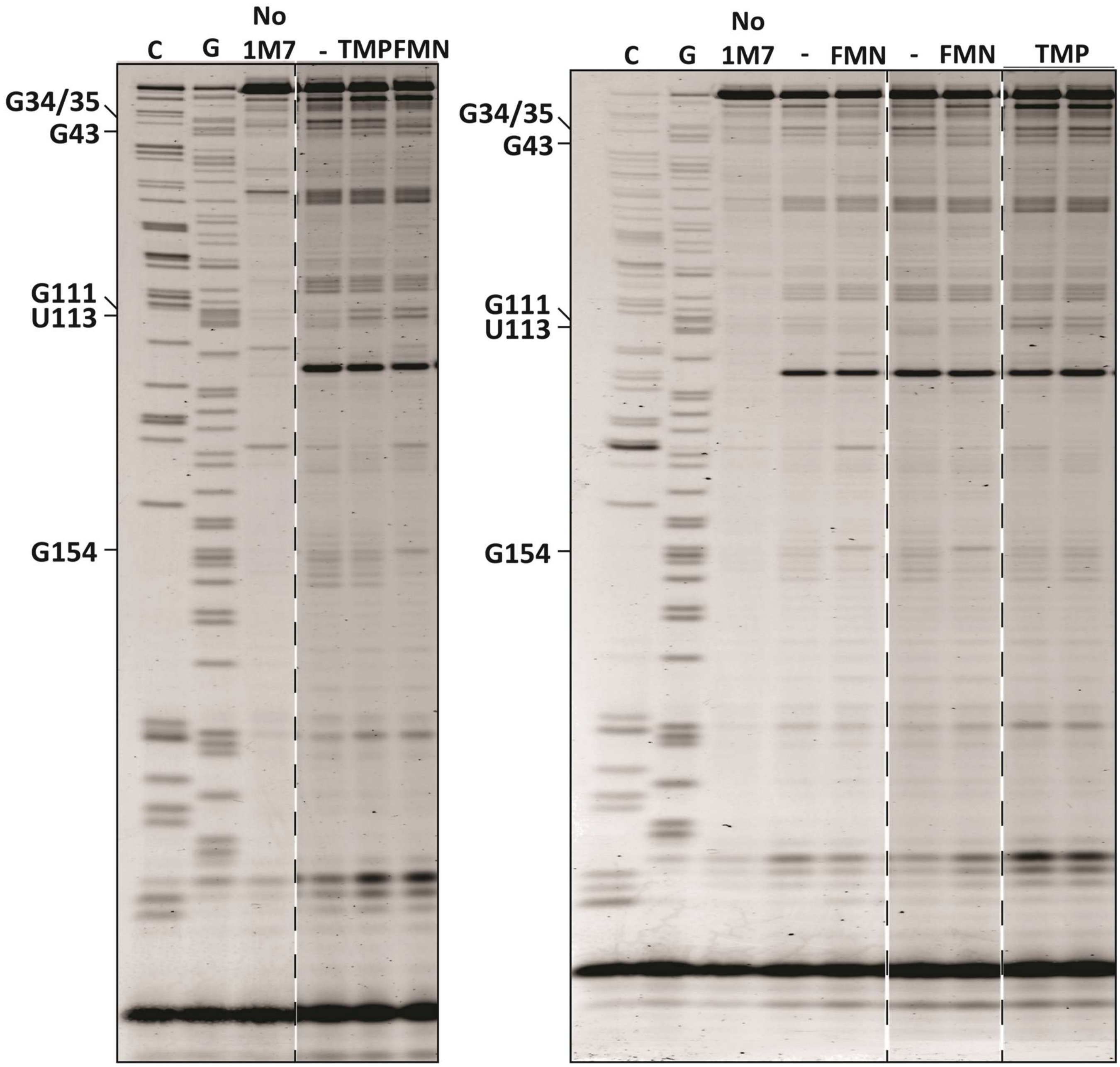

Supplementary Figure S5. SHAPE assay to show reactivity of nucleotides induced by binding of 500 µM TMP or 100 µM FMN in the FMN riboswitch RNA. Fluorescence imaging of the denaturing sequencing PAGE gel showing triplicate measurements per indicated condition. Stops indicate nucleotides with enhanced acylation reactivity. Gel lanes of indicated conditions were quantified, averaged and corrected for loading differences observed in the 5′-SHAPE cassette regions. On the left side, sequencing lanes were included by using selected dideoxynucleotides. ‘No 1M7’ shows the RT efficiency when no 1M7 is included before RT. ‘-’ shows results when no ligand was incubated with the RNA. ‘TMP’ shows results when TMP was incubated with the RNA. ‘FMN’ shows results when FMN is combined with the FMN riboswitch. On the side of the gel are nucleotides indicated that are in proximity to induced folding difference regions in the FMN riboswitch RNA. Further experimental details can be found in the section ‘Selective 2′ Hydroxyl Acylation analyzed by Primer Extension’.

Supplementary Figure S6: Concentration-dependent differences in SHAPE patterns of the FMN riboswitch after incubating with different concentrations of TMP or FMN

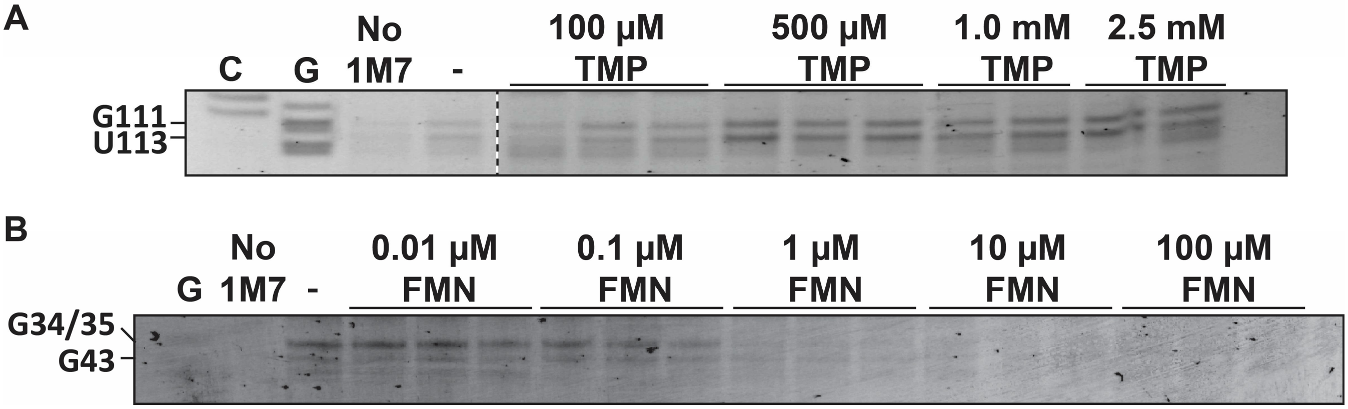

Supplementary Figure S6. SHAPE experiment to highlight reactivity differences of significantly affected nucleotides of the FMN riboswitch after incubation with varying concentrations of TMP or FMN. (A) Change in reactivity pattern of G111 and U113, as these nucleotides most significantly changed (Figure 5A and Supplementary Figure S5), when FMN riboswitch RNA is incubated with 100-2500 µM TMP. The increased signal in the highlighted gel lanes at 500 µM TMP and higher concentrations indicates ligand interaction with the riboswitch. On the left side, sequencing lanes were included by using selected dideoxynucleotides. 'No 1M7' shows the RT efficiency when no 1M7 is included before RT. '-' shows results when no ligand was incubated with the RNA. (B) Change in reactivity pattern of G34/35, as these nucleotides most significantly changed (Figure 5A and Supplementary Figure S5) when FMN riboswitch RNA is incubated with 0.01-100 µM FMN. The decreased signal in the highlighted gel lanes at 1 µM FMN and higher concentrations indicates ligand-induced folding of the riboswitch. On the left side, sequencing lanes were included by using selected dideoxynucleotides. 'No 1M7' shows the RT efficiency when no 1M7 is included before RT. '-' shows results when no ligand was incubated with the RNA. These results suggest that the interactions between TMP and the FMN riboswitch are weaker compared to those between the riboswitch and FMN.

Supplementary Figure S7: IVTT assay with FMN, TMP, Both Ligands or Thiamine

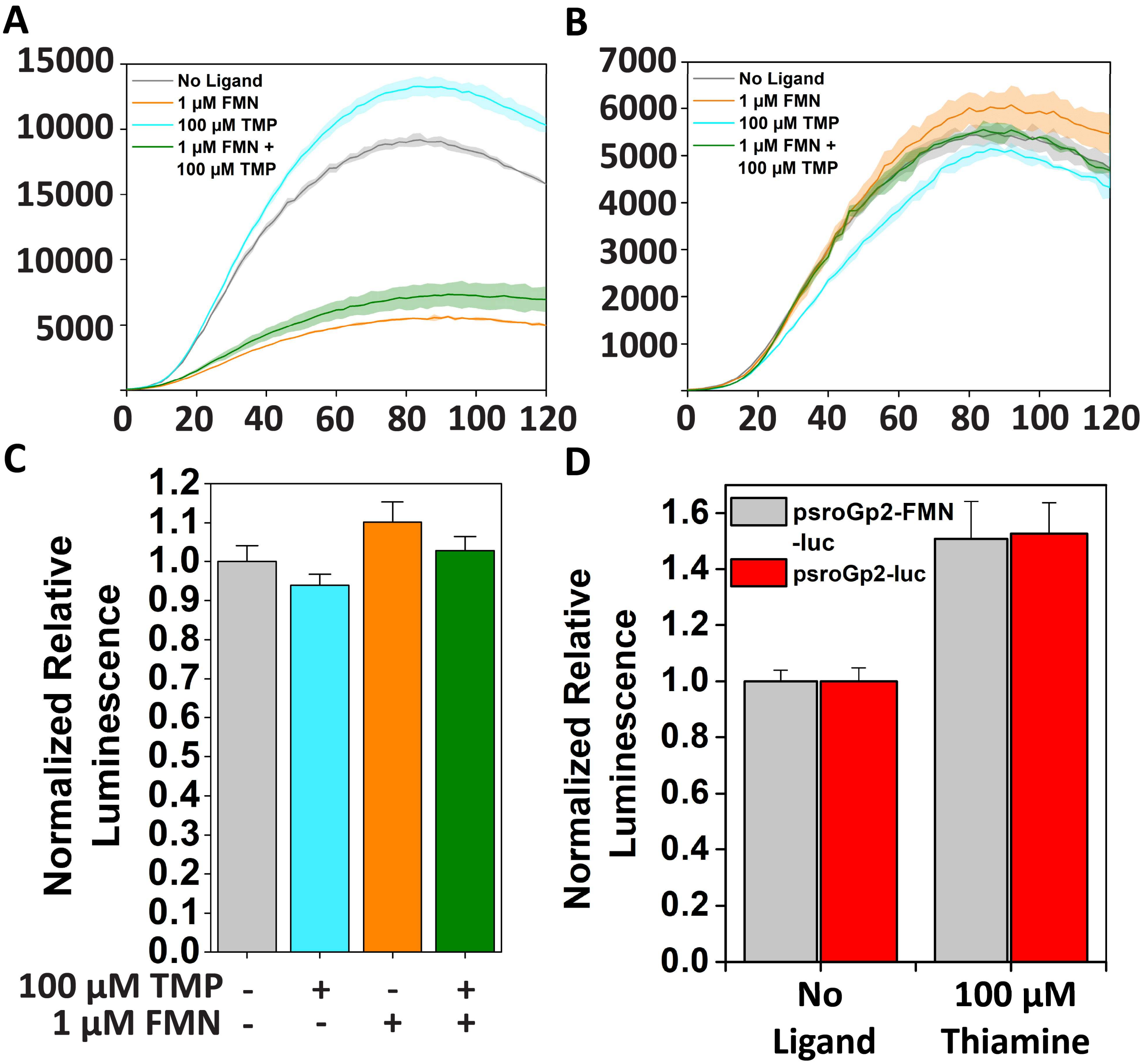

Supplementary Figure S7. Luminescence-based IVTT assays showing that the interactions of FMN and TMP affect the gene expression levels of genes under control of the FMN riboswitch. (A) Relative luminescence signals measured for psroGp2-FMN-luc expression upon incubating with no ligand or indicated amounts of FMN, TMP or FMN and TMP simultaneously. (B) Relative luminescence signals measured for psroGp2-luc expression upon incubating with no ligand, or indicated amounts of FMN, TMP or FMN and TMP simultaneously. (C) Normalized relative luminescence signals after IVTT reactions with psroGp2-luc and the different indicated ligands. The peak signals were used to compare and normalize results. (D) Normalized relative luminescence signals after performing IVTT reactions with psroGp2-FMN-luc and psroGp2-luc with no ligand or 100 µM thiamine. No effect was observed for thiamine.

EXPERIMENTAL SECTION

BIOCHEMICAL EXPERIMENTS

All reagents used were purchased from commercial suppliers and were used according to manufacturer's instructions. Oligonucleotide sequences were ordered from Integrated DNA Technology (IDT). *Escherichia coli* (*E. coli*) CS1562 (CGSC#: 6911) was a gift from prof. Arnold Driessen, University of Groningen.

Culturing Bacteria and RNA extraction

Escherichia coli CS1562 (tolC6:tn10) was cultured on sterile Luria Broth (LB) (5 g/L yeast extract, 10 g/L tryptone and 10 g/L sodium chloride) agar plates supplemented with tetracycline (25 µM) at 37 °C. Colonies were picked and grown in liquid LB medium supplemented with tetracycline (25 µM) at 37 °C.

For RNA extractions, bacteria were grown to OD_{600} of 0.8. Bacteria were spun down, LB supplemented with antibiotic was removed, and the bacteria were washed once with PBS (1x, pH 7.2). Bacteria were resuspended in lysozyme (100 µL, 0.4 mg/mL in 10 mM Tris/EDTA (pH) 8.0) and incubated for 20 minutes. RNA was extracted from bacteria using the Quick-RNA™ MiniPrep kit. The recommended DNase I step was incorporated to ensure removal of DNA during RNA purification. The purified RNA was eluted in NF-water (100 µL) and quantified by NanoDrop. RNA extracts were considered sufficiently pure when both A_{260}/A_{230} and A_{260}/A_{280} ratios were above 2.0.

Photoaffinity Labeling in Total RNA Extract

Total RNA (30 µg in 57 µL NF-water) from *E. coli* CS1562 was incubated at 75 °C for 5 minutes and stored on ice for 3 minutes. Three-fold concentrated folding buffer (33 µL, 333 mM NaCl, 33 mM $MgCl_2$, 300 mM HEPES (pH 8.0)) was added to the denatured RNA and the samples were incubated at 37 °C for 10 minutes. NF-water (5 µL) or FMN (2 mM, 5 µL) was added and the samples were incubated at 37 °C for 20 minutes. Probe **1** (1 mM, 5 µL), control probe **12** (1 mM, 5 µL) or DMSO (5 µL) was added to the samples to obtain a total volume of 100 µL. Every mixture contained the same percentage of DMSO. The RNA samples were incubated at 37 °C for 30 minutes. UV irradiation was performed with 365 nm light in a UV Stratalinker for 30 minutes. The RNA was isolated by ethanol precipitation[4]. RNA pellets were washed with 70% (v/v) ethanol and dissolved in 54 µL NF-water. The isolated RNA was incubated at 65 °C for 5 minutes and stored on ice for 3 minutes. A ten-fold concentrated click mixture (6 µL) was added to each sample to obtain final concentrations of 0.5 mM $CuSO_4$, 5.0 mM sodium ascorbate, 2.5 mM tris-(3-hydroxypropyltriazolylmethyl)amine and 0.2 mM disulfide biotin azide. The click reactions were performed at 37 °C with agitation at 500 rpm for 90 minutes. Total RNA was isolated by ethanol precipitation[4], dissolved in 100 µL binding buffer (0.5 M NaCl, 20 mM Tris-HCl (pH 7.5), 1 mM EDTA), incubated at 65 °C for 5 minutes and sequentially at 4 °C for 3 minutes. Streptavidin magnetic beads (NEB, 25 µL) were added to each sample and the samples were incubated in an overhead rotator at room temperature for 90 minutes. A fraction (4 µL) of input RNA was stored at -20 °C to use during downstream RT-qPCR. Beads were washed three times using washing buffer (0.5 M NaCl, 20 mM Tris-HCl (pH 7.5), 1 mM EDTA, 0.2% (v/v) Tween-20) and three times with low-salt buffer (0.15 M NaCl, 20 mM Tris-HCl (pH 7.5), 1 mM EDTA) using a magnetic rack. RNA was eluted by resuspending the beads in DTT (50 mM, 20 µL). Eluted RNA (2 µL) per sample was used for one-step RT-qPCR (Luna, NEB) using an annealing temperature of 58 °C, 20 µL reaction volume and 40 cycles. The used primers are depicted in **Supplementary Table S2**. The relative fold enrichment of *rnpB*, *prs* and *ribB* were calculated by comparing the differences in Ct values before and after pulldown after correcting the Ct values of the gene of interest using the housekeeping gene *cysG*. The values were normalized over the observed signal for DMSO control samples. The error bars represent standard deviations based on three biological replicates, each consisting of two technical replicates. Statistical significance was defined by unpaired two-tailed Student's *t* tests.

For sequencing, eluted RNA was cleaned up using RNA Clean and Concentrator™-5 (Zymo) and dissolved in NF-water (10 µL) with 2.5 ng/µL RNA as minimal concentration measured using the QuBit RNA High Sensitivity kit (Invitrogen). For the DMSO samples, the experiment was performed with three times the amount of RNA sample

to obtain enough RNA after cleaning up the samples. Briefly, the KAPA RNA HyperPrep Kit with RiboErase (Roche) was used for the library preparation. Samples were sequenced using the Illumina NextSeq2000 (2x 59 bp) by the Sequencing Facility FNWI Science faculty Radboud University Nijmegen. Sequencing reads were aligned to the *E. coli* K12 MG1655 genome using Bowtie2[5] and summarized using FeatureCounts[6]. First, low coverage genes were filtered out (counts per million <1) and then enriched genes were analyzed using EdgeR[7]. Differentially expressed genes were visualized in a volcano plot using a cutoff value of Log2 Fold Change of 0.5 and Pvalue of 0.05.

**Primers**	**Sequence (5′ to 3′)**
rnpB Forward Primer	ATA AGC CGG GTT CTG TCG TG
rnpB Reverse Primer	GTA AAC TCC ACC CGG AGC AA
prs Forward Primer	CCA GCA TAC CTG ACA GGG TC
prs Reverse Primer	CTC ACC CGA TCT TCT CTG GC
ribB Forward Primer	GTA ACG ATT CTG TCG GGC AT
ribB Reverse Primer	TAC CAG AAT CAG GGC AGT CT
cysG Forward Primer	TTG TCG GCG GTG GTG ATG TC
cysG Reverse primer	ATG CGG TGA ACT GTG GAA TAA ACG

Supplementary Table S2. DNA sequences of the used primers during One-Step RT-qPCR for selected amplicons of *rnpB*, *prs* and *ribB* (genes of interest), and *cysG* (housekeeping gene) to quantify pulldown enrichments.

Polymerase Chain Reaction and *In Vitro* Transcription of FMN Riboswitch RNA

To obtain the FMN riboswitch RNA, the same polymerase chain reaction, including the sequences, and *in vitro* transcription methods were applied as described in Crielaard *et al.* (2022)[8]. For the photoaffinity labeling experiments, the FMN riboswitch aptamer RNA was used. The FMN riboswitch RNA was inserted in between 5′ and 3′ SHAPE cassettes for SHAPE and Reverse Transcription Termination Assays.[8,9]

The *thiM* TPP riboswitch that was used for *in vitro* photoaffinity labeling studies was inserted in between the same structure cassettes that were used for the FMN riboswitch. The TPP riboswitch RNA was produced as described in Crielaard *et al.* (2022)[8] using the same primer set that was used when the FMN riboswitch was inserted in between structure cassettes. The DNA sequence that was used to produce the TPP RNA was obtained from Edwards and Ferré-D'Amaré *et al* (2014)[10]:

5′ - GCGGAATTCTAATACGACTCACTATAGGGCCTTCGGGCCAAGGACTCGGGGTGCCCTTCTGCGTGAAGGCTGAGAAAT
ACCCGTATCACCTGATCTGGATAATGCCAGCGTAGGGAAGTTCTCGATCCGGTTCGCCGGATCCAAATCGGGCTTCGGTCC
GGTTC - 3′

Photoaffinity labeling of the FMN and TPP Riboswitch *in vitro*

FMN RNA or TPP RNA (10 pmol) was combined with NF-water (5 µL), incubated at 95 °C for 5 minutes and stored on ice for 3 minutes. Three-fold concentrated folding buffer (33 µL, 333 mM NaCl, 33 mM $MgCl_2$, 300 mM HEPES (pH 8.0)) was added and the RNA was incubated at 37 °C for 10 minutes. FMN (1 µL, 1 mM), TPP FMN (1 µL, 1 mM) or NF-water (1 µL) was added to the RNA and the samples were incubated in the dark at 37 °C for 20 minutes.

Probe **1** (0.5 µL, 1 mM) was added to every sample. The samples were incubated in the dark at 37 °C for 30 minutes. When photocrosslinking was required, the samples were irradiated with 365 nm light in a UV Stratalinker for 30 min. Otherwise, the samples were kept in the dark at 37 °C. Afterwards, the RNA was isolated by ethanol precipitation[4]. The RNA pellets were washed with 70% (v/v) ethanol and dissolved in NF-water (9 µL). The isolated RNA was incubated at 65 °C for 5 minutes and stored on ice for 3 minutes. A ten-fold click mixture (1 µL) was added to the RNA samples to obtain final concentrations of 0.5 mM $CuSO_4$, 5.0 mM sodium ascorbate, 2.5 mM tris-(3-hydroxypropyltriazolylmethyl)amine and 0.4 mM fluorescein-azide. The RNA was incubated in the dark at room temperature for 60 minutes. RNA was isolated by ethanol precipitation in a similar way as before. The RNA pellets were dissolved in urea (8M, 10 µL) and analyzed using denaturing 8% (v/v) polyacrylamide gel. Fluorophore labeling was visualized using fluorescence imaging (Typhoon Amersham). The gel was stained using SYBR Gold and visualized by fluorescence imaging (Typhoon Amersham). Labeling of the correct RNA size was confirmed by including an RNA ladder with known sizes. Quantification of the labeled RNA bands was performed *in triplo* using FiJi ImageJ[11] and selecting the labeled RNA bands. The gel lanes were corrected for background signal originating from the gel, averaged and normalized over the highest signal. The error bars represent standard deviations based on three biological replicates. Statistical significance was defined by unpaired two-tailed Student's *t* tests.

Reverse Transcription Termination Assay

Photoaffinity labeling of the FMN RNA was performed in a similar way as described in 'Photoaffinity labeling of the FMN Riboswitch *in vitro*' using 5 pmol FMN RNA. For '-', DMSO (3% (v/v)) was added instead of probe. For '-UV', no UV irradiation was performed. RNA was isolated by ethanol precipitation[4]. The RNA pellets were dissolved in NF-water (10 µL). The RNA was combined with 6 pmol FAM-Primer (5'- /56-FAM/GAA CCG GAC CGA AGC CCG -3'). To obtain sequencing lanes, untreated RNA (5 pmol) was incubated with FAM-primer (6 pmol). All samples were incubated at 65 °C for 5 minutes and stored on ice for 3 minutes. For all RT stop samples, dNTPs (2 µL, 10 mM), five-fold first-strand buffer (4 µL), and DTT (0.1 M, 1 µL) were added. To create a sequencing lane, RT was performed using a dNTP mix containing a 10:1 molar ratio for one selected ddNTP:dNTP (Jena Biosciences), whereas the other three dNTPs are in 1:1 molar ratio to the selected ddNTP. Superscript III was added to initiate all RT reactions. The reactions were incubated at 45 °C for 2 minutes, 55 °C for 60 minutes and 70 °C for 15 minutes. Alkaline hydrolysis was performed by adding 1 M NaOH (10 µL) and incubating at 95 °C for 10 minutes. The reactions were quenched using 1 M HCl. The cDNA was isolated by ethanol precipitation[4], cDNA pellets were dissolved in urea (8M, 5 µL) and loaded in a denaturing 8% polyacrylamide gel. Products were separated on a gel in 1x TBE (pH 8.3); 1000 V for 3.5 hours and 250 V for 16 hours. The gels were visualized by fluorescence imaging (Typhoon Amersham). The gel lanes were quantified using Fiji ImageJ[11]. A gel lane in the SHAPE cassette was used to correct for gel loading differences. The signals for '+UV' and '-UV' were averaged over biological triplicates and the signal intensities were normalized over the highest signal.

Molecular Docking

Docking was conducted on Molecular Operating Environment[12] (MOE 2015) using the 'crystal structure of the FMN riboswitch bound to FMN' (PDB ID: 3F2Q) published by Serganov *et al*. (2009)[3,13]. All preparatory steps were performed with default settings. The model was refined using the H- CUFIX forcefield[14,15] and partial charges were calculated and applied subsequently. Protonate3D was used to protonate the system and a 5000-step energy minimization was performed for the solvated system. Two-dimensional (2D-) structures of probe **1** and TMP were prepared in ChemDraw 22.2.0 ultra, converted into 3D-structures in MOE using QuickPrep and minimized in energy. After preparation of the RNA model and ligands, general docking function[16] was employed using the Triangle matcher docking method, London dG scoring function[17] and GBVI/WSA dG scoring function[18]. Both induced fit and rigid receptor were applied for refinement with 15 poses. The best docked orientation was selected based on the docking score and binding interactions. ChimeraX[19] was used to visualize the predicted structures. Only nucleotides that are predicted to interact with the ligands and are conserved in both the FMN riboswitch of *Bacillus Subtilis* wildtype and *Fusobacterium Nucleatum* were discussed and highlighted in the shown structures.[2]

Selective 2′ Hydroxyl Acylation analyzed by Primer Extension

FMN RNA (5 pmol) was combined with NF-water (6 µL), incubated at 95 °C for 5 minutes and stored on ice for 3 minutes. Three-times concentrated folding buffer (3.3 µL, 100 mM HEPES (pH 8.0), 33.3 mM $MgCl_2$ and 333 mM NaCl) was added to each RNA sample. The RNA was incubated at 37 °C for 10 minutes. The indicated ligand (100-2500 µM TMP, or 0.01-100 µM FMN) or NF-water was added to the RNA and the samples were incubated at 4 °C in the dark for 30 minutes. After incubation, 9 µL of folded RNA was added to 1 µL of 160 mM 1M7 (in DMSO) and incubated at indicated temperatures for 2 minutes. No-acylation control reactions were performed with DMSO (1 µL). The RNA was isolated by ethanol precipitation as described in 'Photoaffinity labeling of the FMN Riboswitch *in vitro*'. The pellets were dissolved in 10 µL NF-water. The reverse transcription, alkaline hydrolysis, precipitation and gel readout were performed in a similar way as described in 'Reverse Transcription Termination Assay'. The gel lanes were quantified using FiJi ImageJ[11]. A gel lane in the SHAPE cassette was used to correct for gel loading differences. The gel intensity patterns of the SHAPE samples of 'TMP' were compared to the SHAPE pattern observed for no ligand. Experiments were performed in biological triplicates.

Construction of psroGp2-FMN-luc and psroGp2-luc

psroGp2-FMN-luc was produced by Golden Gate assembly in a similar manner as described in Crielaard *et al* (2022)[8], after which that plasmid was digested and a fragment without encoding for the FMN riboswitch was inserted to create psroGp2-luc using the templates reported by Pedrolli *et al.* (2015)[20]. The used fragments to create pSroGp2-FMN-luc are reported in Crielaard *et al.* (2022)[8] or in **Supplementary Table S3**. All fragments were obtained in-house, except for the fragments sroGp2-FMN fragment and sroGp2 (obtained from Integrated DNA Technologies).

Briefly, vector fragment (100 ng) was mixed with the 5′ fragment, sroGp2-FMN fragment, luciferase fragment and the 3′+T500 fragment in a 1:3 molar ratio. Ligation reactions were performed in 15 µL total volume, including T4 ligase buffer (1x) and Esp3I and T4 ligase (both 1 µL). The reactions were incubated using the indicated ligation program.

Ligated plasmid mixture (2 µL) was mixed with competent TOP10F *E. coli* (200 µL).[21] The samples were mixed and left on ice for 30 minutes. The bacteria were transformed by performing a heat shock at 42 °C for 45 seconds, after which the bacteria were incubated on ice for 3 minutes. Bacteria were plated on LB plates containing 50 µg/mL ampicillin and grown at 37 °C overnight. Colonies were picked and grown in liquid LB containing 100 µg/mL ampicillin at 37 °C overnight. Plasmid DNA was isolated by QIAprep Spin Miniprep Kit (QIAGEN). Plasmid sequences were analyzed using agarose gel electrophoresis (1% v/v) and the inserted sequences were validated by Sanger Sequencing (Baseclear).

To construct psroGp2-luc, psroGp2-FMN-luc (1 µg) and the fragment sroGp2 (1 µg) were separately linearized using BlpI and BamHI in 50 µL reactions. The linearized plasmid was purified by agarose gel purification using QIAquick PCR & Gel Cleanup Kit (Qiagen) and sroGp2 with sticky ends was purified by QIAquick PCR purification kit (Qiagen). Ligation was performed by combining linearized vector (50 ng) with the insert fragment (3:1 molar ratio) in the presence of 1x DNA Ligase Buffer (2 µL) and T4 DNA Ligase (1 µL). The mixture was gently mixed, incubated at room temperature for 10 minutes and heat inactivated at 65 °C for 10 minutes. The plasmid was chilled on ice and subsequently the ligation mixture (7.5 µL) was mixed with competent TOP10F *E. coli* (50 µL).[21] The transformation, plasmid isolation and validations were performed in a similar manner as described for psroGp2-FMN-Luc.

Fragment	Sequence (5' to 3')
Luciferase	TTCTCCCGTCTCGGTTGGAAGACGCCAAAAACATAAAGAAAGGCCCGGCGCCATTCTATCCTC<br>TAGAGGATGGAACCGCTGGAGAGCAACTGCATAAGGCTATGAAGAGATACGCCCTGGTTCCT<br>GGAACAATTGCTTTTACAGATGCACATATCGAGGTGAACATCACGTACGCGGAATACTTCGAA<br>ATGTCCGTTCGGTTGGCAGAAGCTATGAAACGATATGGGCTGAATACAAATCACAGAATCGTC<br>GTATGCAGTGAAAACTCTCTTCAATTCTTTATGCCGGTGTTGGGCGCGTTATTTATCGGAGTTG<br>CAGTTGCGCCCGCGAACGACATTTATAATGAACGTGAATTGCTCAACAGTATGAACATTTCGC<br>AGCCTACCGTAGTGTTTGTTTCCAAAAAGGGGTTGCAAAAAATTTTGAACGTGCAAAAAAAAT<br>TACCAATAATCCAGAAAATTATTATCATGGATTCTAAAACGGATTACCAGGGATTTCAGTCGAT<br>GTACACGTTCGTCACATCTCATCTACCTCCCGGTTTTAATGAATACGATTTTGTACCAGAGTCCT<br>TTGATCGTGACAAAACAATTGCACTGATAATGAATTCCTCTGGATCTACTGGGTTACCTAAGGG<br>TGTGGCCCTTCCGCATAGAACTGCCTGCGTCAGATTCTCGCATGCCAGAGATCCTATTTTTGGCA<br>ATCAAATCATTCCGGATACTGCGATTTTAAGTGTTGTTCCATTCCATCACGGTTTTGGAATGTTTA<br>CTACACTCGGATATTTGATATGTGGATTTCGAGTCGTCTTAATGTATAGATTTGAAGAAGAGCTG<br>TTTTTACGATCCCTTCAGGATTACAAAATTCAAAGTGCGTTGCTAGTACCAACCCTATTTTCATTCT<br>TCGCCAAAAGCACTCTGATTGACAAATACGATTTATCTAATTTACACGAAATTGCTTCTGGGGGC<br>GCACCTCTTTCGAAAGAAGTCGGGGAAGCGGTTGCAAAACGCTTCCATCTTCCAGGGATACGAC<br>AAGGATATGGGCTCACTGAGACTACATCAGCTATTCTGATTACACCCGAGGGGGATGATAAACC<br>GGGCGCGGTCGGTAAAGTTGTTCCATTTTTTGAAGCGAAGGTTGTGGATCTGGATACCGGGAAA<br>ACGCTGGGCGTTAATCAGAGAGGCGAATTATGTGTCAGAGGACCTATGATTATGTCCGGTTATG<br>TAAACAATCCGGAAGCGACCAACGCCTTGATTGACAAGGATGGATGGCTACATTCTGGAGACAT<br>AGCTTACTGGGACGAAGACGAACACTTCTTCATAGTTGACCGCTTGAAGTCTTTAATTAAATACA<br>AAGGATATCAGGTGGCCCCCGCTGAATTGGAATCGATATTGTTACAACACCCCAACATCTTCGAC<br>GCGGGCGTGGCAGGTCTTCCCGACGATGACGCCGGTGAACTTCCCGCCGCCGTTGTTGTTTTGG<br>AGCACGGAAAGACGATGACGGAAAAAGAGATCGTGGATTACGTCGCCAGTCAAGTAACAACCG<br>CGAAAAAGTTGCGCGGAGGAGTTGTGTTTGTGGACGAAGTACCGAAAGGTCTTACCGGAAAAC<br>TCGACGCAAGAAAAATCAGAGAGATCCTCATAAAGGCCAAGAAGGGCGGAAAGTCCAAATTGT<br>AACTCGAGGGAGACGGGAGAA
sroGp2-FMN[20]	TTCTCCCGTCTCCGAGCGGCGTTGCTGCCGCTAATCATTAGCGTtatagtGAATCCgCTTAaagcttGCtt<br>attctcaGGGCGGggcgaaattCCCCACCggcggtaaatCAACTCAGttgaaagcccgcgAGCGCTTTGGGTGC<br>GaactcaaaggacaGCAGATCCggtgtaattCCGGGGCCGACggttagagtccGGATGGGAGAGAgtaacgat<br>TCTGTCGGGCATggacccgctcacGTTATTTTGGCTATATgccgccactcctaagactgccctgattCTGGTAAC<br>CATAATTTTagtgaggTTTTTTTaccATGAATCAGACGCTACTTtGGATCCaaGCAGAGTTGGGAGACG<br>CCTCA
sroGp2[20]	AACCGGTAACGTGCTGAGCGGCGTTGCTGCCGCTAATCATTAGCGTtatagtGAATCCgCTTAaagcttG<br>CttattctcaGTTTagtgaggTTTTTTTaccATGAATCAGACGCTACTTtGGATCCaaGCAGAGTTGGAAGACG

Supplementary Table S3. DNA sequences of the used fragments to construct pSroGp2-FMN-luc and pSroGp2-luc.[8,20]

In Vitro Transcription/Translation Assay

Plasmids psroGp2-FMN-luc and psroGp2-luc were used as DNA templates for the *in vitro* transcription/translation (IVTT) assay. IVTT assays were performed using the commercially available *E. coli* T7 S30 Extract System for Circular DNA (Promega). The recommended standard procedure was adapted to 11.1 µL IVTT reactions with 7.5 nM of plasmid DNA and 2 mM luciferin. IVTT reactions were performed in the presence of 1 µM FMN, 100 µM TMP or both ligands simultaneously. Nuclease-free water (1.1 µL) was used when IVTT reactions were performed in absence of any ligand. IVTT reactions (10 µL) were added to a white 384-wells plate and measured every 2 minutes after shaking for 5 seconds at 30 °C for 120 minutes on luminescence on a BioTek Synergy H1 plate reader. Peak signals (after approximately 84 minutes) were averaged, corrected for background signal, normalized over the signal for 'no ligand' and corrected for potential effect of the ligands observed in IVTT reactions using psroGp2-luc.

SYNTHESIS, PURIFICATION AND ANALYSIS OF COMPOUNDS

All reagents below were purchased from commercially available suppliers and used as received. Reactions were performed at room temperature (RT) unless otherwise stated. Reactions taking 16 hours were considered overnight reactions. Progress of reactions were determined using thin-layer chromatography using silica gel-coated plates (Merck 60 F254). The used mobile phases and corresponding R_f values are indicated per reaction. Flash column chromatography was performed with silica gel (0.040-0.063 mm, pore size 60 Å, VWR). Visualization of the spotted molecules was performed with UV light or potassium permanganate staining solution. Mass spectra were measured on a Single-Quad Thermo ISQ with UltiMate 3000 autosampler. High resolution mass spectrometry (HRMS) measurement was performed using a timsTOF (Bruker Daltonics, Bremen, Germany). NMR spectra were measured in the indicated deuterated solvents using a Bruker 400 MHz Avance III HD nanobay equipped with a Prodigy BB cryoprobe or a JEOL500 ECZ-R – Timon equipped with Royal HFC probe. Chemical shifts are displayed in parts per million (ppm) with respect to the indicated deuterated solvent or TMS (δ = 0.00 ppm) for ^{1}H and ^{13}C NMR. Coupling constants are reported in hertz (Hz) as *J* coupling constants. The multiplicity is described by the number of coupling constants, number of peaks and splitting pattern in the signal (s = singlet, d = doublet, t = triplet, q = quartet, dd = doublet of doublets, td = triplet of doublets, m = multiplet and br = broad). Peak assignments are based on ^{1}H NMR, ^{13}C NMR, ^{31}P NMR, COSY, HSQC and HMBC spectra. The spectra of the assigned peaks are depicted in the section 'SPECTRA'.

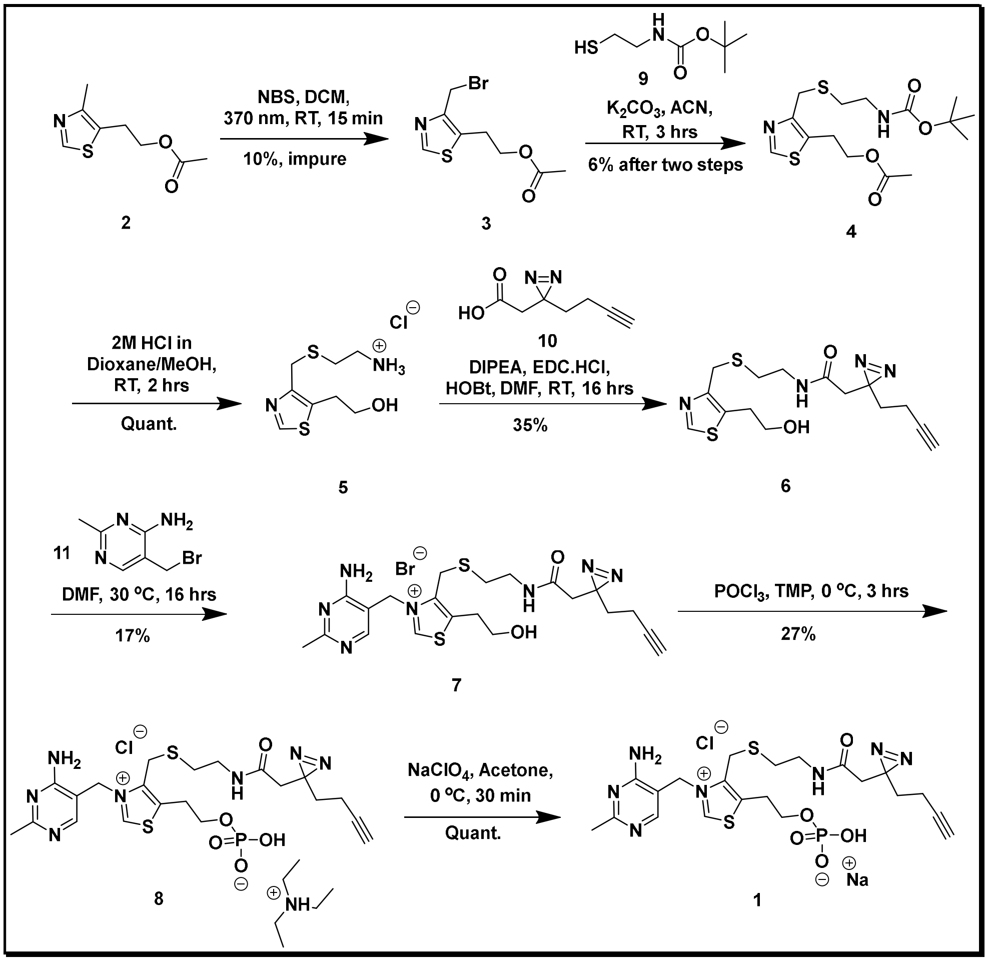

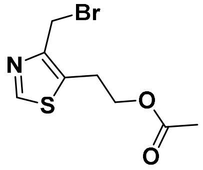

3

2-(4-(bromomethyl)thiazol-5-yl)ethyl acetate (3)

To a mixture of N-Bromosuccinimide (12 g, 68 mmol) in anhydrous dichloromethane (200 mL) was added methyl 2-(4-methyl-5-thiazolyl)ethyl acetate **2** (5.0 g, 27 mmol). The resulting

mixture was irradiated with a 370 nm UV light (Kessil PR160, max. 40 W) at 100% intensity for 15 minutes. The reaction mixture was quenched by diluting with saturated sodium thiosulfate (100 mL). The organic layer was washed with saturated sodium bicarbonate (twice) and brine (one time), dried over magnesium sulfate, filtered and concentrated *in vacuo* to yield a yellow liquid. Further purification was achieved by flash column chromatography (ethyl acetate 10% to 30% in *n*-heptane) to afford **3** as impure pale-yellow oil (0.92 g, 10% yield, 60% pure by NMR) as pale-yellow oil. **Rf** = 0.2 (30% ethyl acetate in *n*-heptane). **^{1}H NMR** (500 MHz, $CDCl_3$) δ 8.66 (s, 1H), 4.61 (s, 2H), 4.28 (t, *J* = 6.5 Hz, 2H), 3.19 (t, *J* = 6.5 Hz, 2H), 2.07 (s, 3H). **^{13}C NMR** (126 MHz, $CDCl_3$) δ 170.79, 151.29, 149.50, 132.53, 63.55, 25.99, 24.94, 20.99. **ESI-MS** $[M+H]^+$ (m/z): Calculated 265.1; Observed 265.9.

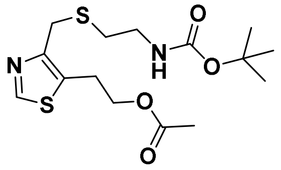

4

2-(4-(((2-((*tert*-butoxycarbonyl)amino)ethyl)thio)methyl) thiazol-5-yl)ethyl acetate (4)

To a solution of 2-(Boc-amino)ethanethiol **9** (0.70 g, 3.9 mmol) and potassium carbonate (1.0 g, 7.9 mmol) in anhydrous acetonitrile (15 mL) was added the impure solution of **3** (0.92 g) in acetonitrile (5 mL). The reaction was stirred at room temperature for three hours, diluted with ethyl acetate (200 mL) and washed twice with NaOH (1 M), aqueous saturated $NaHCO_3$ and brine. The organic layer was dried over magnesium sulfate, filtered and concentrated *in vacuo* to yield a brown liquid. Further purification was achieved by flash column chromatography (ethyl acetate 20-50% in *n*-heptane over 20 column volumes) to afford **4** as pale-yellow oil (0.55 g, 6% after two steps). **Rf** = 0.2 (40% ethyl acetate in *n*-heptane). **^{1}H NMR** (500 MHz, $CDCl_3$) δ 8.62 (s, 1H), 5.09 (s, 1H), 4.26 (t, *J* = 6.6 Hz, 2H), 3.86 (s, 2H), 3.32 (q, *J* = 6.2 Hz, 2H), 3.16 (t, *J* = 6.6 Hz, 2H), 2.68 (t, *J* = 6.4 Hz, 2H), 2.06 (s, 3H), 1.43 (s, 9H). **^{13}C NMR** (126 MHz, $CDCl_3$) δ 170.89, 155.94, 150.87, 150.61, 129.72, 79.43, 64.09, 39.80, 32.47, 29.10, 28.52, 25.88, 20.85. **ESI-MS** $[M+H]^+$ (m/z): calculated 361.4; Observed 361.1.

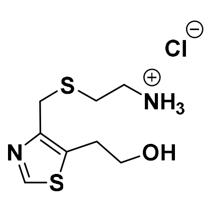

5

2-(((5-(2-hydroxyethyl)thiazol-4-yl)methyl)thio)ethan-1-aminium chloride (5)

To a solution of 2 M HCl in methanol/1,4-dioxane (1/1 (v/v)) was added **4** (0.16 g, 0.44 mmol). The reaction mixture was left stirring at room temperature for two hours. The reaction mixture was concentrated *in vacuo* to afford **5** (97 mg, quant.) as orange oil. **Rf** = 0 (ethyl acetate: *n*-heptane). **^{1}H NMR** (500 MHz, DMSO-D_6) δ 8.95 (s, 1H), 8.13 (s, 3H), 3.93 (s, 2H), 3.59 (t, *J* = 6.3 Hz, 2H), 3.01 – 2.95 (m, 4H), 2.77 (t, *J* = 7.2 Hz, 2H). **^{13}C NMR** (126 MHz, DMSO-D_6) δ 152.30, 149.21, 132.43, 61.79, 38.83, 29.72, 29.01, 28.54. **ESI-MS** $[M+H]^+$ (m/z): calculated 219.3; Observed 219.1.

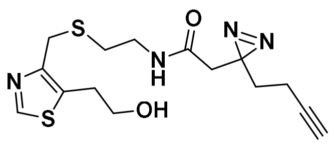

6

2-(3-(but-3-yn-1-yl)-3H-diazirin-3-yl)-*N*-(2-(((5-(2-hydroxyethyl)thiazol-4-yl)methyl)thio)ethyl)acetamide (6)

To a solution of **10** (see below, 81 mg, 0.53 mmol) in anhydrous dimethylformamide (4 mL) were added 1-hydroxybenzotriazole hydrate (89 mg, 0.66 mmol), 1-ethyl-3-(3-dimethylaminopropyl)carbodiimide hydrochloric acid (EDC.HCl) (0.13 mg, 0.66 mmol) and diisopropylethylamine (0.15 mL, 0.88 mmol). The reaction mixture was stirred for 5 minutes. Compound **5** (97 mg, 0.44 mmol) was dissolved in dimethylformamide (1 mL) and added dropwise to the reaction mixture. The reaction was left stirring at room temperature for 16 hours. The reaction mixture was diluted with ethyl acetate and extracted with saturated sodium bicarbonate solution. The aqueous layer was washed with ethyl acetate (three times). The organic layers were combined and washed with brine (three times), dried over magnesium sulfate, filtered and concentrated *in vacuo* to obtain an orange oil. Further purification was achieved using flash column chromatography (80%-100% ethyl acetate in *n*-heptane) to obtain **6** as orange oil (54 mg, 35%). **Rf** = 0.2 (90% EtOAc in *n*-heptane). **^{1}H NMR** (500 MHz, $CDCl_3$) δ 8.65 (s, 1H), 6.83 (s, 1H), 3.90 (s, 2H), 3.87 (t, *J* = 6.2 Hz, 2H), 3.48 (q, *J* = 5.9 Hz, 2H), 3.08 (t, *J* = 6.1 Hz, 2H), 2.71 (t, *J* = 6.2 Hz, 2H), 2.25 (s, 2H), 2.05 (td, *J* = 7.3, 2.5 Hz, 2H), 2.01 (t, *J* = 2.6 Hz, 1H), 1.77 (t, *J* = 7.3 Hz, 2H). **^{13}C NMR** (126 MHz, $CDCl_3$) δ

168.08, 151.02, 150.39, 131.31, 82.83, 69.64, 63.14, 41.57, 39.34, 32.12, 31.86, 29.81, 29.11, 26.22, 13.40. **ESI-MS** $[M+H]^+$ (m/z): calculated 353.5; Observed 353.1.

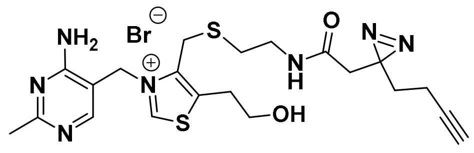

7

3-((4-amino-2-methylpyrimidin-5-yl)methyl)-4-(((2-(2-(3-(but-3-yn-1-yl)-3H-diazirin-3-yl)acetamido)ethyl)thio)methyl)-5-(2-hydroxyethyl)thiazol-3-ium (7)

To a solution of **6** (77 mg, 0.21 mmol) in anhydrous dimethylformamide (0.50 mL) was added 4-amino-5-bromomethyl-2-methylpyrimidine hydrobromide **11** (60 mg, 0.29 mmol). The reaction was left stirring at 35 °C overnight. The reaction was concentrated *in vacuo*, dissolved in MilliQ-graded water (3.0 mL), filtered and further purified by preparatory reverse-phase high-performance liquid chromatography (Shimadzu, 5-95% acetonitrile in water containing 100 mM triethylamine bicarbonate buffer (TEAB), 45 minutes, 5 mL/min) to obtain **7** (20 mg, 17%) as white solid. **^{1}H NMR** (500 MHz, DMSO-D_6) δ 9.53 (s, 1H), 8.22 (t, J = 5.8 Hz, 1H), 8.11 (s, 1H), 7.22 (s, 2H), 5.36 (s, 2H), 3.76 (t, 2H), 3.25 – 3.12 (m, 2H), 2.82 (t, J = 2.7 Hz, 1H), 2.58 (s, 3H), 2.39 (s, 3H), 2.33 – 2.23 (m, 4H), 2.23 (s, 2H), 2.03 (td, J = 7.5, 2.6 Hz, 6H), 1.65 (t, J = 7.5 Hz, 4H). **^{13}C NMR** (126 MHz, DMSO-D_6) δ 168.96, 168.05, 162.55, 159.04, 156.40, 143.23, 140.08, 103.86, 83.66, 72.36, 64.05, 51.81, 40.05, 38.76, 32.35, 27.15, 25.97, 25.88, 25.74, 13.19, 12.42. **ESI-MS** $[M]^+$ (m/z): calculated 474.6; Observed 474.1.

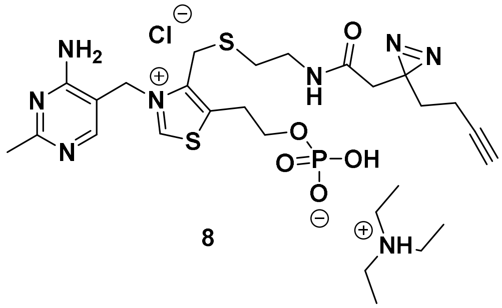

8

2-(3-((4-amino-2-methylpyrimidin-5-yl)methyl)-4-(((2-(2-(3-(but-3-yn-1-yl)-3H-diazirin-3-yl)acetamido)ethyl)thio)methyl)thiazol-3-ium-5-yl)ethyl hydrogen phosphate triethylammonium chloride (8)

A solution of **7** (15 mg, 0.025 mmol) in anhydrous trimethyl phosphate (TMP) was brought under nitrogen atmosphere. The solution was cooled to 0 °C and phosphoryl chloride (25 µL, 0.27 mmol) was added dropwise. The reaction mixture was stirred on ice for 4 hours. Subsequently, the reaction was quenched using 5.0 mL of 0.5 M TEAB and neutralized with triethylamine (TEA). The crude mixture was purified by preparatory reverse-phase high-performance liquid chromatography (Shimadzu, 5-95% acetonitrile in 100 mM TEAB, 45 minutes, 5 mL/min). Product **8** was obtained as white triethylammonium salt (5.0 mg, 27%). **^{1}H NMR** (500 MHz, DMSO-D_6) δ 9.52 (s, 1H), 8.16 (s, 1H), 7.21 (s, 2H), 5.50 (s, 2H), 4.28 (s, 2H), 3.91 (q, J = 7.0 Hz, 2H), 3.29 – 3.26 (m, 2H), 3.15 (q, J = 6.6 Hz, 2H), 2.80 (t, J = 2.7 Hz, 1H), 2.65 – 2.58 (m, 2H), 2.38 (s, 3H), 2.24 (s, 2H), 2.01 (td, J = 7.5, 3.0 Hz, 5H), 1.64 (t, 3H). **^{31}P NMR** (500 MHz, DMSO-D_6) δ 0.03.

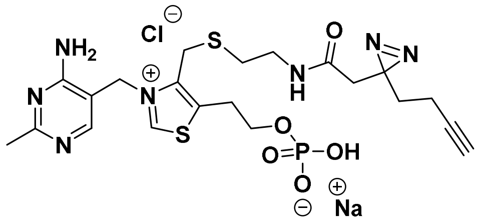

1

2-(3-((4-amino-2-methylpyrimidin-5-yl)methyl)-4-(((2-(2-(3-(but-3-yn-1-yl)-3H-diazirin-3-yl)acetamido)ethyl)thio)methyl)thiazol-3-ium-5-yl)ethyl hydrogen phosphate sodium chloride (1)

Compound **8** (0.8 mg, 0.001 mmol) was converted to the sodium salt by ion-exchange with sodium perchlorate in acetone. A solution of sodium perchlorate in acetone (1 mL, 25 mg/mL) was added to TEA salt **8** and the mixture was vortexed briefly and stored on ice for 5 minutes. The suspension was centrifuged (17,000 g, 5 minutes) at 4 °C. The supernatant was discarded, and this procedure was performed twice more. Subsequently, the precipitate was washed with acetone thrice. The sodium salt **1** was obtained as white powder (0.6 mg, quant.). **^{1}H NMR** (500 MHz, D_2O, 4 °C) δ 7.97 (s, 1H), 5.41 (s, 2H), 4.02 (s, 2H), 3.77 (q, J = 5.4 Hz, 2H), 3.20 (t, J = 6.3 Hz, 2H), 3.08 (t, J = 5.3 Hz, 2H), 2.57 (t, J = 5.1 Hz, 2H), 2.26 (s, 3H), 2.21 (s, 2H), 2.09 (t, J = 2.3 Hz, 1H), 1.80 (td, J = 8.4, 4.2 Hz, 2H), 1.50 (t, J = 7.2 Hz, 2H). **^{31}P NMR** (500 MHz, D_2O) δ -0.78. **HRMS** $[M]^+$ (m/z): calculated 554.1404; Observed 554.1385.

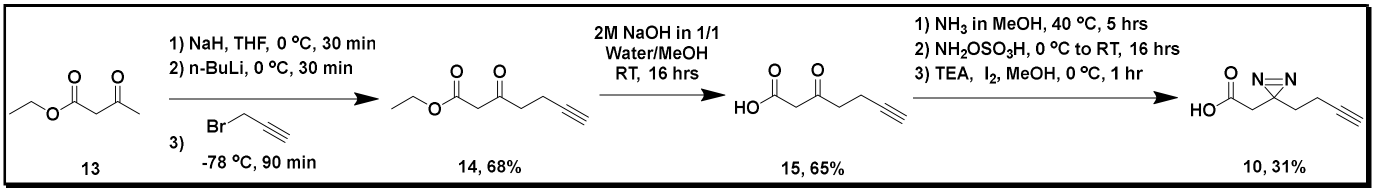

The synthesis of photoaffinity linker **10** was based on Chao *et al.* (2020)[22].

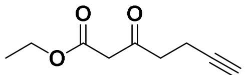
14

Ethyl 3-oxohept-6-ynoate (14)

A mixture of sodium hydride (3.2 g, 60% suspension in mineral oil, 79 mmol) in dry THF (25 mL) was brought to 0 °C on ice. Ethyl acetoacetate **13** (5.1 mL, 38 mmol) was added to this mixture. The reaction mixture was stirred at 0 °C for 30 minutes. *n*-Butyllithium (36 mL, 58 mmol) was added dropwise to the reaction mixture. The heterogeneous mixture was stirred on ice for 30 minutes. The reaction mixture was brought to -78 °C using a dry ice-acetone bath. Propargyl bromide (5.0 mL of 80% (v/v) in toluene, 58 mmol) was slowly added to the reaction mixture. The reaction was left stirring for three hours. The reaction mixture was slowly quenched using water (75 mL). The mixture was extracted using diethyl ether (six times), washed with brine (three times), dried over magnesium sulfate, filtered, and concentrated *in vacuo* to yield an orange mixture. Further purification was achieved by silica column chromatography (*n*-heptane: ethyl acetate 20% over 10 column volumes) to afford **14** (4.4 g, 68%) as an orange oil. $\mathbf{R_f}$ = 0.3 (25% ethyl acetate in *n*-heptane). ^{1}**H NMR** (500 MHz, $CDCl_3$) δ 4.18 (q, *J* = 7.1 Hz, 2H), 3.44 (s, 2H), 2.79 (t, *J* = 6.6 Hz, 2H), 2.45 (td, 2H), 1.94 (t, *J* = 2.1 Hz, 1H), 1.26 (t, *J* = 1.2 Hz, 3H). ^{13}**C NMR** (126 MHz, $CDCl_3$) δ 200.67, 167.01, 82.62, 69.09, 69.05, 61.59, 49.30, 41.69, 14.13, 12.91. **ESI-MS** $[M+H]^+$ (m/z): calculated 169.2, observed: 169.1.

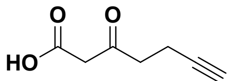
15

3-oxohept-6-ynoic acid (15)

To a solution of 2 M sodium hydroxide in water/methanol (1/1 (v/v)) was added **14** (5.0 g, 30 mmol) and the reaction was stirred at room temperature overnight. The reaction mixture was concentrated *in vacuo* and diluted using sodium hydroxide (1 M). The mixture was extracted with ethyl acetate (three times). The aqueous layer was acidified using hydrochloric acid (1 M) and extracted with ethyl acetate (five times). The organic layers were combined, washed with saturated brine (three times), dried over magnesium sulfate, filtered, and concentrated *in vacuo* to yield compound **15** (3.6 g, 86%) as dark yellow oil. ^{1}**H NMR** (500 MHz, $CDCl_3$) δ 11.83 (s, 1H), 3.55 (s, 2H), 2.84 (t, *J* = 6.6 Hz, 2H), 2.50 (td, *J* = 7.2, 2.6 Hz, 3H), 1.98 (t, *J* = 2.7 Hz, 1H). ^{13}**C NMR** (126 MHz, $CDCl_3$) δ 201.73, 170.63, 82.17, 69.38, 48.00, 41.86, 12.92. **ESI-MS** $[M+H]^+$ (m/z): calculated 163.1, observed: 163.2.

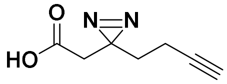
10

2-(3-(but-3-yn-1-yl)-3H-diazirin-3-yl)acetic acid (10)

Compound **15** (0.15 g, 1.1 mmol) was dissolved in anhydrous methanol (0.5 mL) and brought to 0 °C under argon atmosphere. Saturated ammonia in methanol (7 M, 1.5 mL, 11 mmol) was added to the mixture and the reaction mixture was stirred for 3h at 0 °C. Hydroxylamine-O-Sulfonic Acid (0.15 g, 1.3 mmol) was dissolved in dry methanol (1 mL) and slowly added to the reaction mixture at 0 °C. The reaction was allowed to reach room temperature overnight. The reaction mixture was concentrated *in vacuo*. The residue was rinsed with anhydrous methanol (three times) and filtered. The filtrate was concentrated *in vacuo* and redissolved in anhydrous methanol (2 mL). Triethylamine (0.30 ml, 2.1 mmol) was added dropwise at 0 °C and the mixture was stirred for 15 minutes. Iodine (0.22 g, 1.1 mmol) was added in portions to the solution at 0 °C until no color change was observed for 30 minutes. The reaction was diluted with ethyl acetate and washed with 1 M hydrogen chloride, saturated sodium thiosulfate and saturated brine. The organic layer was washed with 1 M sodium hydroxide (three times). The combined aqueous layers were washed with ethyl acetate (three times) and acidified using 1 M hydrogen chloride. Extraction with ethyl acetate (five times) afforded compound **10** (50 mg, 31% yield) as red oil. ^{1}**H NMR** (500 MHz, $CDCl_3$) δ 2.40 (s, 2H), 2.07 (td, *J* = 7.3, 2.6 Hz, 2H), 2.01 (t, *J* = 2.6 Hz, 1H),

1.80 (t, *J* = 7.3 Hz, 2H). **^{13}C NMR** (126 MHz, $CDCl_3$) δ 175.23, 82.56, 69.65, 39.59, 32.04, 25.36, 13.33. **ESI-MS** $[M+H_2O]^+$ (m/z): calculated 170.2, observed: 170.2.

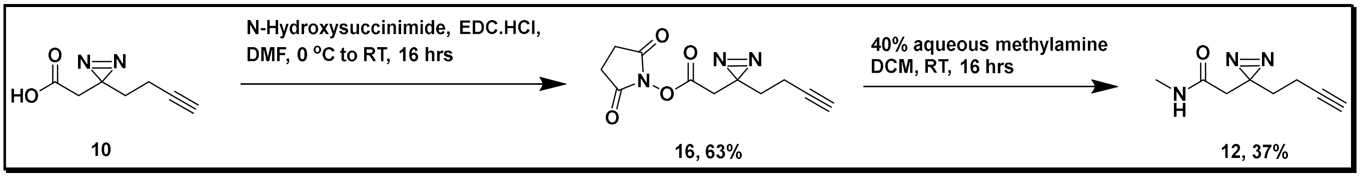

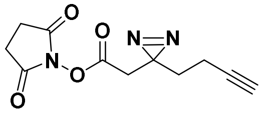

16

2,5-Dioxopyrrolidin-1-yl 2-(3-(but-3-yn-1-yl)-3H-diazirin-3-yl)acetate (16)

To a solution of **10** (80 mg, 0.53 mmol) in anhydrous *N, N*-dimethylformamide (3.0 mL) was added *N*-hydroxysuccinimide (67 mg, 0.58 mmol) in one portion. The mixture was brought to 0 °C under argon atmosphere. (*N*-(3-dimethylaminopropyl)-*N'*-ethylcarbodiimide hydrochloride (0.12 g, 0.63 mmol) was added in portions. The reaction mixture was allowed to reach room temperature overnight. The reaction mixture was diluted with ethyl acetate and washed with water (three times), 1 M HCl (three times) and brine (one time). The organic layer was dried over magnesium sulfate and concentrated *in vacuo*. Further purification was achieved by flash column chromatography (1/1 ethyl acetate in *n*-heptane) to afford **16** (82 mg, 63%) as pale-yellow oil. **R_f** = 0.2 (50% ethyl acetate in *n*-heptane). **^{1}H NMR** (500 MHz, $CDCl_3$) δ 2.87 – 2.85 (m, 4H), 2.65 (s, 2H), 2.10 (td, *J* = 7.3, 2.7 Hz, 2H), 2.04 (t, *J* = 5.2 Hz, 1H), 1.84 (t, *J* = 7.2 Hz, 2H). **^{13}C NMR** (126 MHz, $CDCl_3$) δ 168.79, 164.71, 82.13, 69.99, 37.04, 31.60, 25.72, 25.11, 13.33. **ESI-MS** $[M+H]^+$ (m/z): calculated 272.2, observed: 272.1.

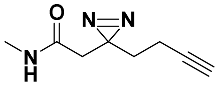

12

2-(3-(But-3-yn-1-yl)-3H-diazirin-3-yl)-*N*-methylacetamide (12)

A solution of **16** (50 mg, 0.20 mmol) in anhydrous dichloromethane (0.50 mL) was slowly added to a solution of 40% aqueous methylamine (26 µL, 0.30 mmol) in water (2 mL). The resulting reaction mixture was stirred at room temperature overnight. The mixture was concentrated *in vacuo* and further purified by flash column chromatography (0-5% methanol in dichloromethane) to yield **12** (12 mg, 37%) as white powder. **R_f** = 0.2 (2.5% methanol in dichloromethane). **^{1}H NMR** (400 MHz, $CDCl_3$) δ 5.85 (s, 1H), 2.83 (d, *J* = 4.8 Hz, 3H), 2.24 (s, 2H), 2.05 (td, *J* = 7.2, 2.3 Hz, 2H), 2.01 (t, *J* = 2.6 Hz, 1H), 1.77 (t, *J* = 7.2 Hz, 2H). **^{13}C NMR** (101 MHz, $CDCl_3$) δ 168.36, 82.77, 69.60, 41.63, 32.05, 26.58, 26.09, 13.37. **ESI-MS** $[M+H]^+$ (m/z): calculated 166.2, observed: 166.2.

SPECTRA

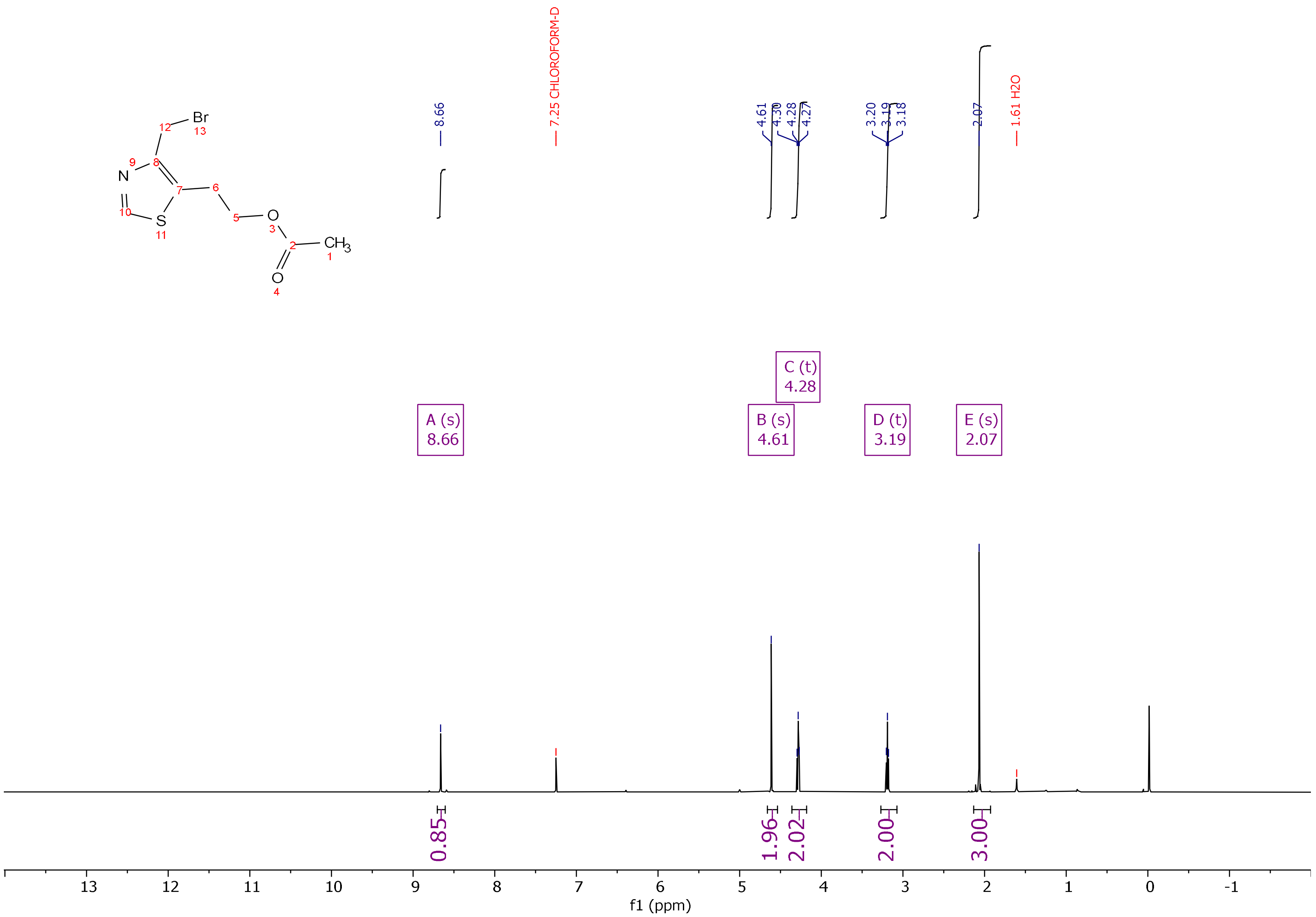

Br
N
S
O
CH3
O
8.66
7.25 CHLOROFORM-D
4.61
4.30
4.28
4.27
3.20
3.19
3.18
2.07
1.61 H2O
A (s)
8.66
B (s)
4.61
C (t)
4.28
D (t)
3.19
E (s)
2.07
0.85
1.96
2.02
2.00
3.00
f1 (ppm)

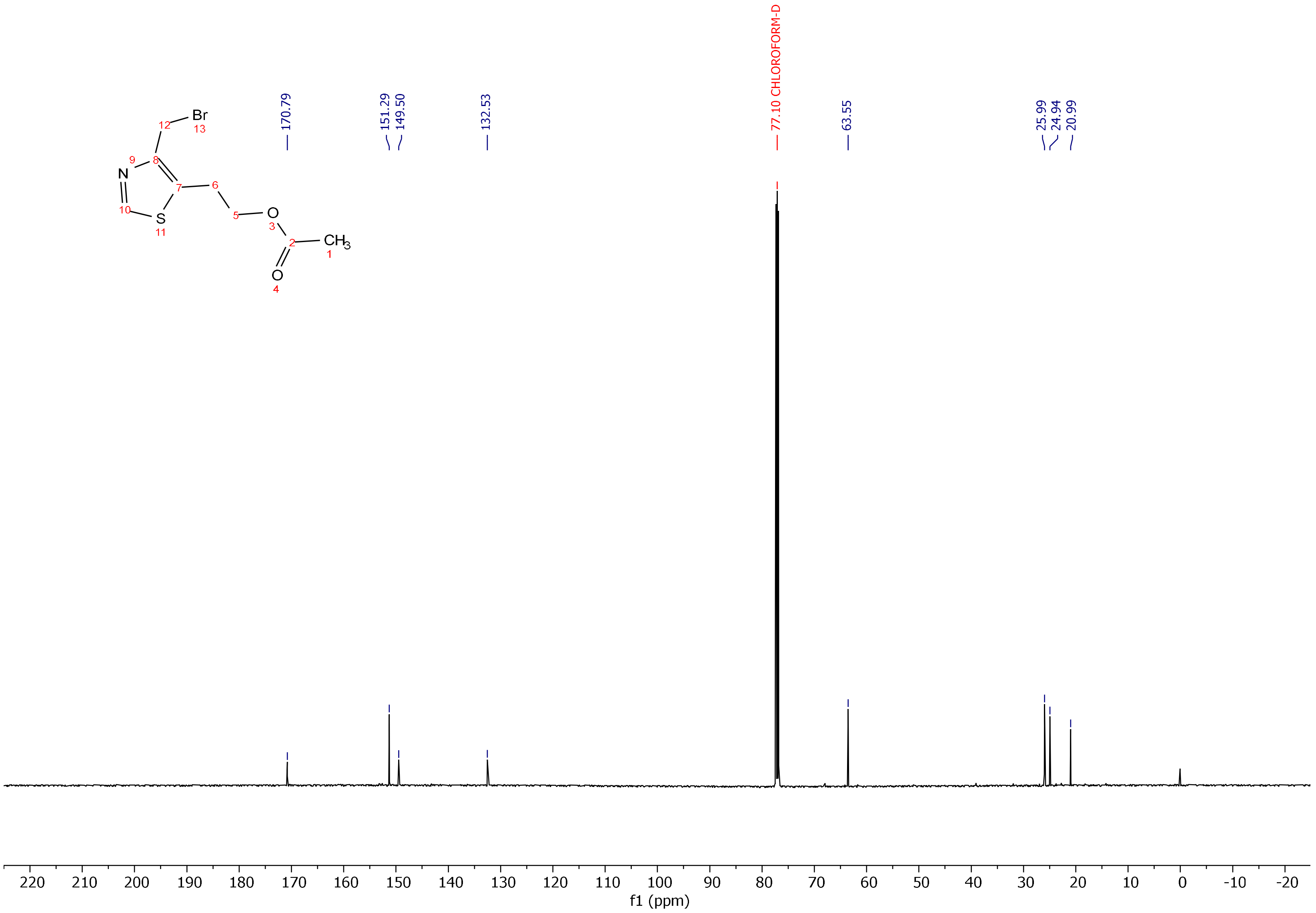

Br
N
S
CH3
O
O
170.79
151.29
149.50
132.53
77.10 CHLOROFORM-D
63.55
25.99
24.94
20.99
220
210
200
190
180
170
160
150
140
130
120
110
100
90
80
70
60
50
40
30
20
10
0
-10
-20
f1 (ppm)

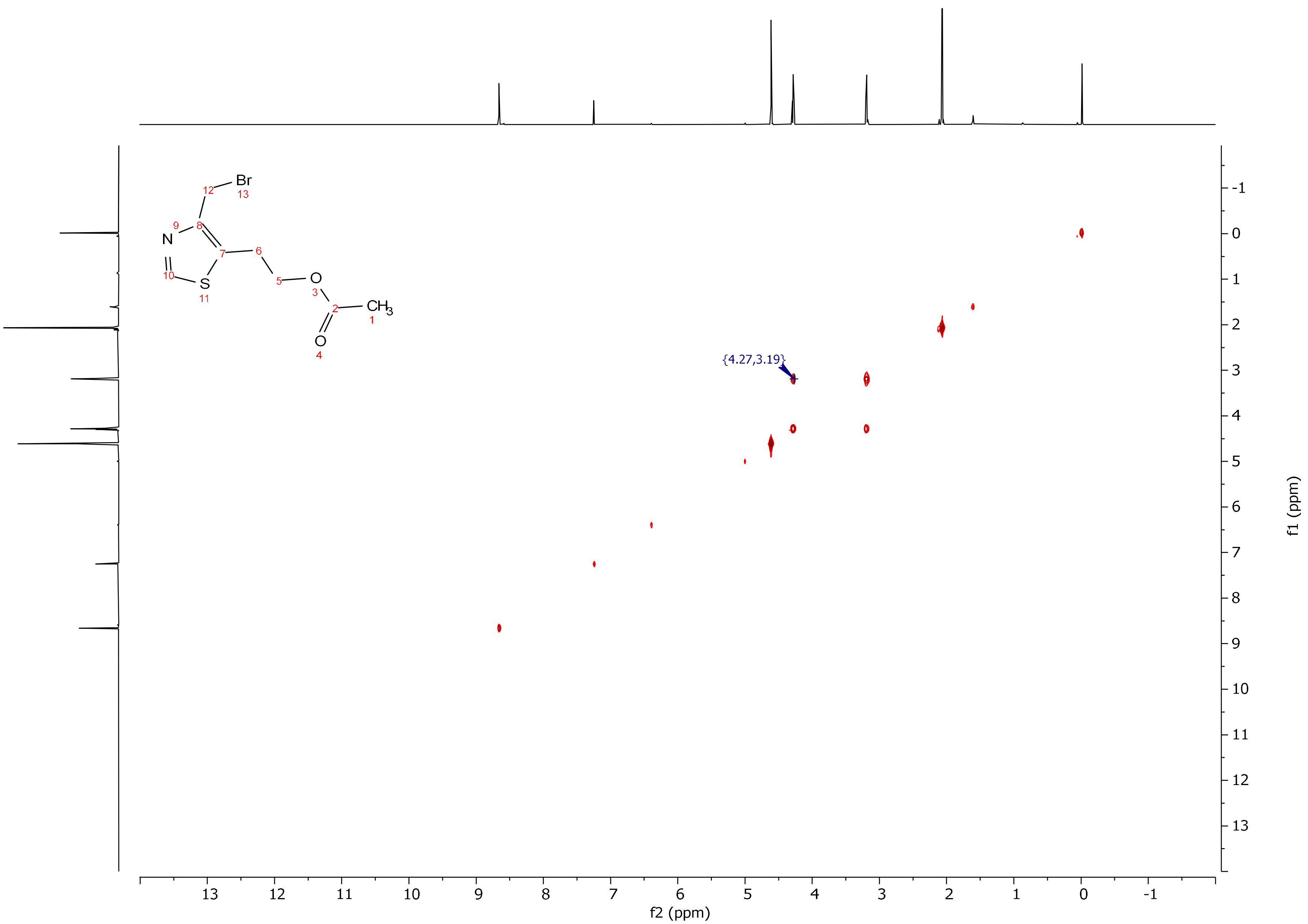

Br
N
S
O
CH3
O
{4.27,3.19}
f1 (ppm)
f2 (ppm)

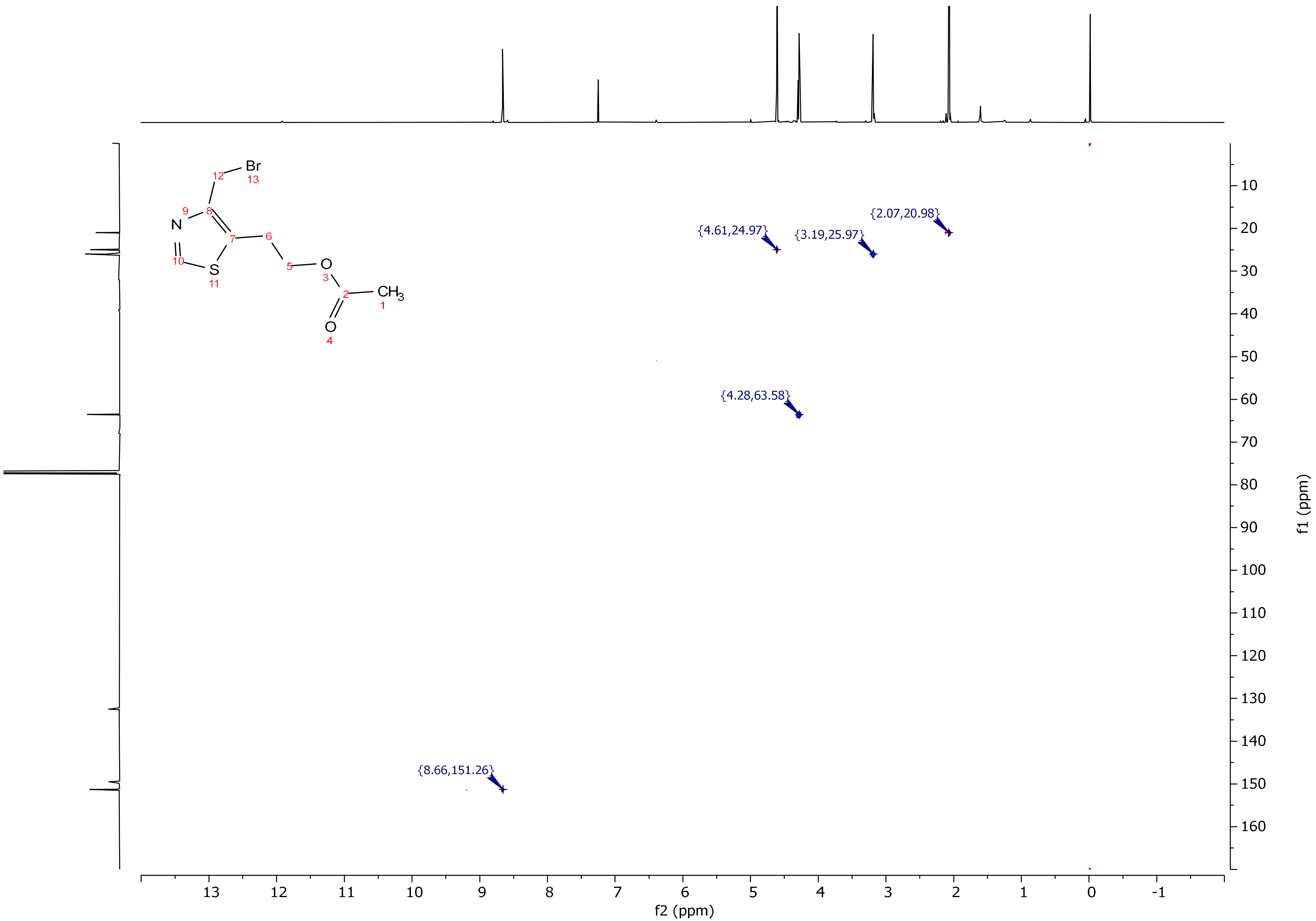
Br
12
13
9
8
N
7
6
10
5
O
S
3
11
2
CH3
1
O
4
{4.61,24.97}
{3.19,25.97}
{2.07,20.98}
{4.28,63.58}
{8.66,151.26}
f2 (ppm)
f1 (ppm)

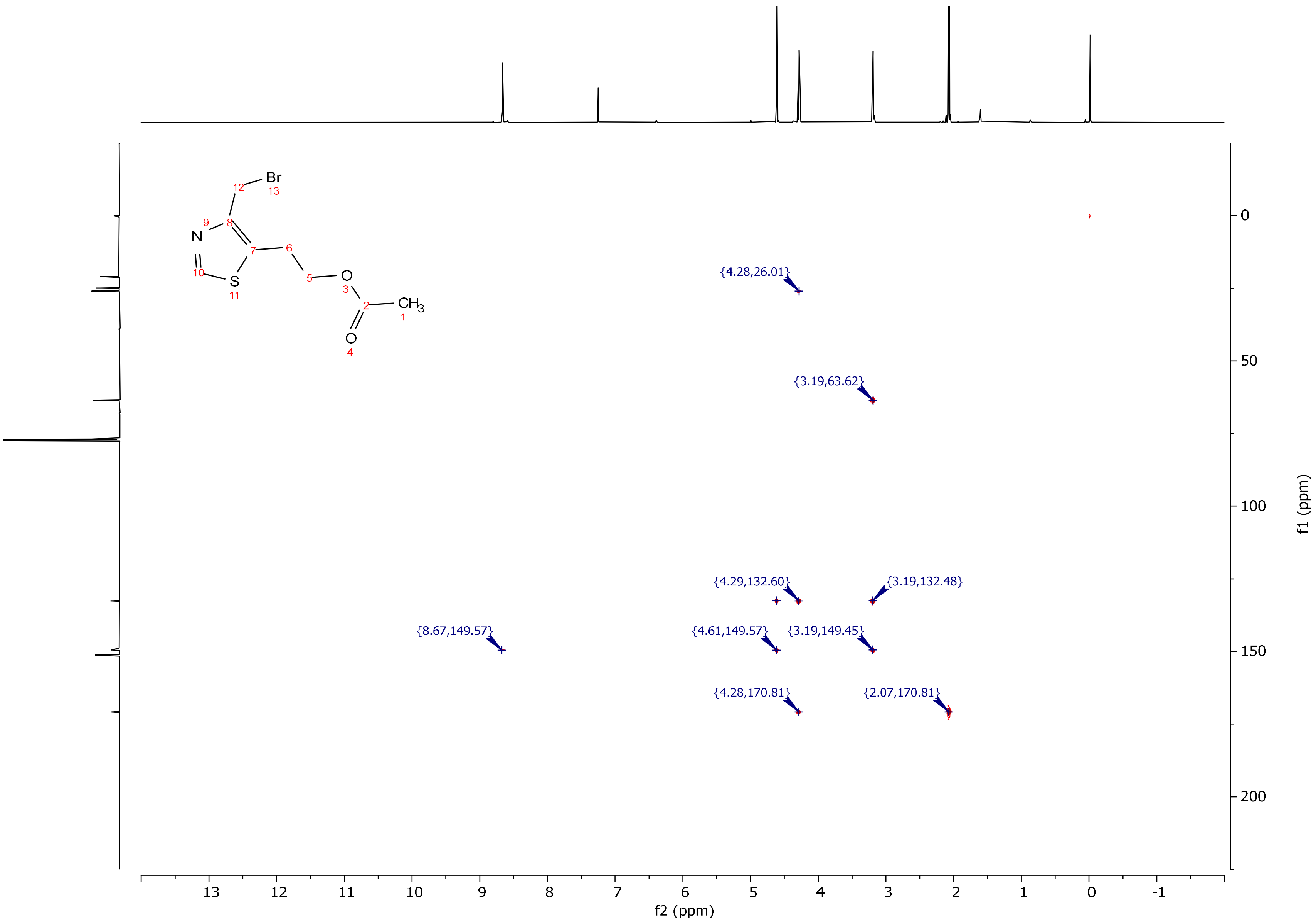
Br
12
13
9
8
N
7
6
10
S
5
O
11
3
2
CH3
1
O
4
{4.28,26.01}
{3.19,63.62}
{4.29,132.60}
{3.19,132.48}
{8.67,149.57}
{4.61,149.57}
{3.19,149.45}
{4.28,170.81}
{2.07,170.81}
f2 (ppm)
f1 (ppm)

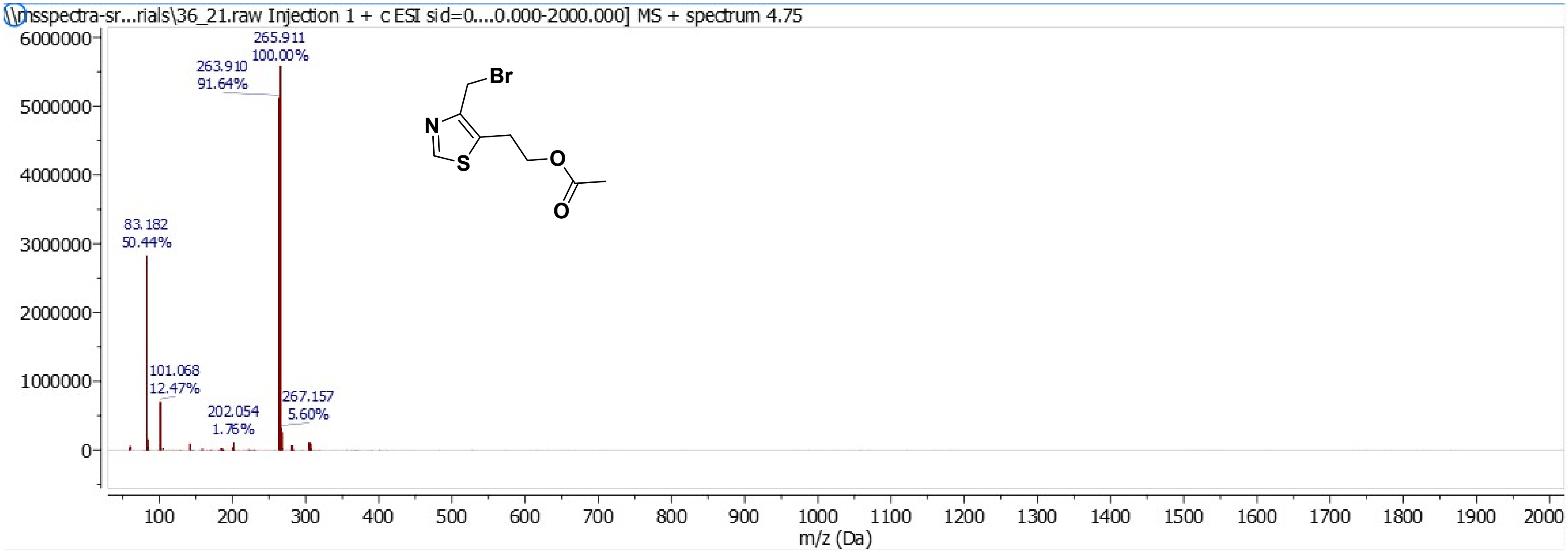
\\msspectra-sr...rials\36_21.raw Injection 1 + c ESI sid=0....0.000-2000.000] MS + spectrum 4.75
265.911
100.00%
263.910
91.64%
83.182
50.44%
101.068
12.47%
202.054
1.76%
267.157
5.60%
Br
N
S
O
O
m/z (Da)

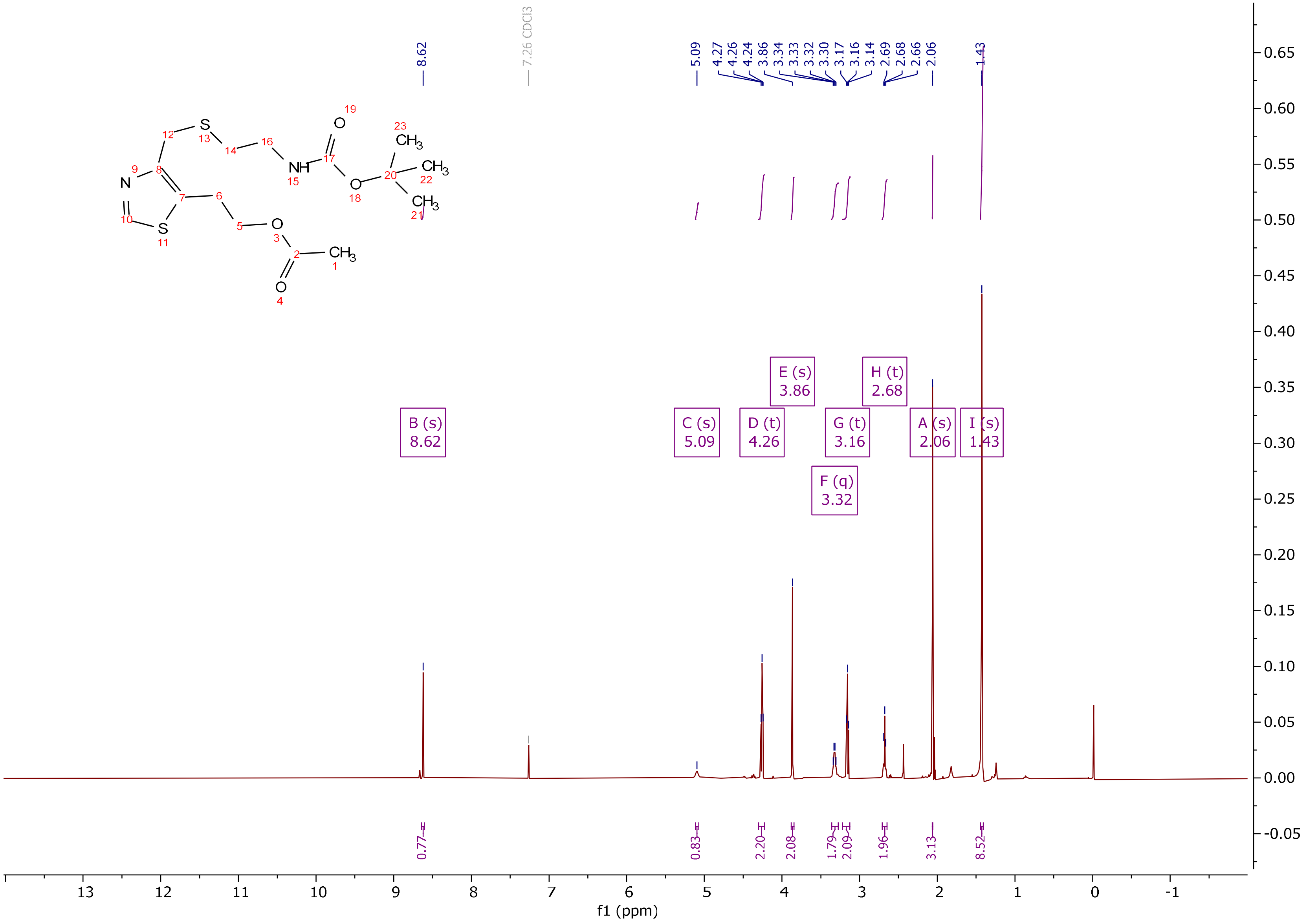

8.62
7.26 CDCl3
5.09
4.27
4.26
4.24
3.86
3.34
3.33
3.32
3.30
3.17
3.16
3.14
2.69
2.68
2.66
2.06
1.43
B (s)
8.62
C (s)
5.09
D (t)
4.26
E (s)
3.86
F (q)
3.32
G (t)
3.16
H (t)
2.68
A (s)
2.06
I (s)
1.43
0.77
0.83
2.20
2.08
1.79
2.09
1.96
3.13
8.52
f1 (ppm)

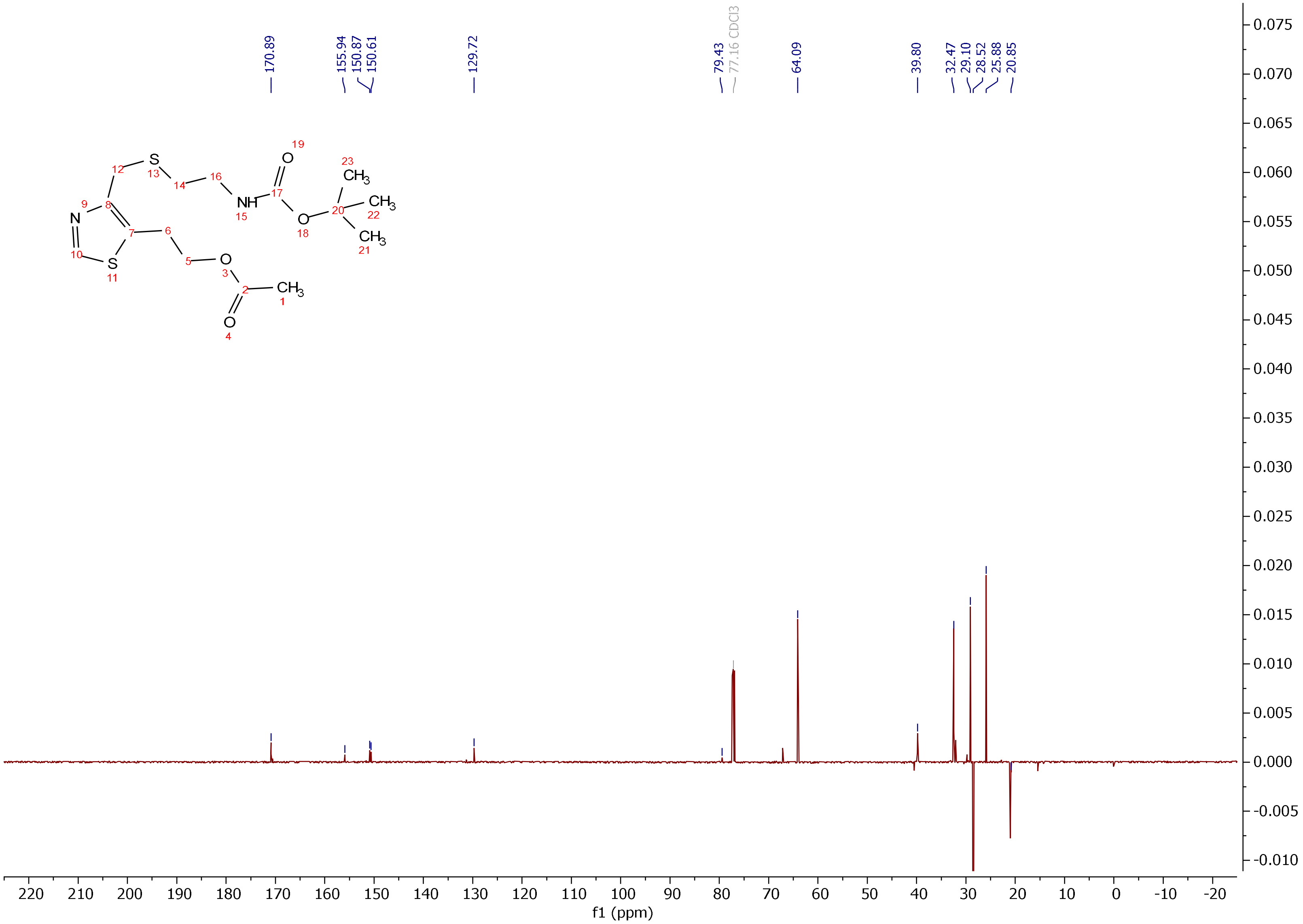
170.89
155.94
150.87
150.61
129.72
79.43
77.16 CDCl3
64.09
39.80
32.47
29.10
28.52
25.88
20.85
f1 (ppm)

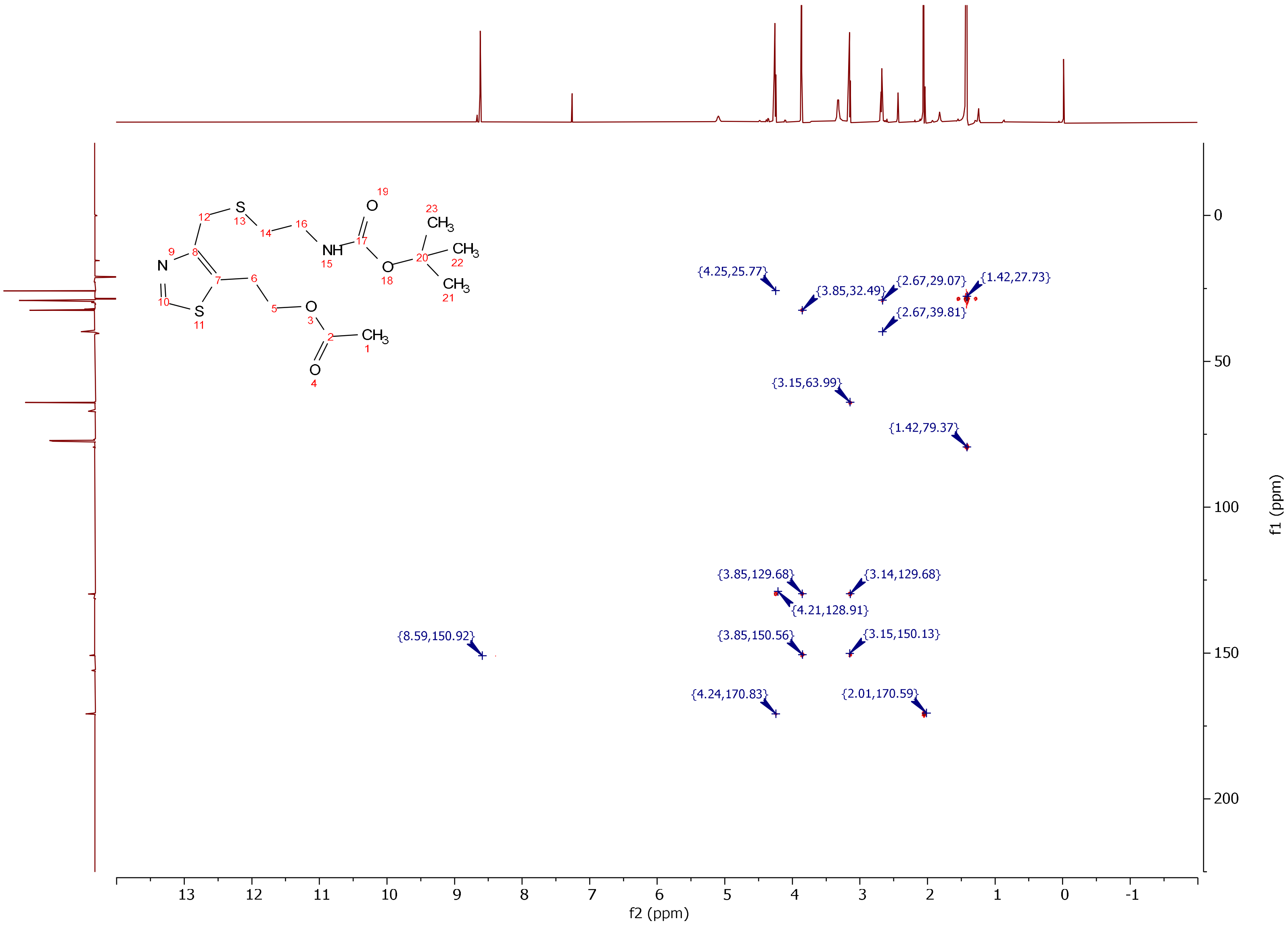

{4.25,25.77}
{3.85,32.49}
{2.67,29.07}
{1.42,27.73}
{2.67,39.81}
{3.15,63.99}
{1.42,79.37}
{3.85,129.68}
{3.14,129.68}
{4.21,128.91}
{8.59,150.92}
{3.85,150.56}
{3.15,150.13}
{4.24,170.83}
{2.01,170.59}
f2 (ppm)
f1 (ppm)

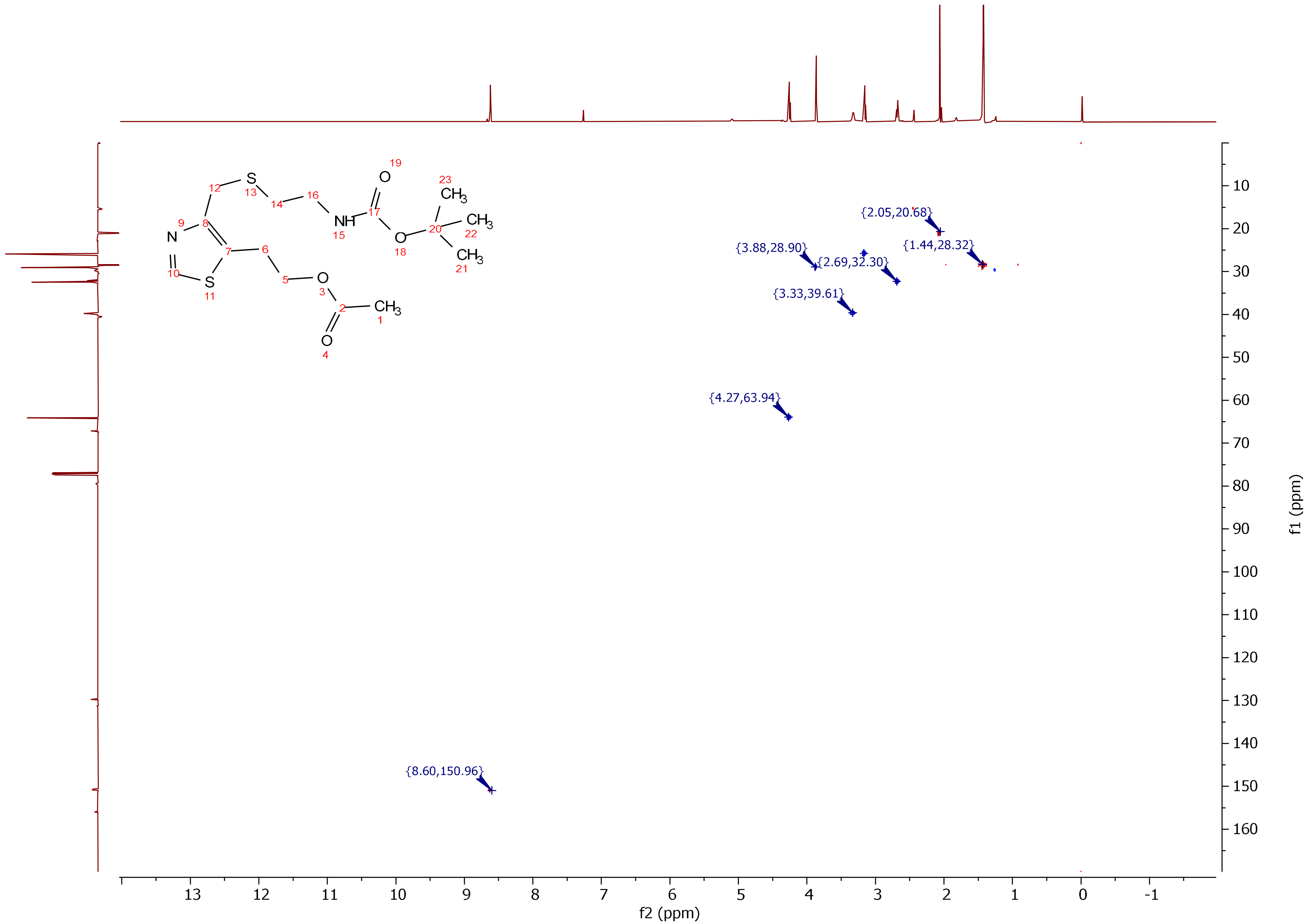
{2.05,20.68}
{3.88,28.90}
{2.69,32.30}
{1.44,28.32}
{3.33,39.61}
{4.27,63.94}
{8.60,150.96}
f2 (ppm)
f1 (ppm)

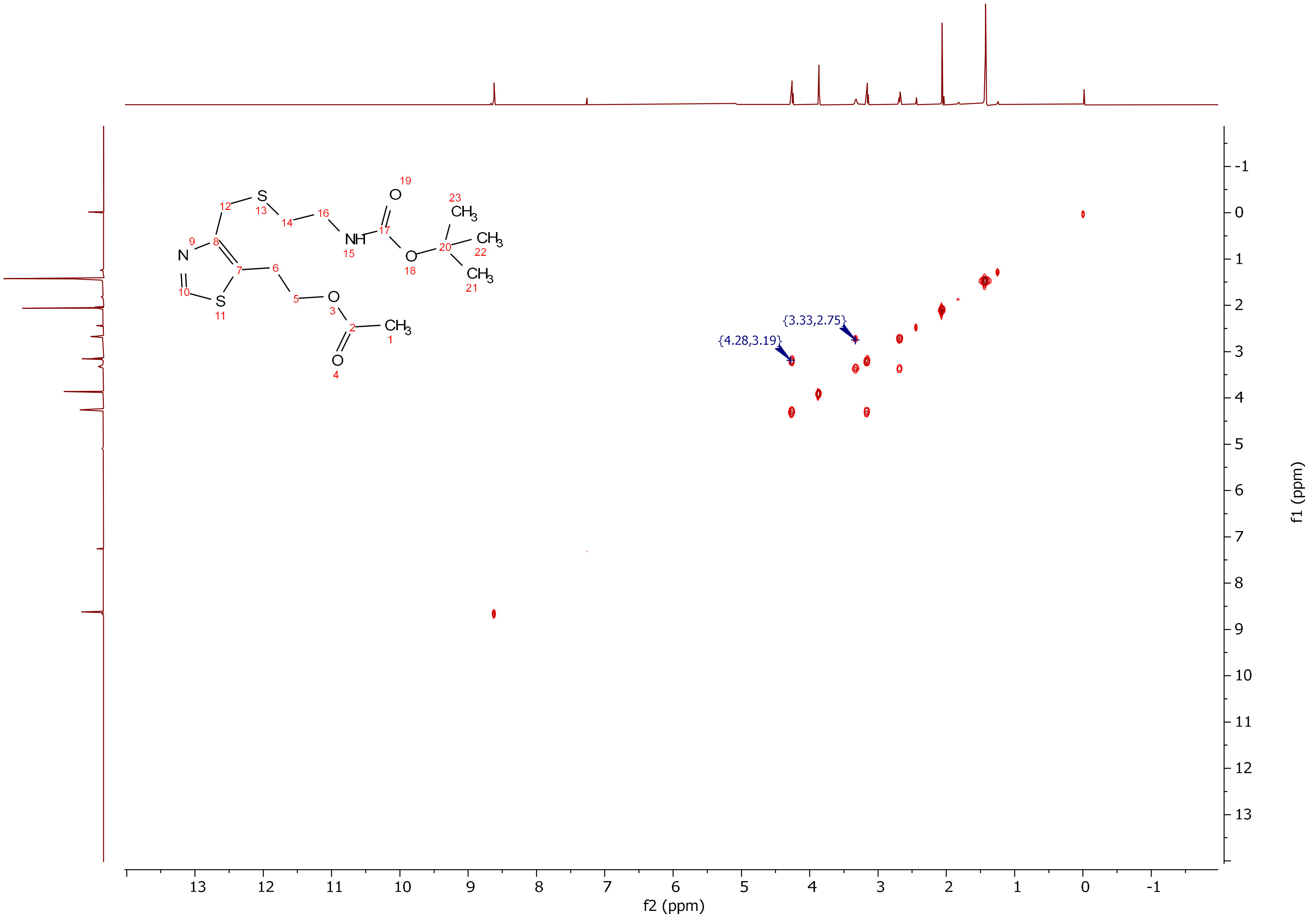
{3.33,2.75}
{4.28,3.19}
f2 (ppm)
f1 (ppm)

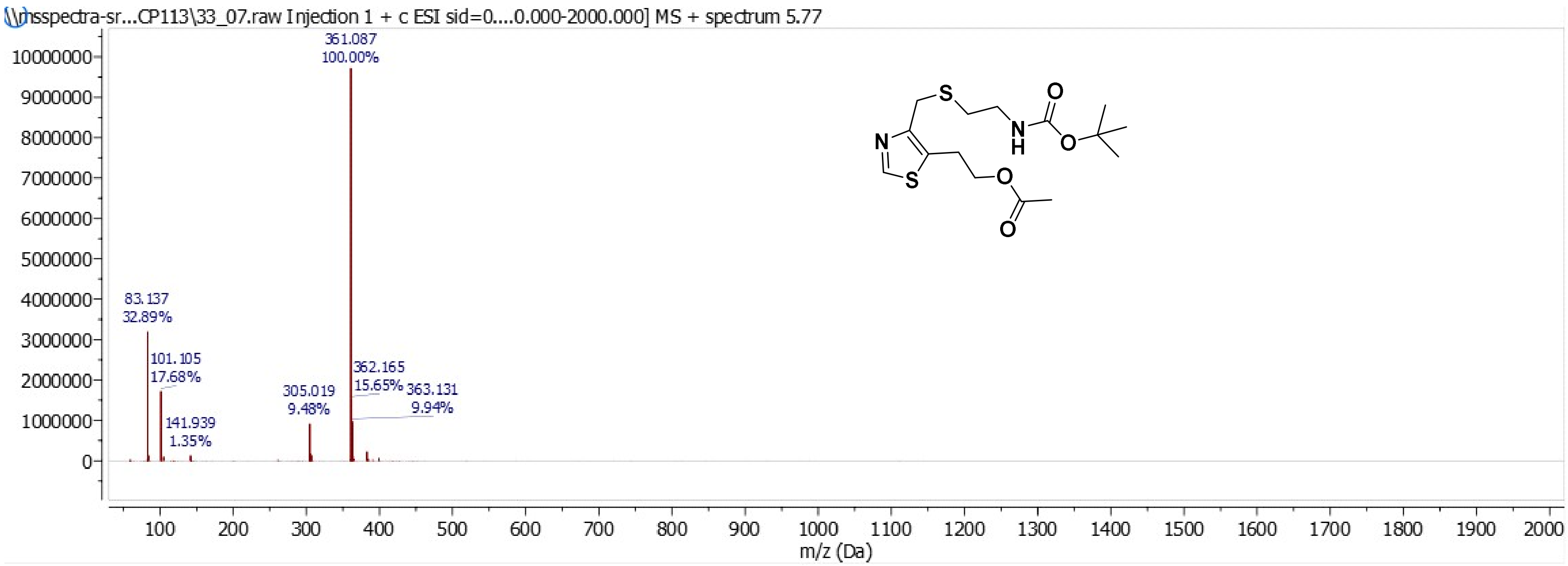
\\msspectra-sr...CP113\33_07.raw Injection 1 + c ESI sid=0....0.000-2000.000] MS + spectrum 5.77
361.087
100.00%
83.137
32.89%
101.105
17.68%
141.939
1.35%
305.019
9.48%
362.165
15.65%
363.131
9.94%
m/z (Da)

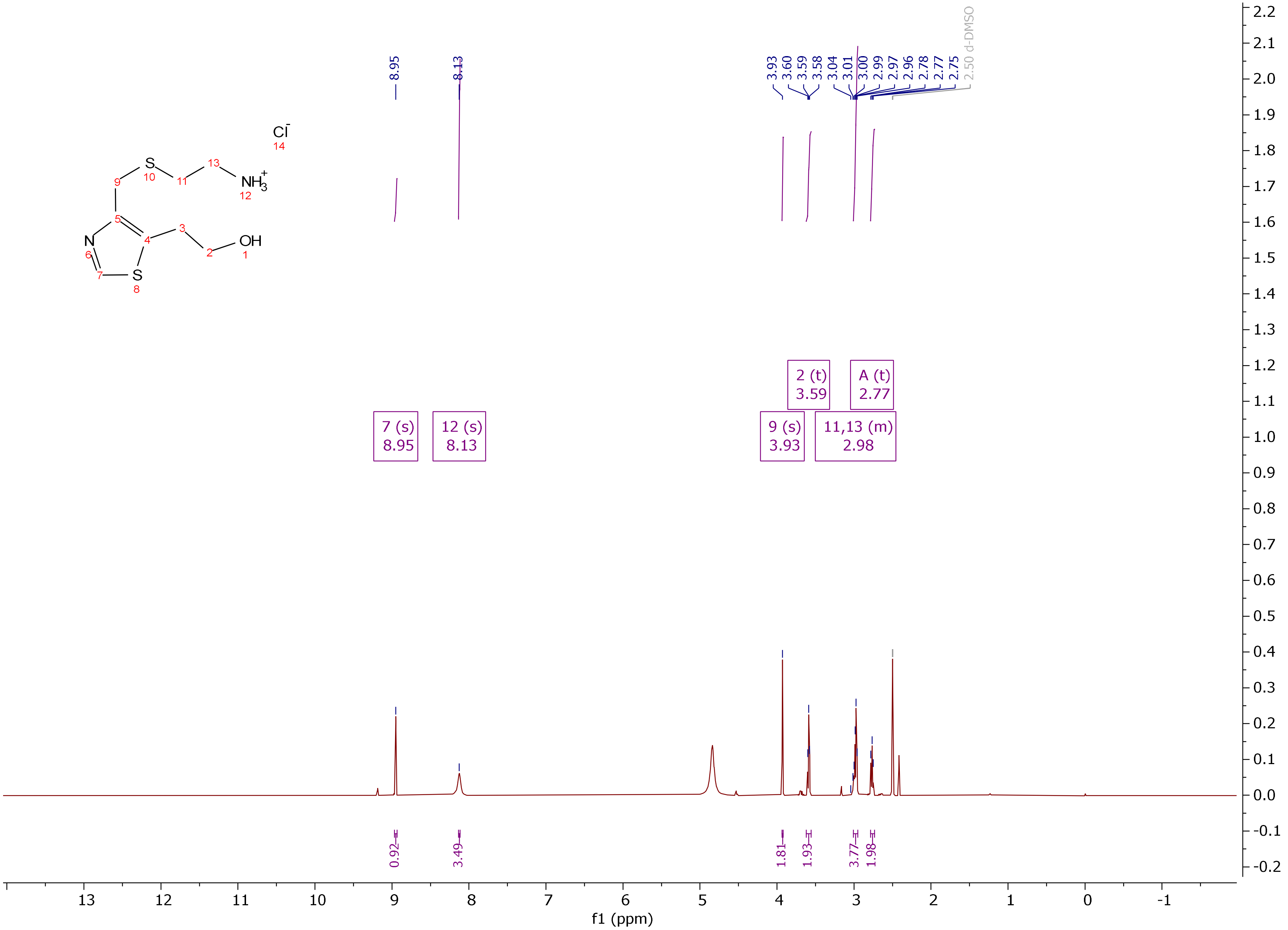

8.95
8.13
3.93
3.60
3.59
3.58
3.04
3.01
3.00
2.99
2.97
2.96
2.78
2.77
2.75
2.50 d-DMSO
7 (s)
8.95
12 (s)
8.13
9 (s)
3.93
2 (t)
3.59
11,13 (m)
2.98
A (t)
2.77
0.92
3.49
1.81
1.93
3.77
1.98
f1 (ppm)

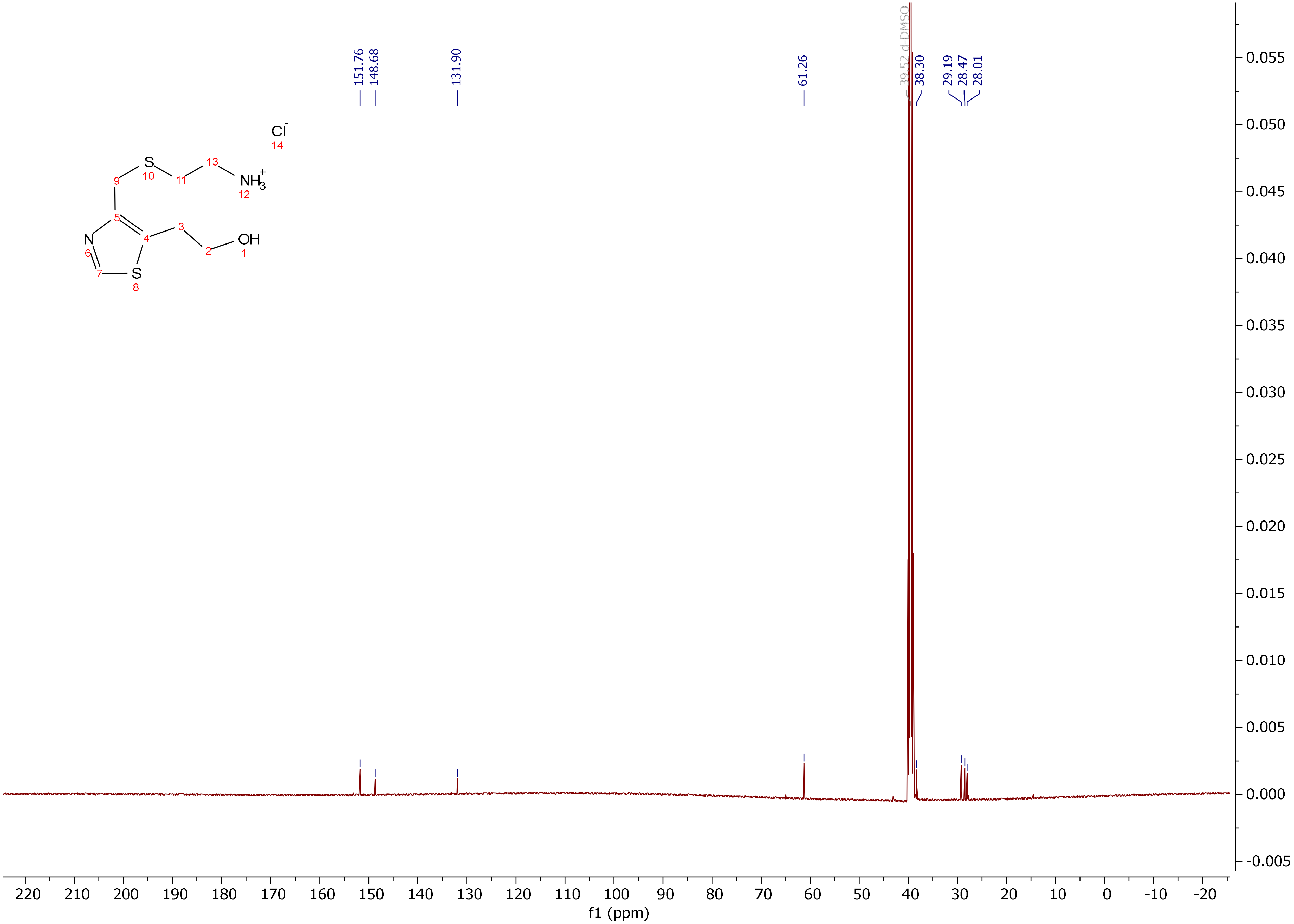
151.76
148.68
131.90
61.26
39.52 d-DMSO
38.30
29.19
28.47
28.01
Cl⁻
14
S
10
9
11
13
NH3+
12
5
4
3
2
OH
1
N
6
7
S
8
0.055
0.050
0.045
0.040
0.035
0.030
0.025
0.020
0.015
0.010
0.005
0.000
-0.005
220 210 200 190 180 170 160 150 140 130 120 110 100 90 80 70 60 50 40 30 20 10 0 -10 -20
f1 (ppm)

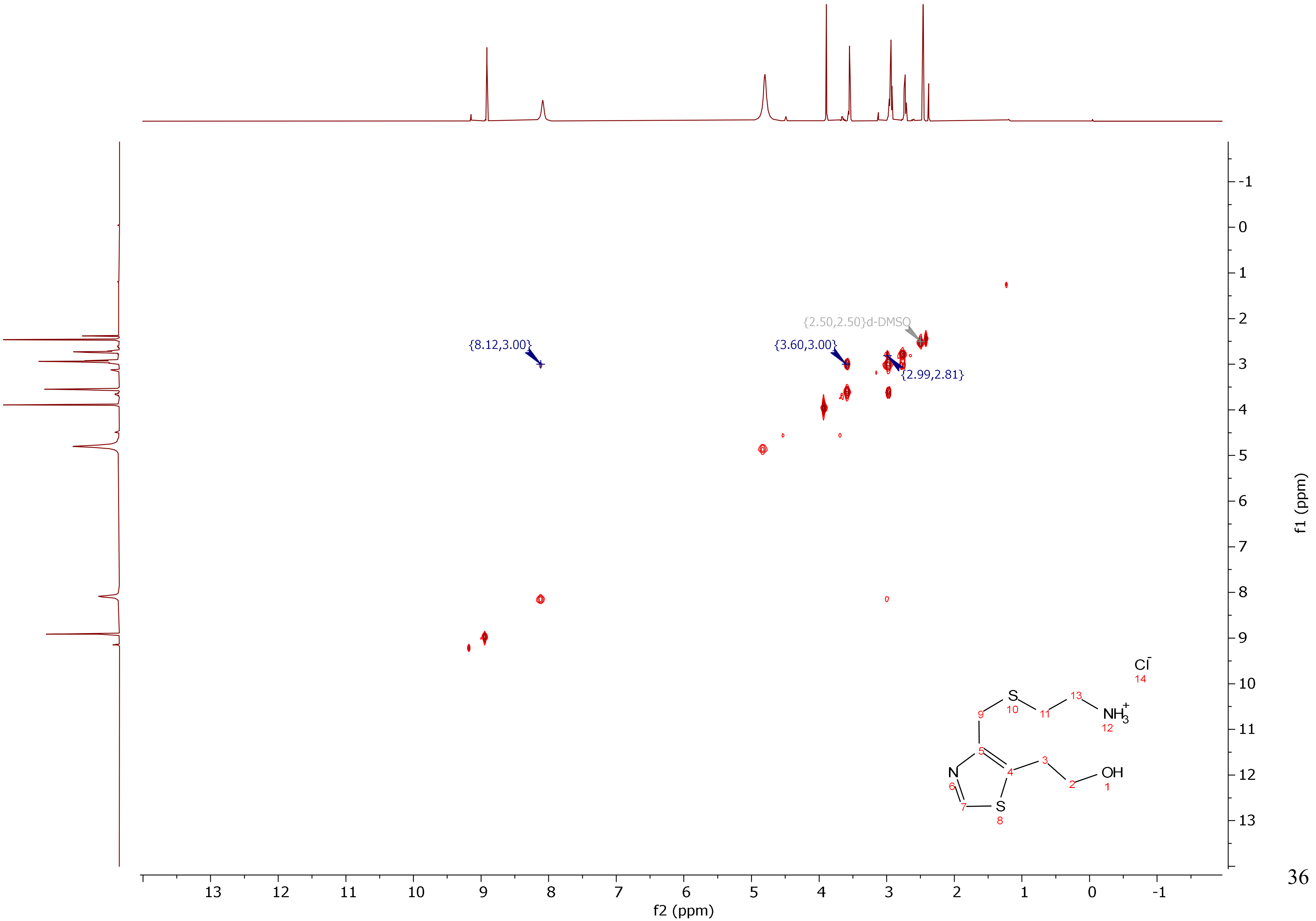
{8.12,3.00}
{3.60,3.00}
{2.50,2.50}d-DMSO
{2.99,2.81}
f2 (ppm)
f1 (ppm)
Cl
NH3
OH
N
S

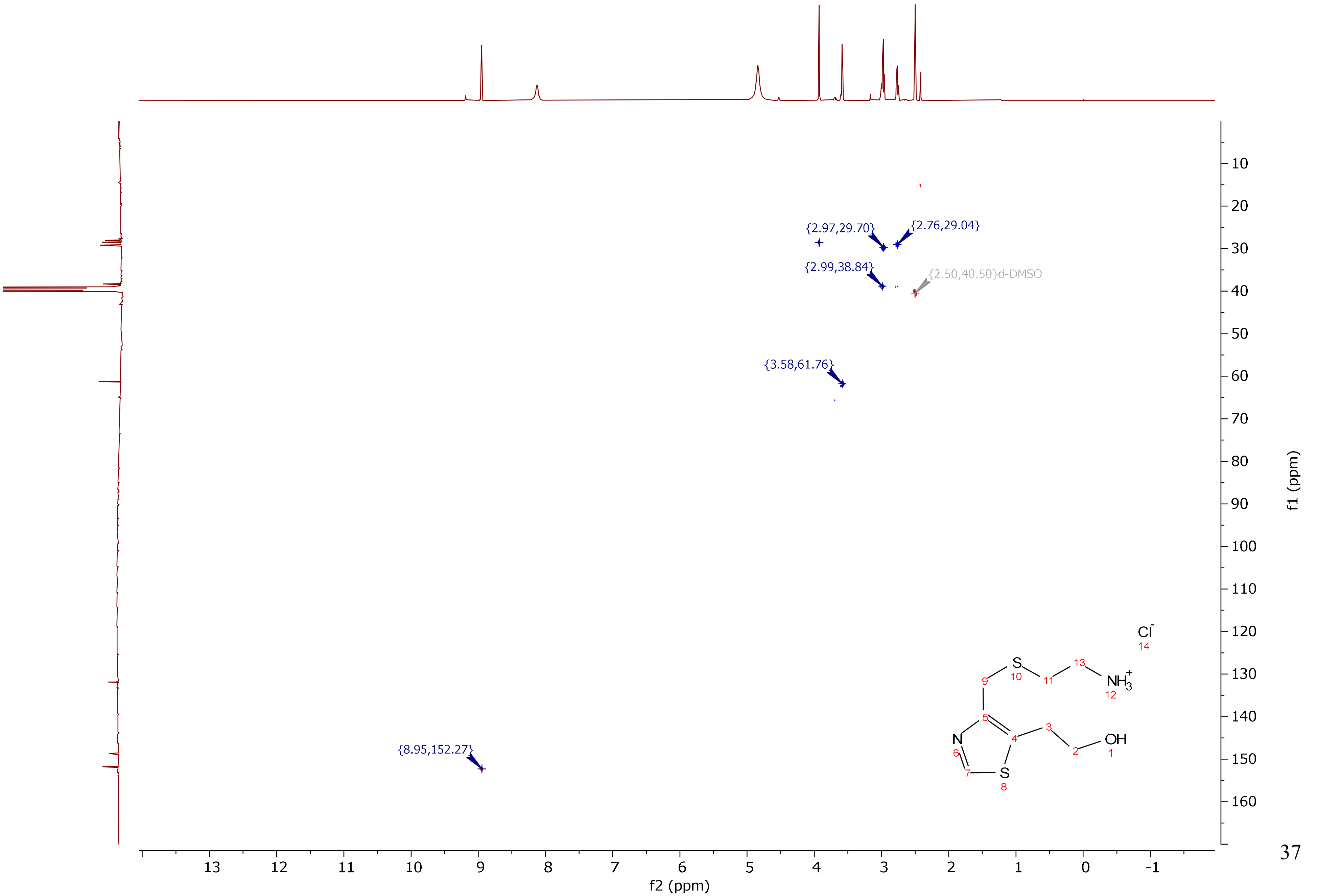
{2.97,29.70}
{2.76,29.04}
{2.99,38.84}
{2.50,40.50}d-DMSO
{3.58,61.76}
{8.95,152.27}
f2 (ppm)
f1 (ppm)
Cl⁻
NH3+
OH
N
S
S

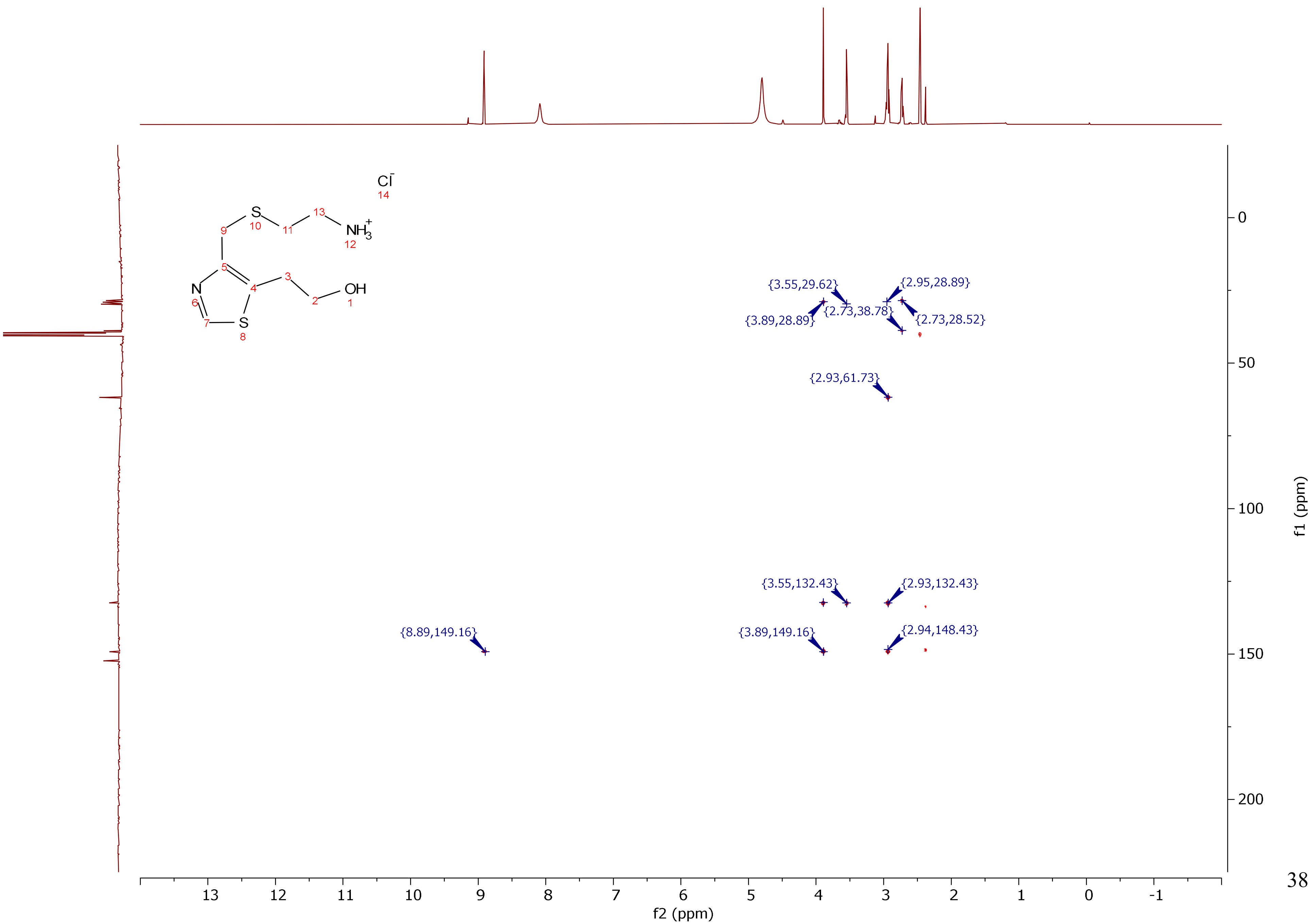
Cl⁻
14
S
10
9
11
13
NH3+
12
5
N
6
4
3
2
OH
1
7
S
8
{3.55,29.62}
{2.95,28.89}
{3.89,28.89}
{2.73,38.78}
{2.73,28.52}
{2.93,61.73}
{3.55,132.43}
{2.93,132.43}
{8.89,149.16}
{3.89,149.16}
{2.94,148.43}
f2 (ppm)
f1 (ppm)

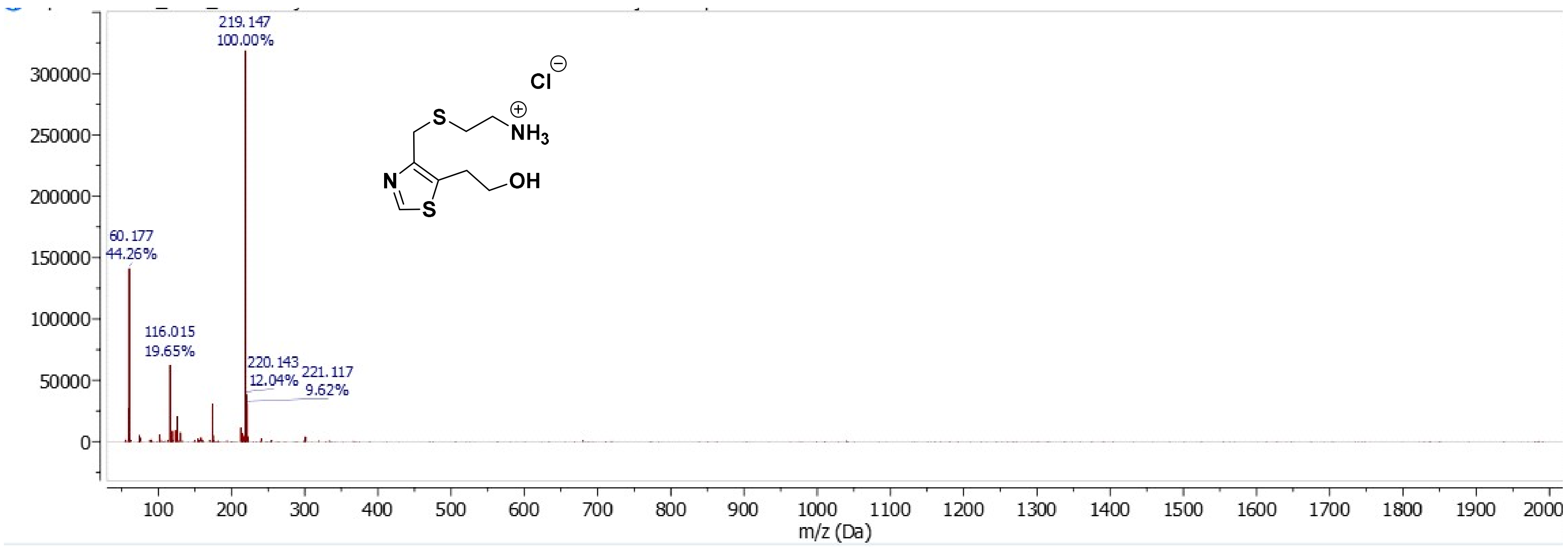
219.147
100.00%
60.177
44.26%
116.015
19.65%
220.143
12.04%
221.117
9.62%
300000
250000
200000
150000
100000
50000
0
100
200
300
400
500
600
700
800
900
1000
1100
1200
1300
1400
1500
1600
1700
1800
1900
2000
m/z (Da)
Cl
S
NH3
N
S
OH

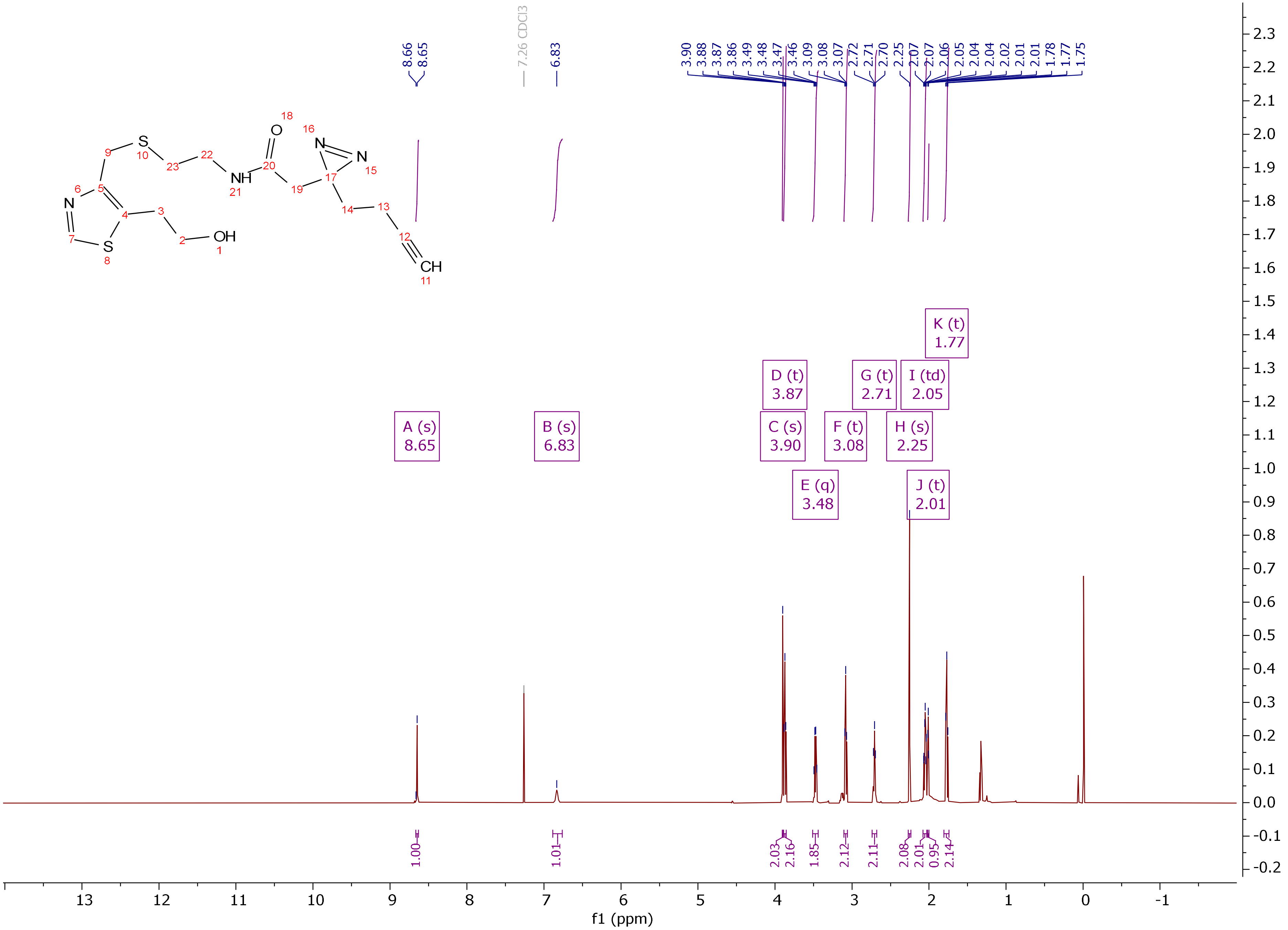

A (s)
8.65
B (s)
6.83
C (s)
3.90
D (t)
3.87
E (q)
3.48
F (t)
3.08
G (t)
2.71
H (s)
2.25
I (td)
2.05
J (t)
2.01
K (t)
1.77
7.26 CDCl3
f1 (ppm)

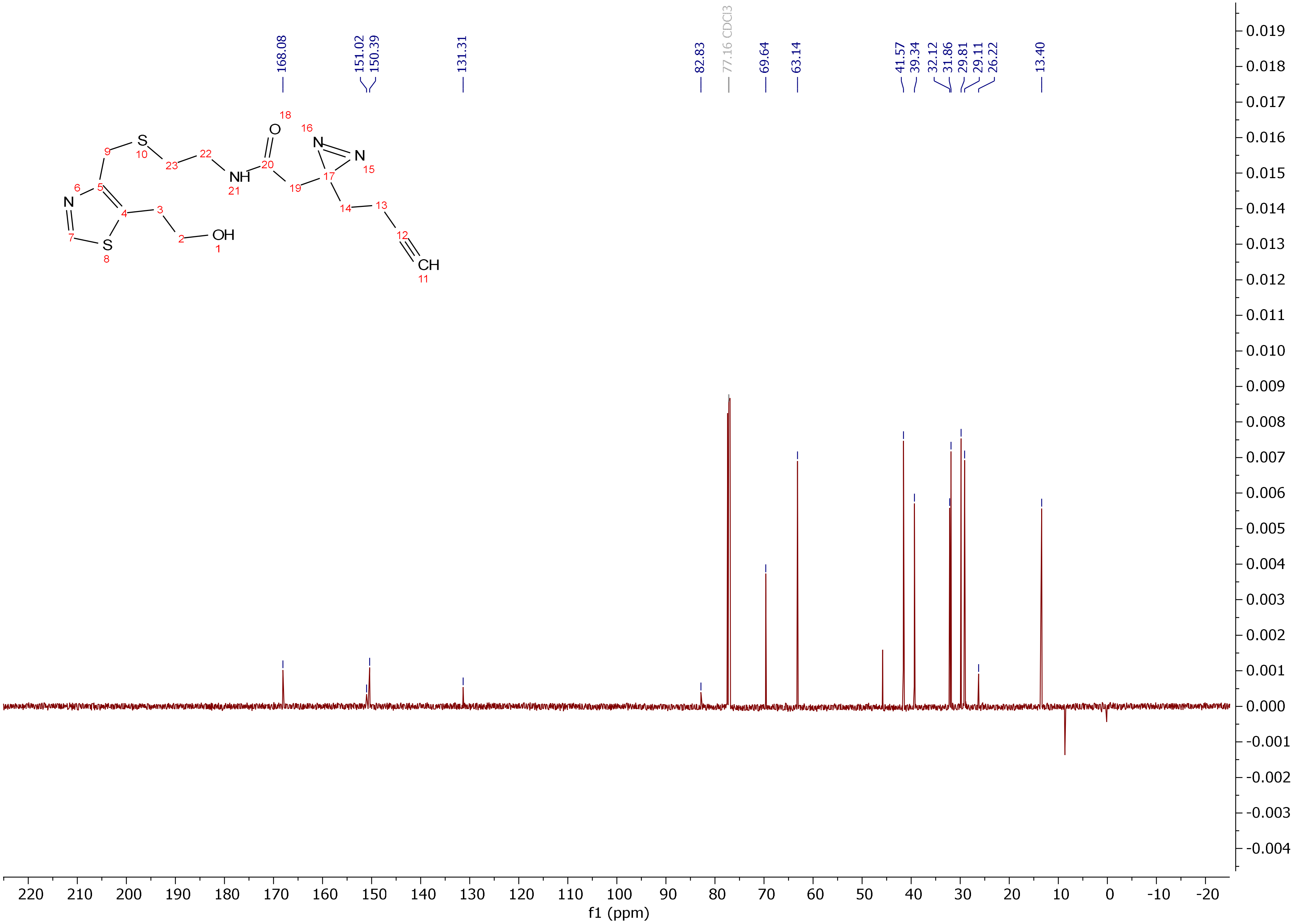

168.08
151.02
150.39
131.31
82.83
77.16 CDCl3
69.64
63.14
41.57
39.34
32.12
31.86
29.81
29.11
26.22
13.40
f1 (ppm)

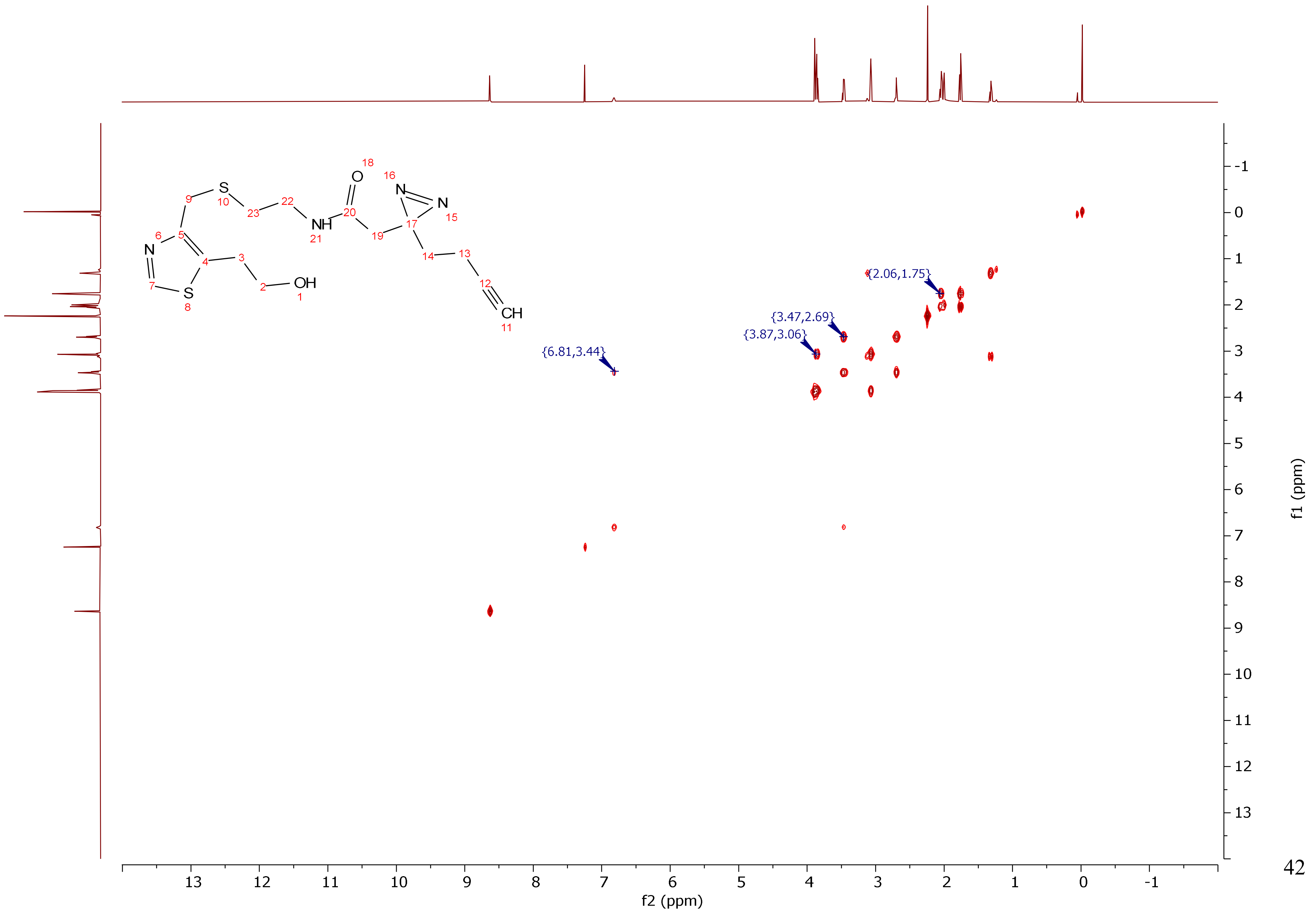

18
16
O
N
N
15
S
9
10
23
22
NH
21
20
19
17
14
13
6
5
N
4
3
7
S
8
2
OH
1
12
CH
11
{2.06,1.75}
{3.47,2.69}
{3.87,3.06}
{6.81,3.44}
f2 (ppm)
f1 (ppm)

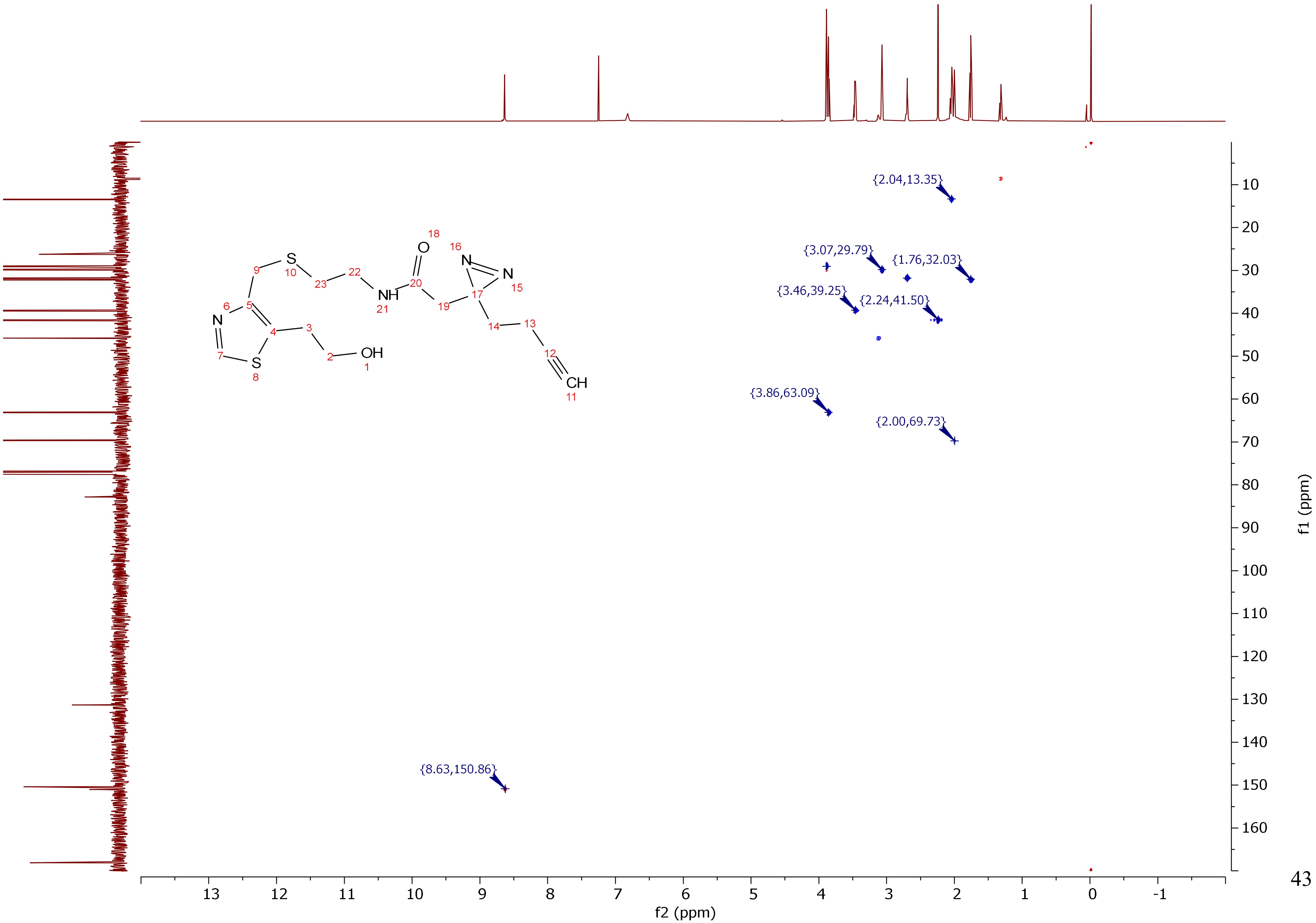
{2.04,13.35}
{3.07,29.79}
{1.76,32.03}
{3.46,39.25}
{2.24,41.50}
{3.86,63.09}
{2.00,69.73}
{8.63,150.86}
f1 (ppm)
f2 (ppm)
OH
NH
S
N
O
CH

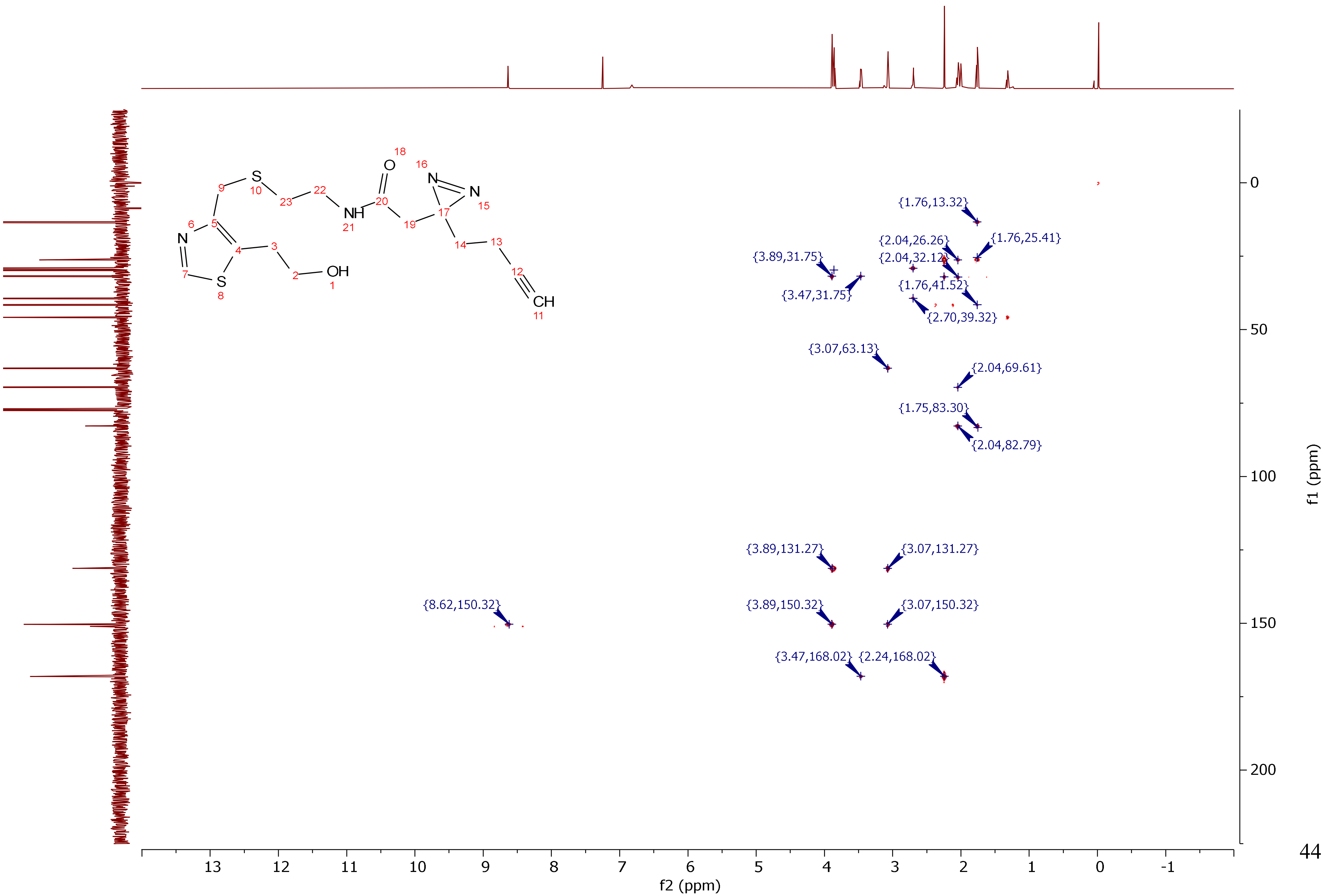
{1.76,13.32}
{2.04,26.26}
{1.76,25.41}
{3.89,31.75}
{2.04,32.12}
{3.47,31.75}
{1.76,41.52}
{2.70,39.32}
{3.07,63.13}
{2.04,69.61}
{1.75,83.30}
{2.04,82.79}
{3.89,131.27}
{3.07,131.27}
{8.62,150.32}
{3.89,150.32}
{3.07,150.32}
{3.47,168.02}
{2.24,168.02}
f2 (ppm)
f1 (ppm)

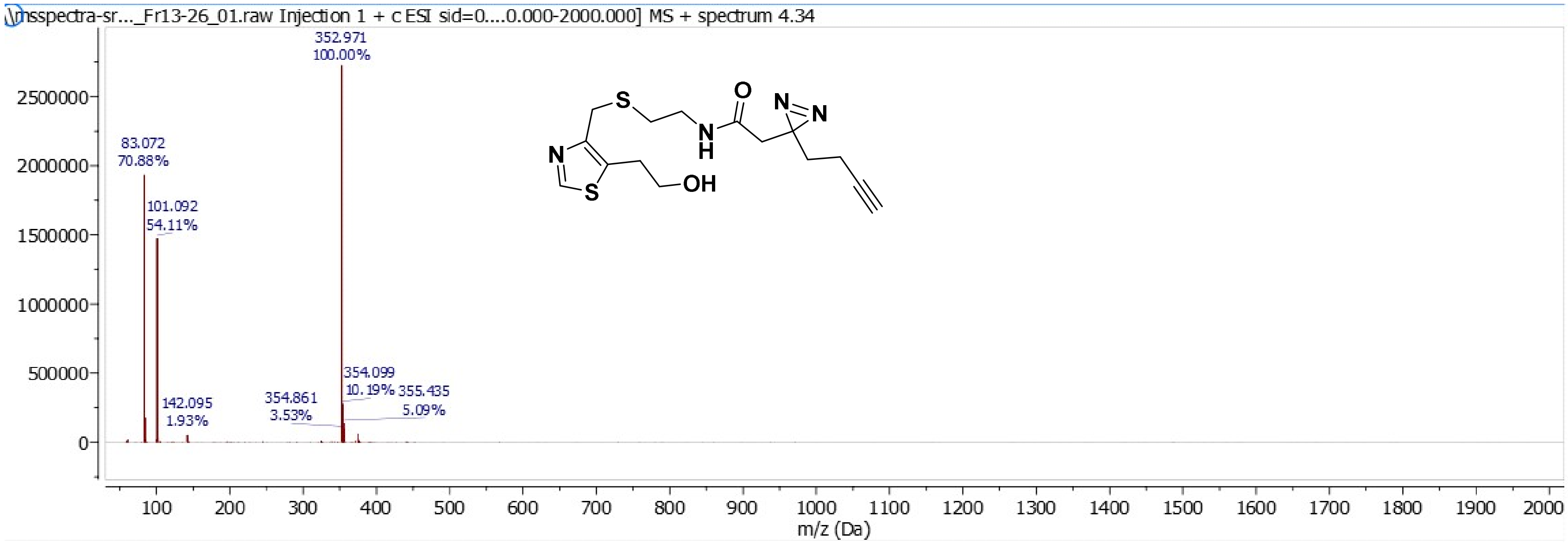
\msspectra-sr..._Fr13-26_01.raw Injection 1 + c ESI sid=0....0.000-2000.000] MS + spectrum 4.34
352.971
100.00%
83.072
70.88%
101.092
54.11%
142.095
1.93%
354.861
3.53%
354.099
10.19%
355.435
5.09%
2500000
2000000
1500000
1000000
500000
0
100
200
300
400
500
600
700
800
900
1000
1100
1200
1300
1400
1500
1600
1700
1800
1900
2000
m/z (Da)
S
N
S
OH
N
H
O
N
N

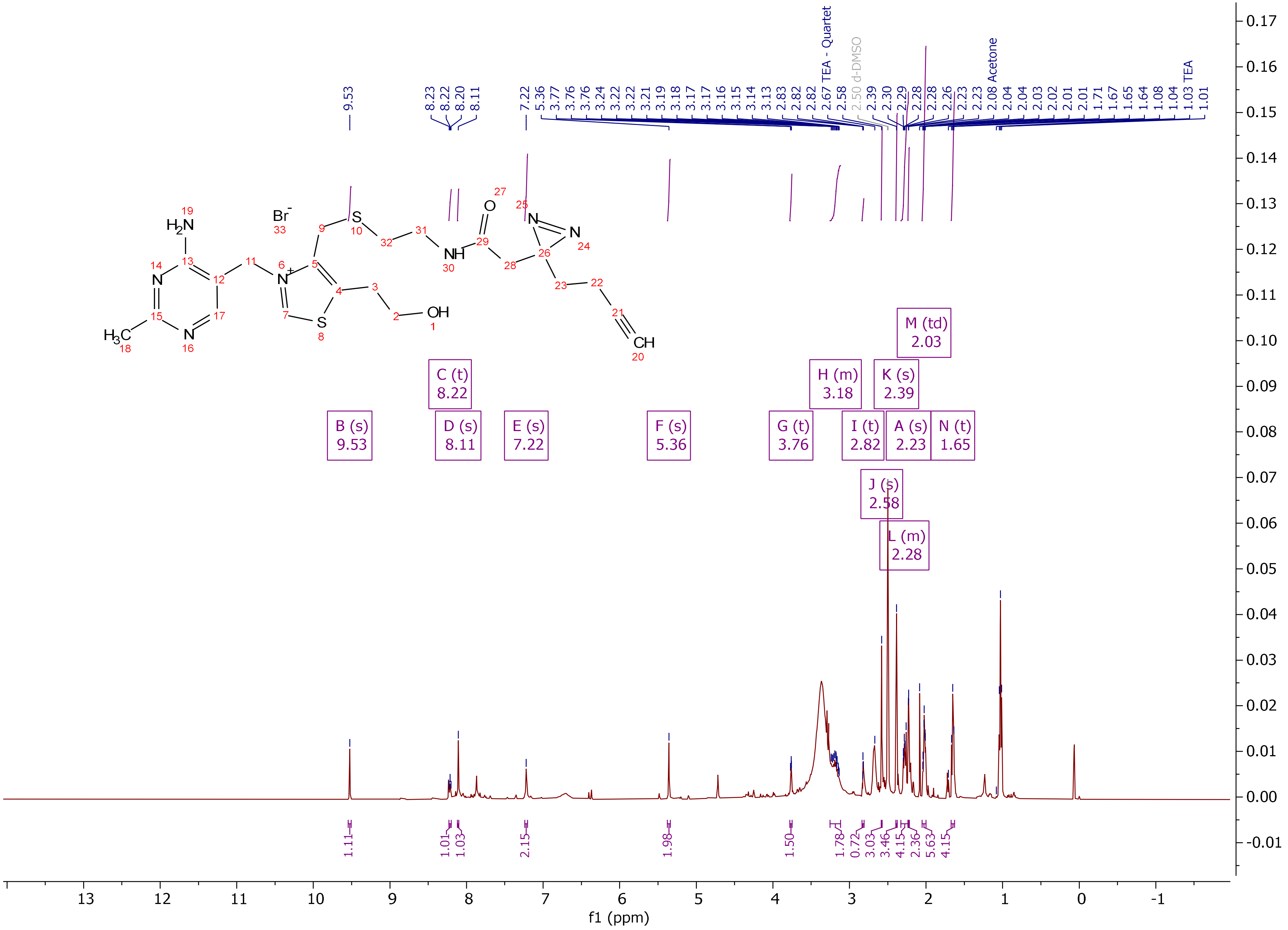

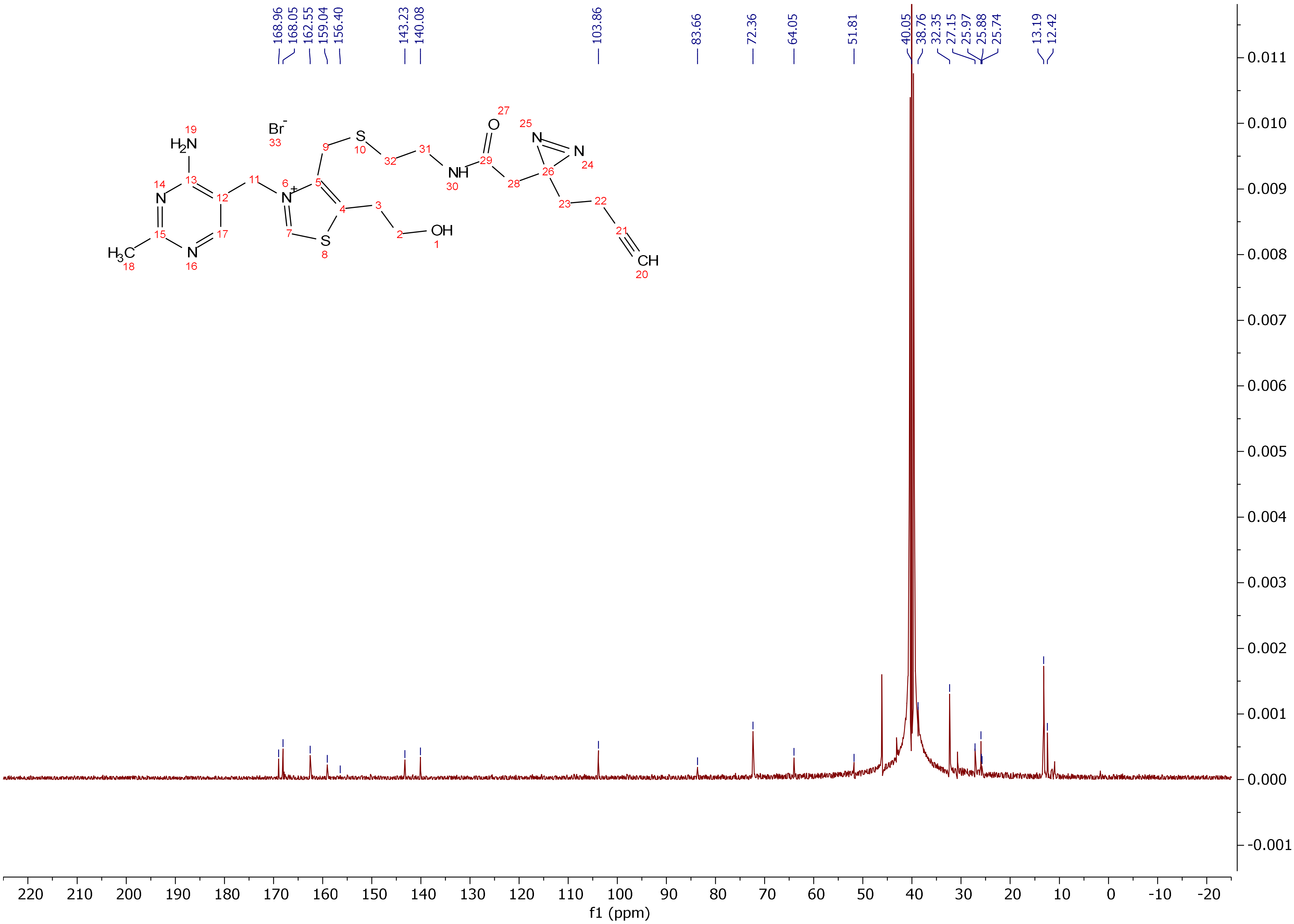

168.96
168.05
162.55
159.04
156.40
143.23
140.08
103.86
83.66
72.36
64.05
51.81
40.05
38.76
32.35
27.15
25.97
25.88
25.74
13.19
12.42
f1 (ppm)

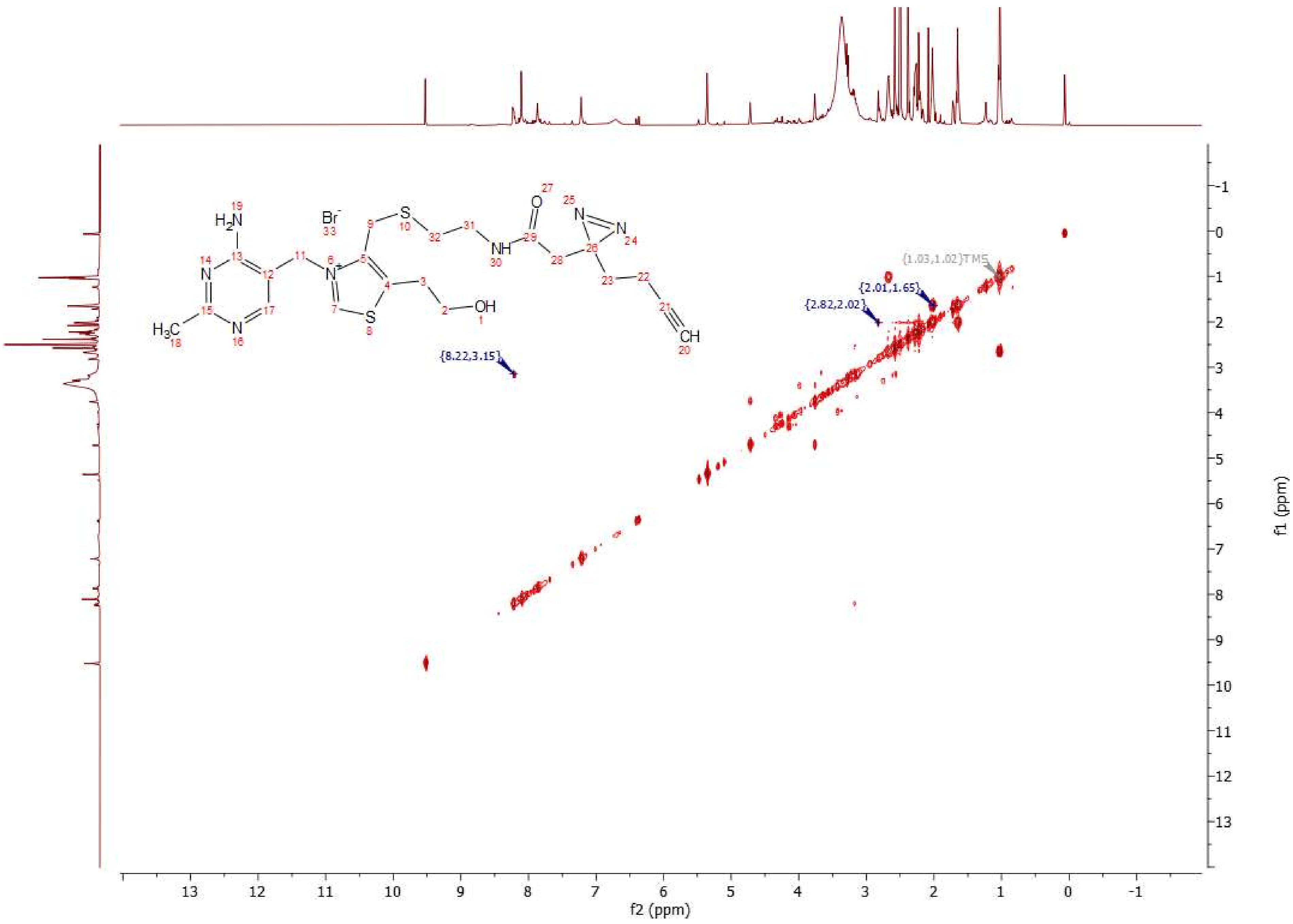

{8.22,3.15}
{2.01,1.65}
{2.82,2.02}
{1.03,1.02}TMS
f2 (ppm)
f1 (ppm)

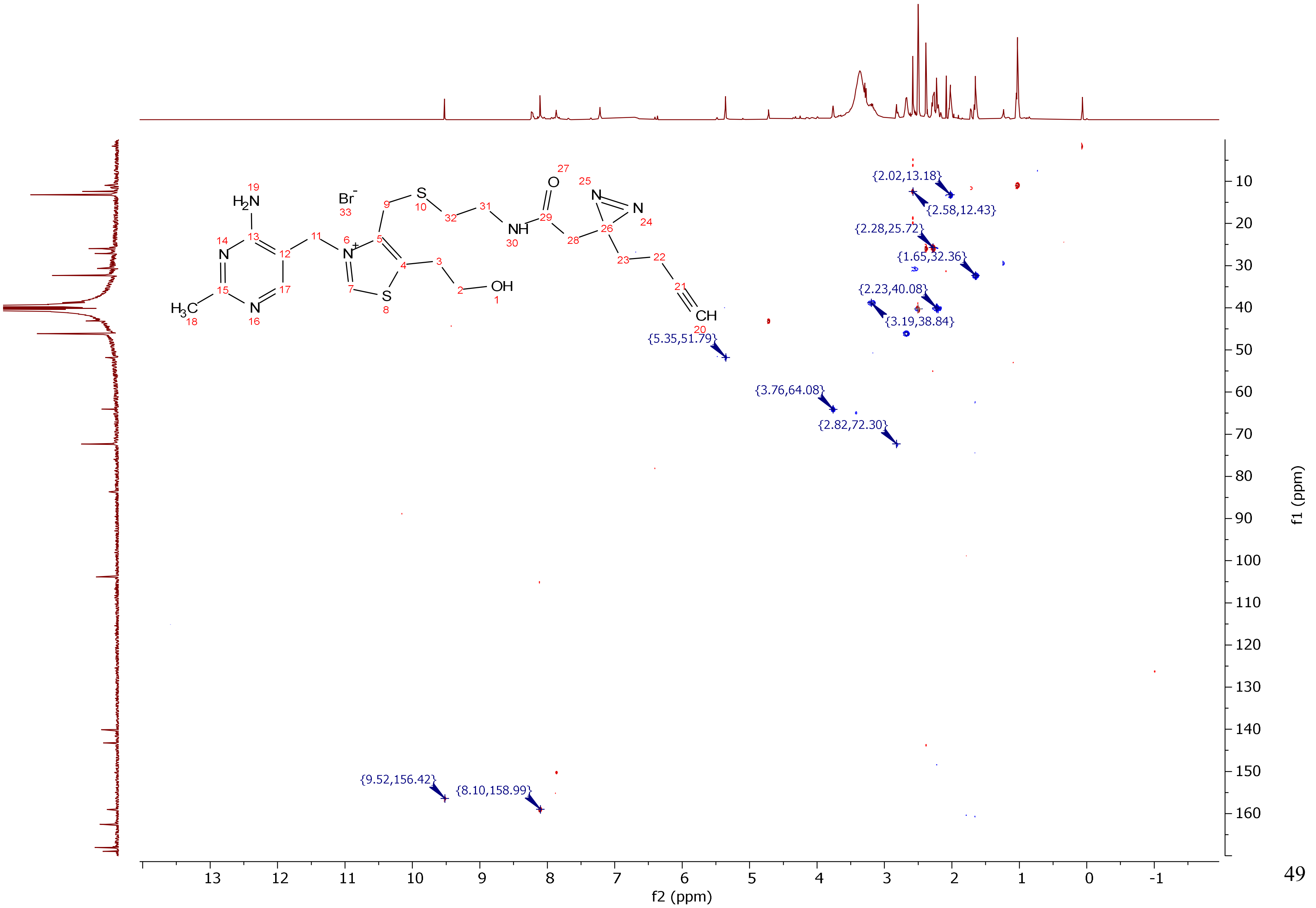

{2.02,13.18}
{2.58,12.43}
{2.28,25.72}
{1.65,32.36}
{2.23,40.08}
{3.19,38.84}
{5.35,51.79}
{3.76,64.08}
{2.82,72.30}
{9.52,156.42}
{8.10,158.99}
f2 (ppm)
f1 (ppm)

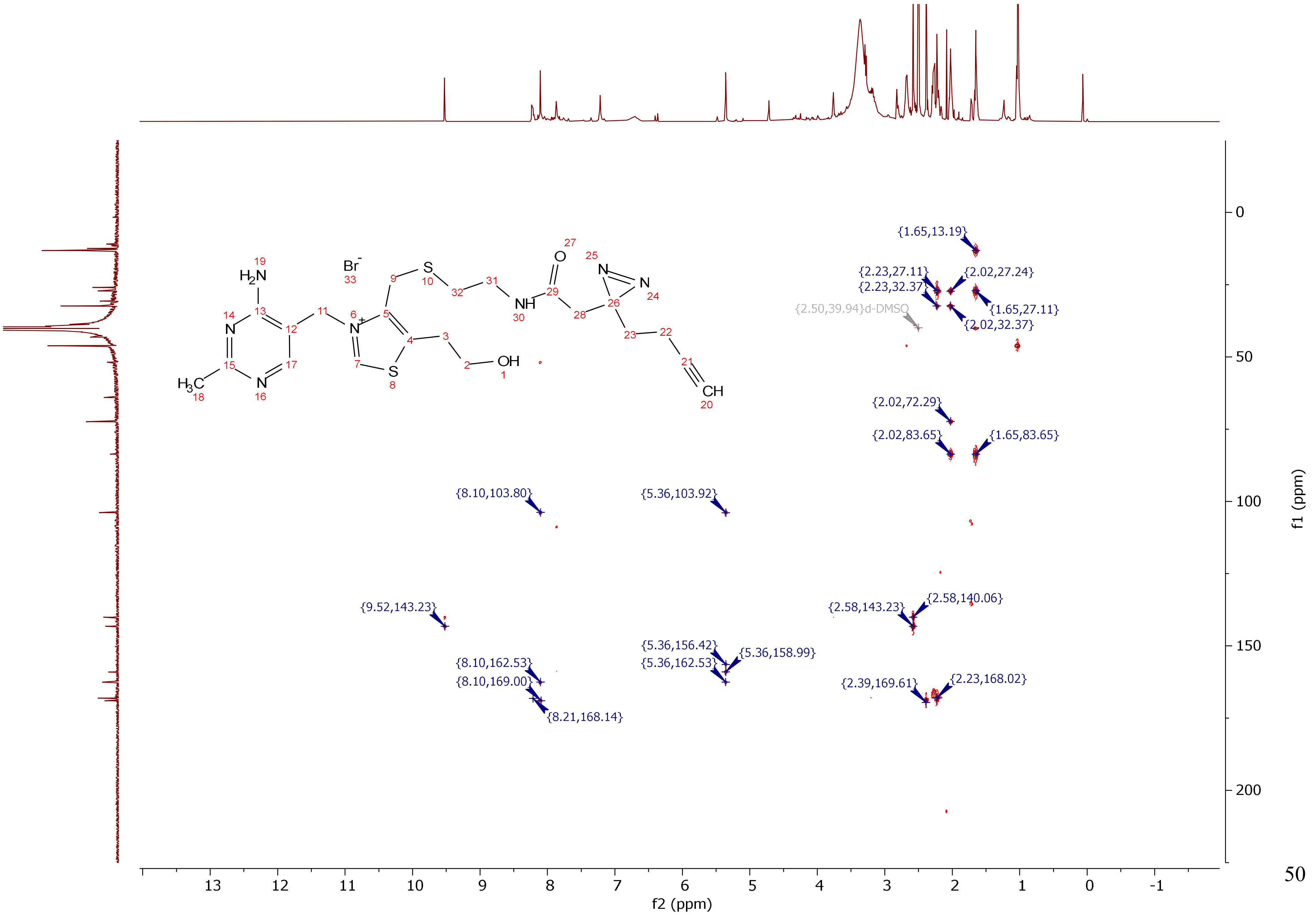

{1.65,13.19}
{2.23,27.11}
{2.02,27.24}
{2.23,32.37}
{1.65,27.11}
{2.50,39.94}d-DMSO
{2.02,32.37}
{2.02,72.29}
{2.02,83.65}
{1.65,83.65}
{8.10,103.80}
{5.36,103.92}
{9.52,143.23}
{2.58,143.23}
{2.58,140.06}
{5.36,156.42}
{5.36,158.99}
{5.36,162.53}
{8.10,162.53}
{8.10,169.00}
{8.21,168.14}
{2.39,169.61}
{2.23,168.02}
Br-
H2N
N
N
H3C
N+
S
S
OH
NH
O
N
N
CH
f2 (ppm)
f1 (ppm)

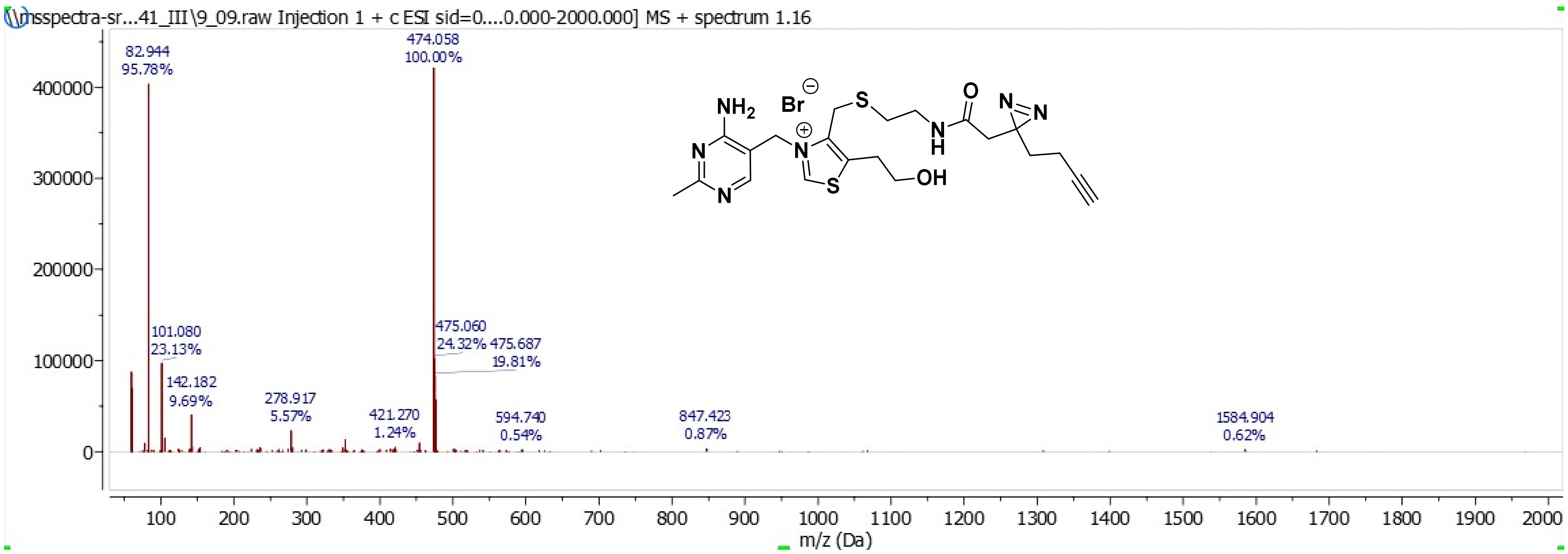
\\msspectra-sr...41_III\9_09.raw Injection 1 + c ESI sid=0....0.000-2000.000] MS + spectrum 1.16
82.944
95.78%
474.058
100.00%
101.080
23.13%
142.182
9.69%
278.917
5.57%
421.270
1.24%
475.060
24.32%
475.687
19.81%
594.740
0.54%
847.423
0.87%
1584.904
0.62%
m/z (Da)
NH2
Br
N
N
S
S
OH
N
H
O
N
N

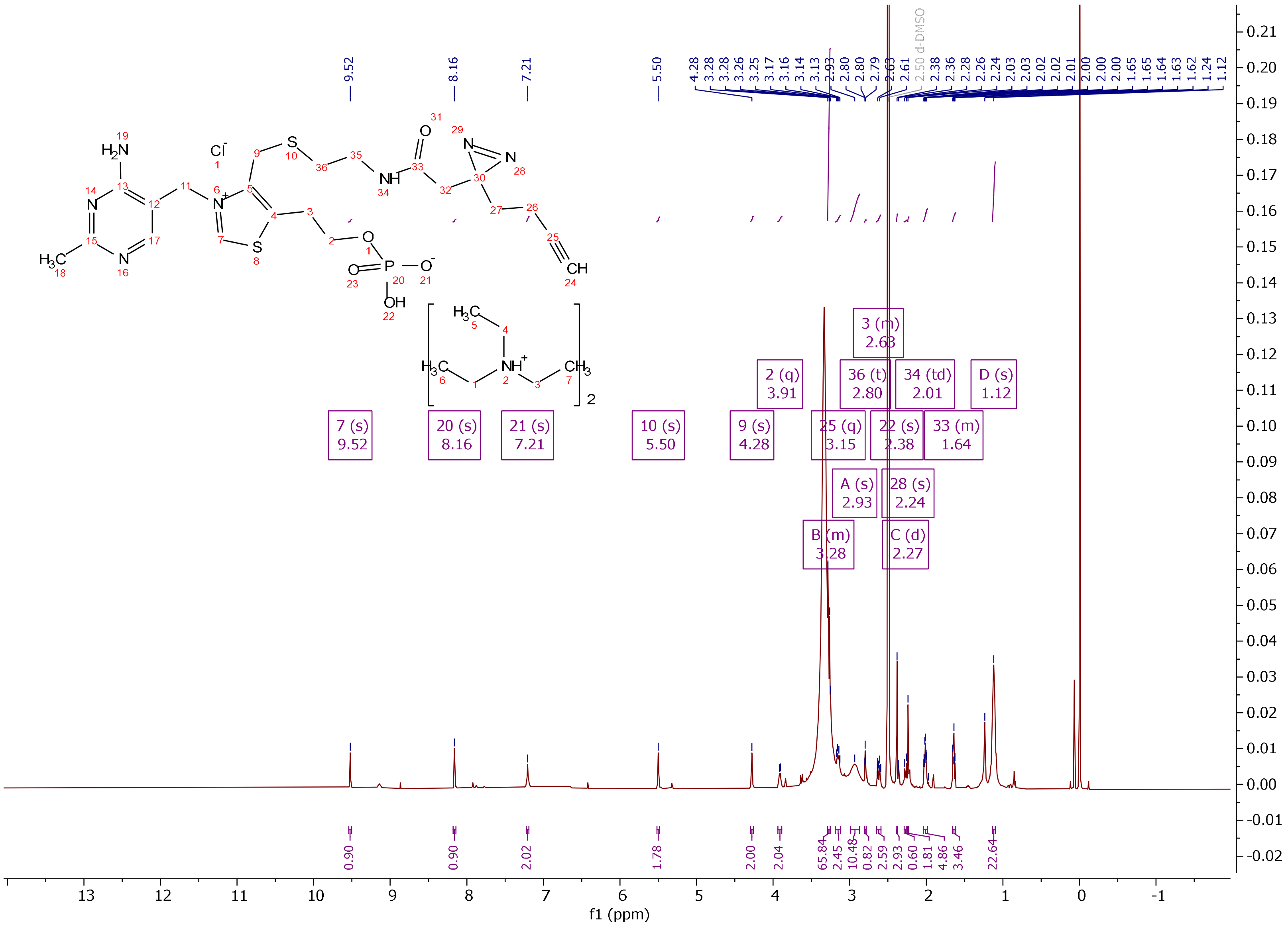

7 (s) 9.52
20 (s) 8.16
21 (s) 7.21
10 (s) 5.50
9 (s) 4.28
2 (q) 3.91
25 (q) 3.15
B (m) 3.28
A (s) 2.93
36 (t) 2.80
3 (m) 2.63
22 (s) 2.38
C (d) 2.27
28 (s) 2.24
34 (td) 2.01
33 (m) 1.64
D (s) 1.12
2.50 d-DMSO
f1 (ppm)

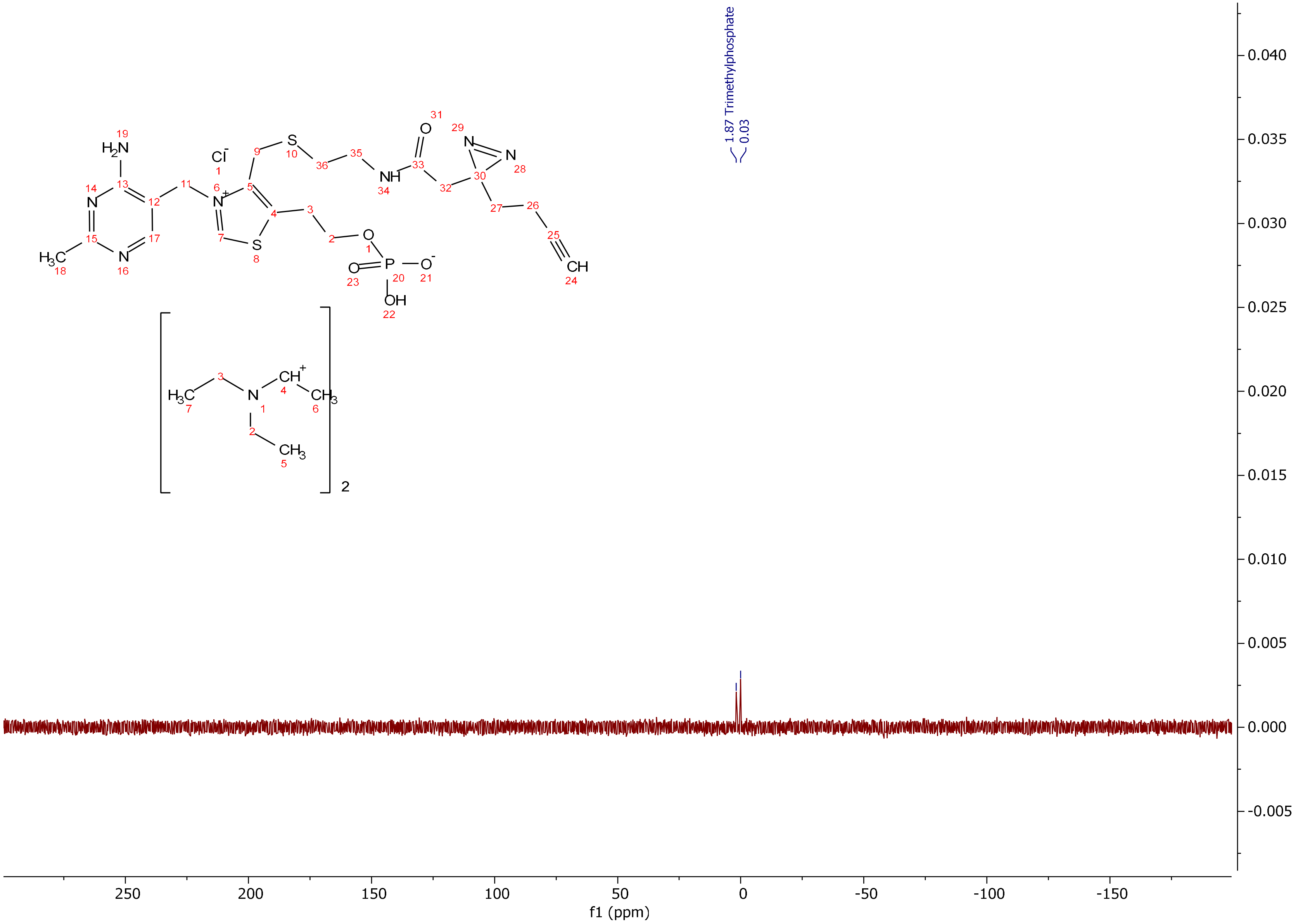
1.87 Trimethylphosphate
0.03
f1 (ppm)

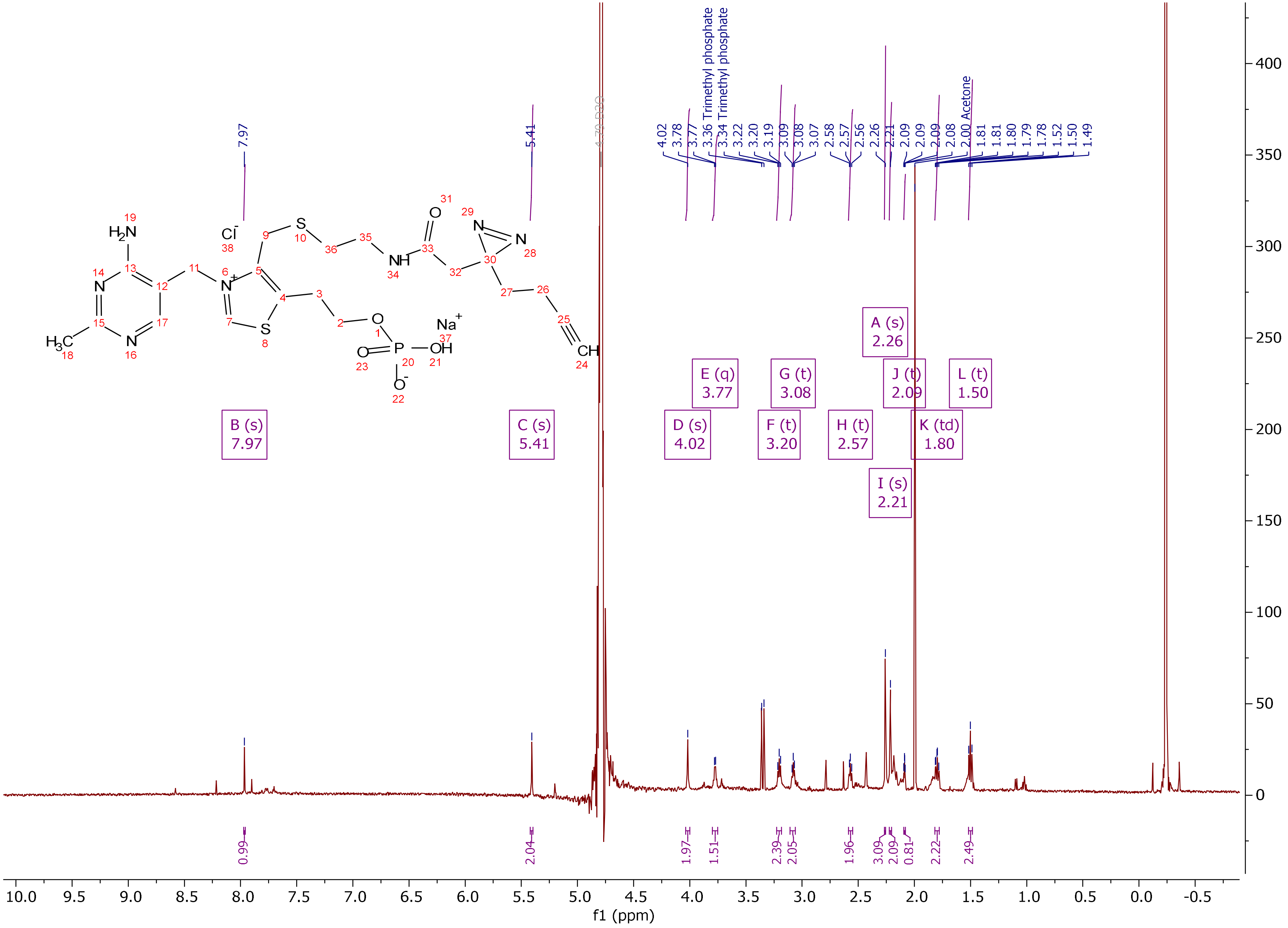

B (s) 7.97
C (s) 5.41
D (s) 4.02
E (q) 3.77
F (t) 3.20
G (t) 3.08
H (t) 2.57
A (s) 2.26
I (s) 2.21
J (t) 2.09
K (td) 1.80
L (t) 1.50
Trimethyl phosphate
Acetone
f1 (ppm)

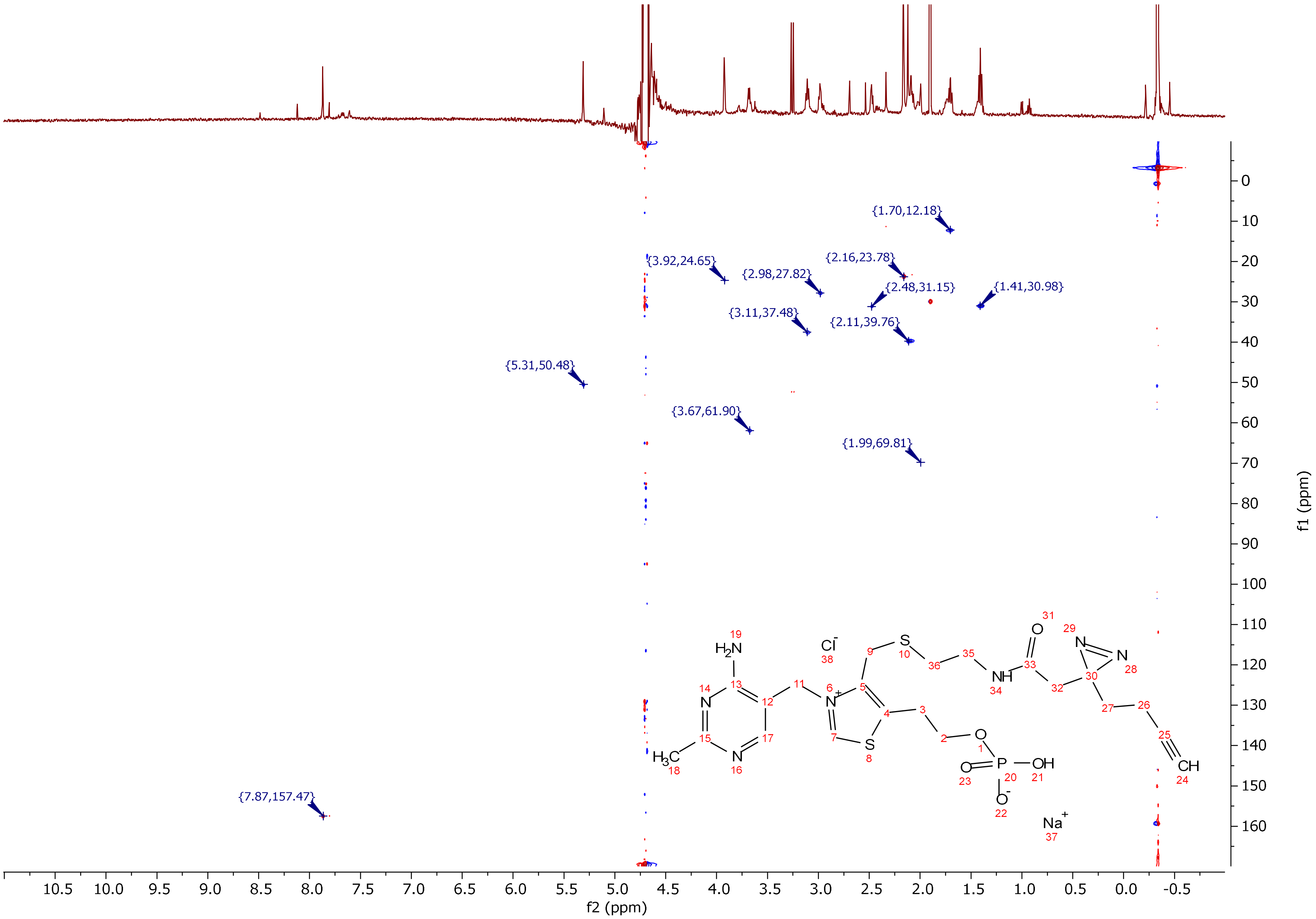
{1.70,12.18}
{3.92,24.65}
{2.98,27.82}
{2.16,23.78}
{2.48,31.15}
{1.41,30.98}
{3.11,37.48}
{2.11,39.76}
{5.31,50.48}
{3.67,61.90}
{1.99,69.81}
{7.87,157.47}
f1 (ppm)
f2 (ppm)
Cl⁻
Na⁺

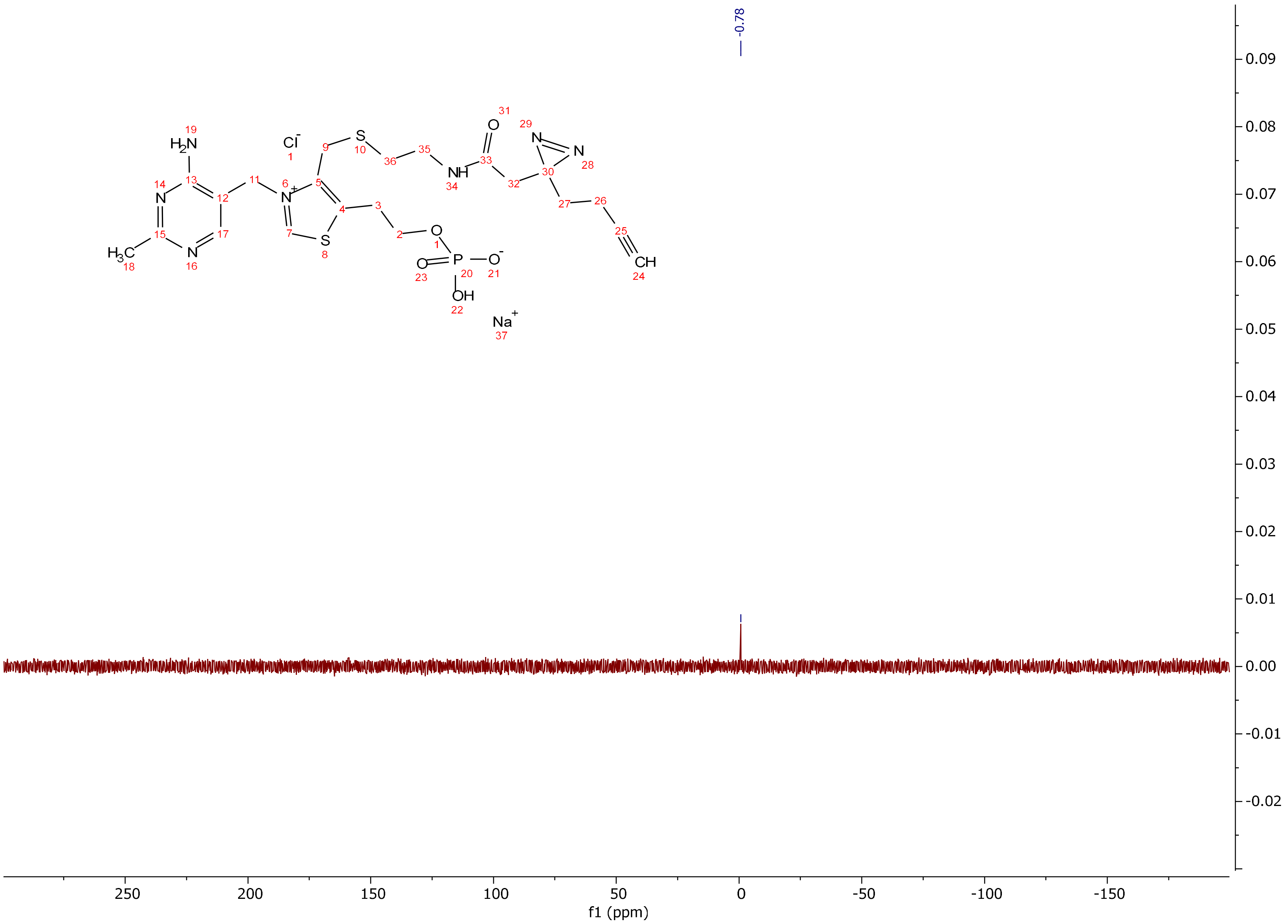
-0.78
f1 (ppm)

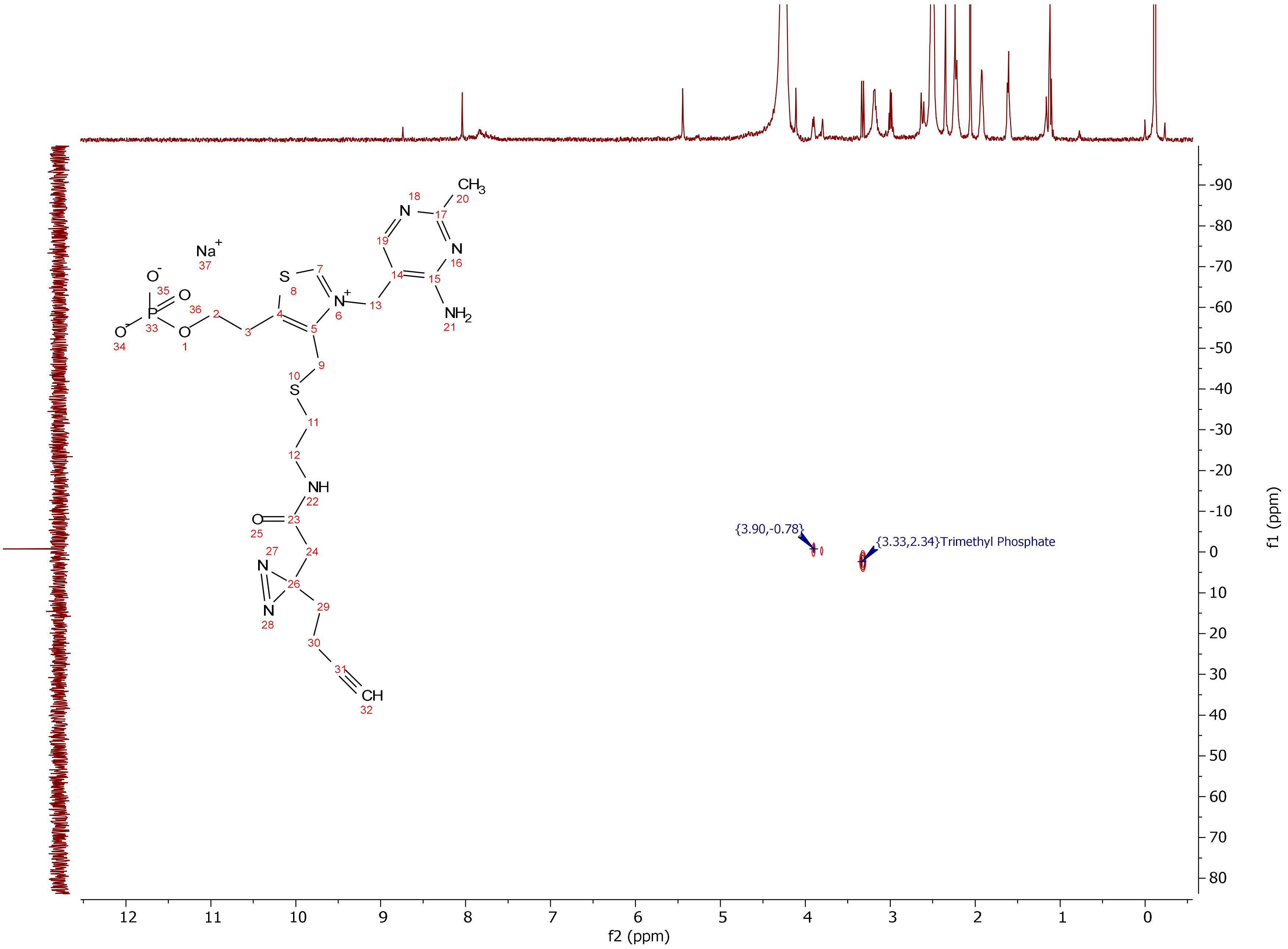
{3.90,-0.78}
{3.33,2.34}Trimethyl Phosphate
f2 (ppm)
f1 (ppm)
Na+
CH3
NH2
NH
CH

LC-MS spectrum

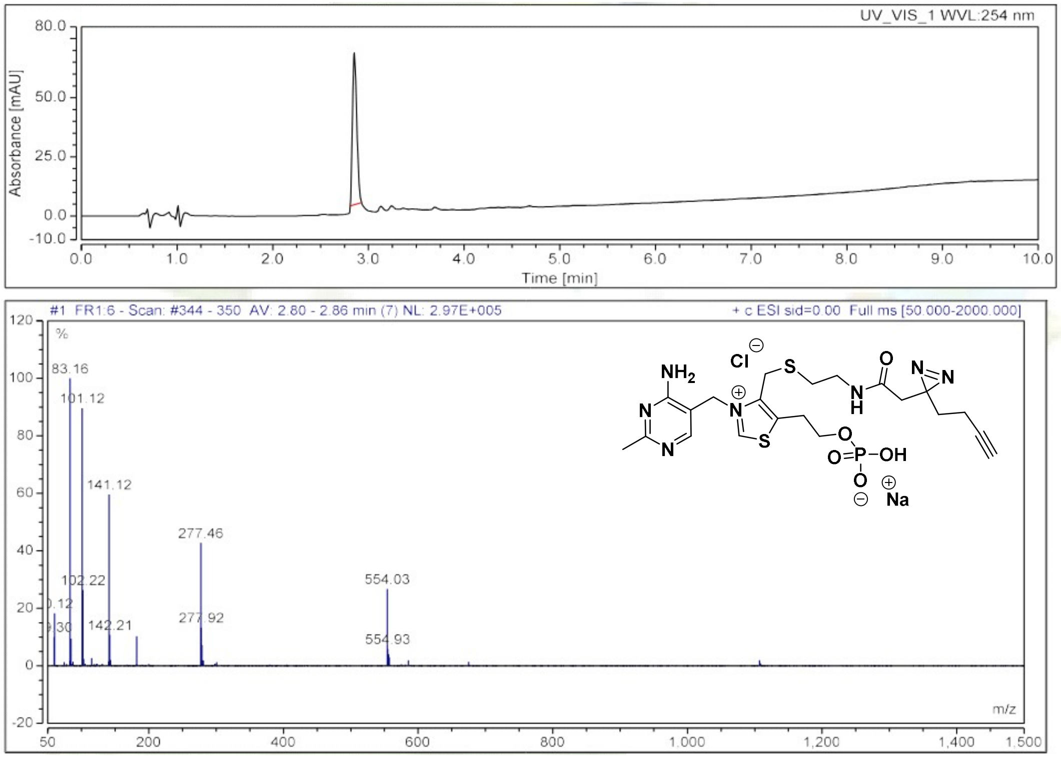

HRMS spectrum

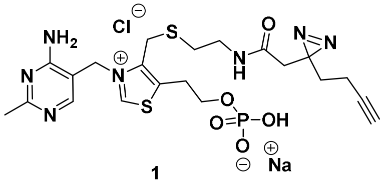

1

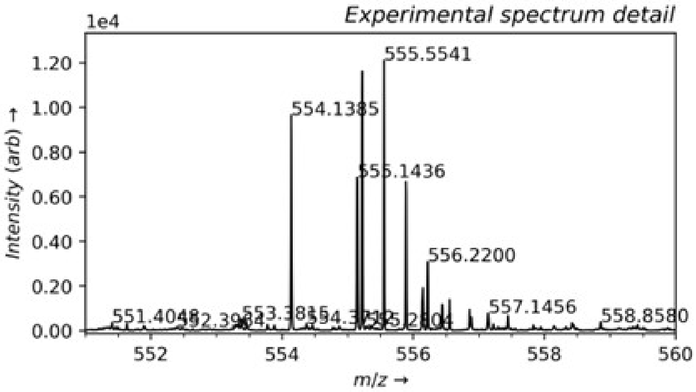

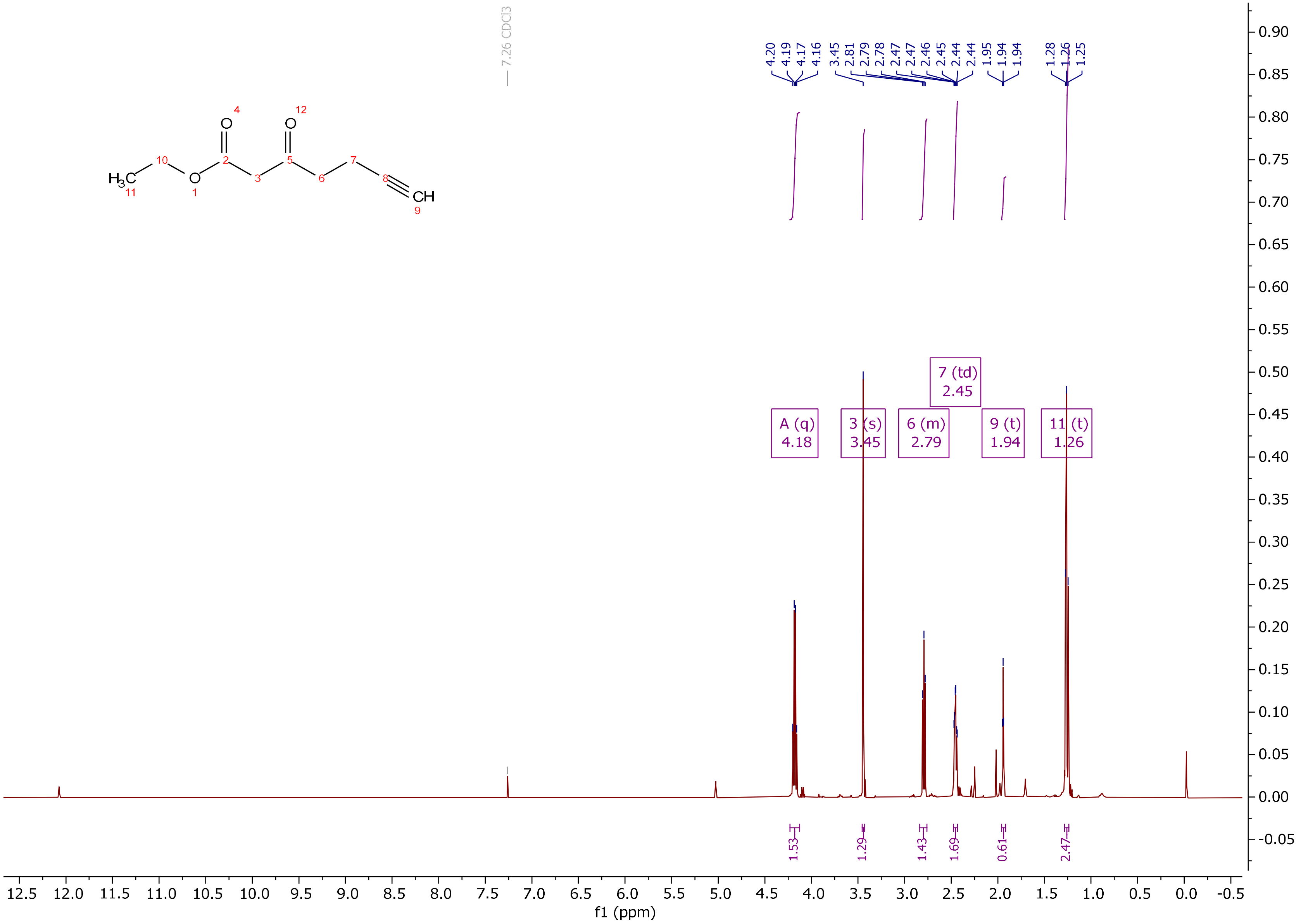
7.26 CDCl3
4.20
4.19
4.17
4.16
3.45
2.81
2.79
2.78
2.47
2.47
2.46
2.45
2.44
2.44
1.95
1.94
1.94
1.28
1.26
1.25
A (q)
4.18
3 (s)
3.45
6 (m)
2.79
7 (td)
2.45
9 (t)
1.94
11 (t)
1.26
1.53
1.29
1.43
1.69
0.61
2.47
f1 (ppm)

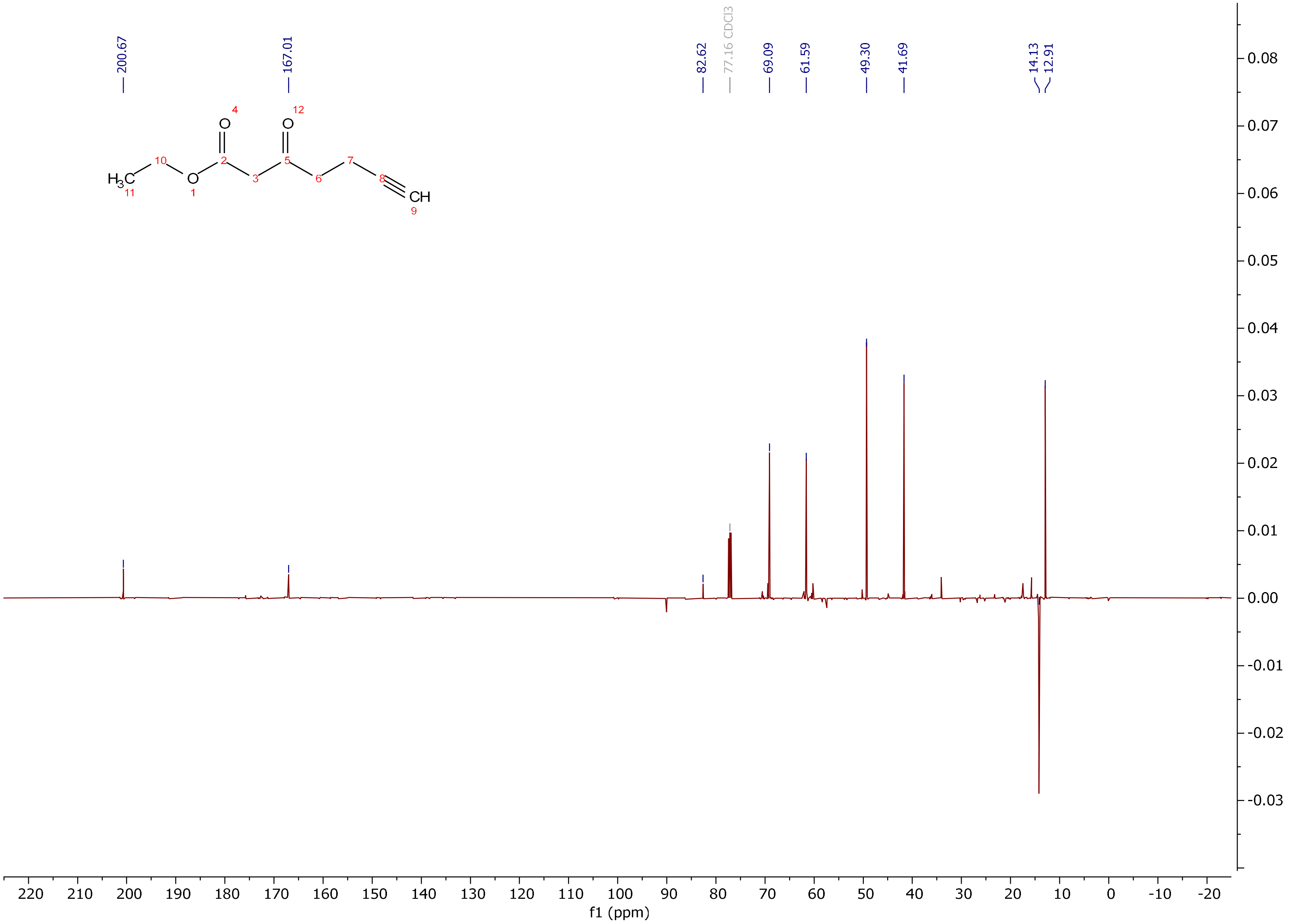

200.67
167.01
82.62
77.16 CDCl3
69.09
61.59
49.30
41.69
14.13
12.91
f1 (ppm)

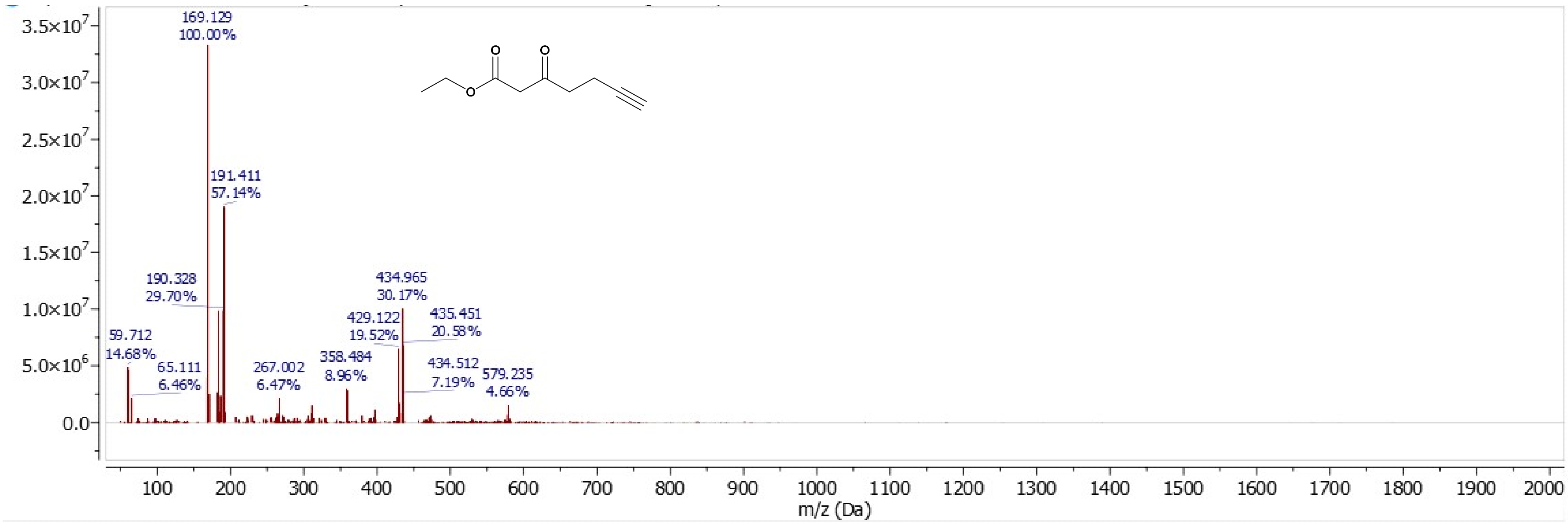

169.129
100.00%
191.411
57.14%
190.328
29.70%
434.965
30.17%
429.122
19.52%
435.451
20.58%
59.712
14.68%
65.111
6.46%
267.002
6.47%
358.484
8.96%
434.512
7.19%
579.235
4.66%
m/z (Da)

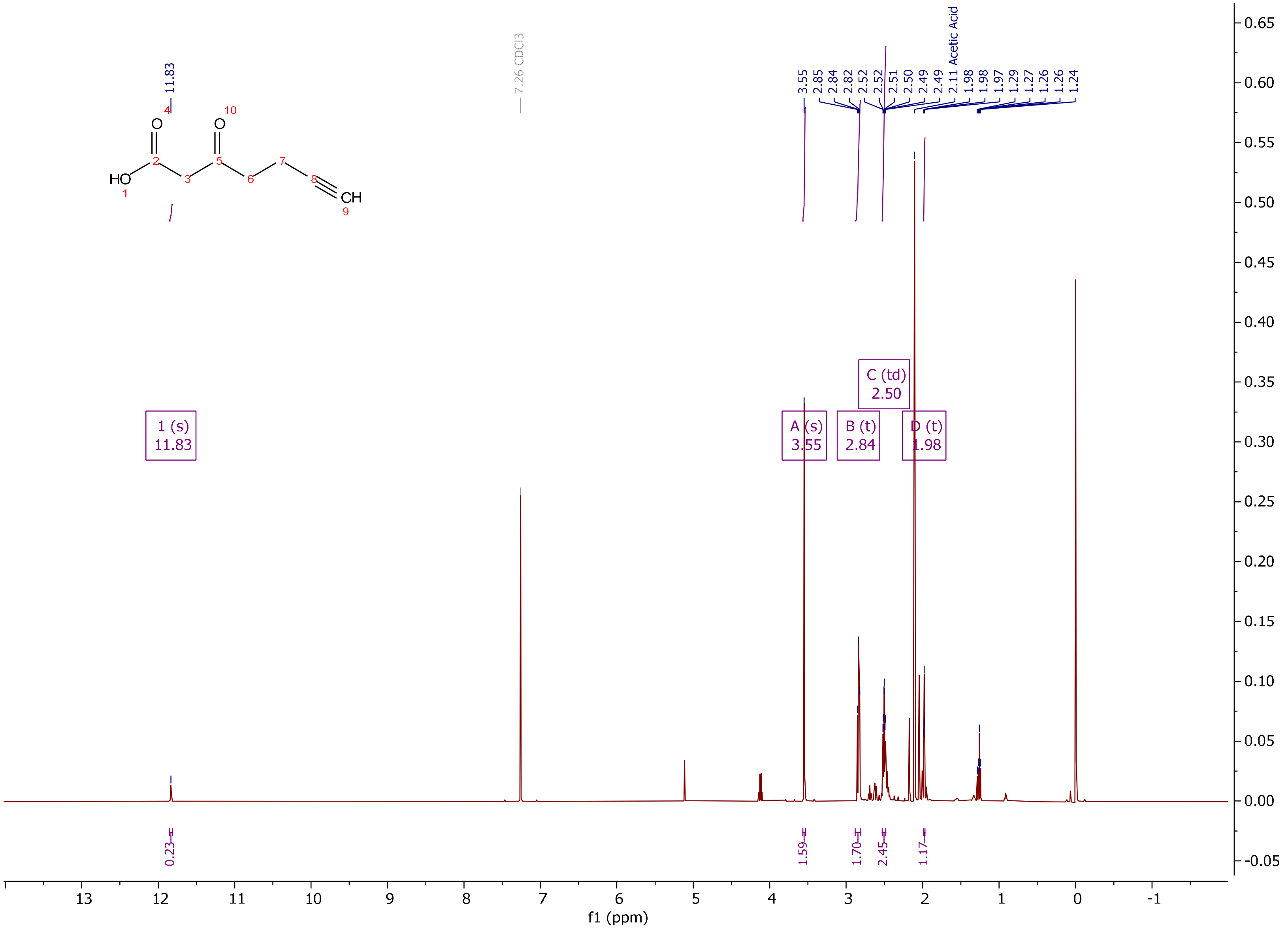

HO
O
O
CH
1 (s)
11.83
A (s)
3.55
B (t)
2.84
C (td)
2.50
D (t)
1.98
7.26 CDCl3
Acetic Acid
f1 (ppm)

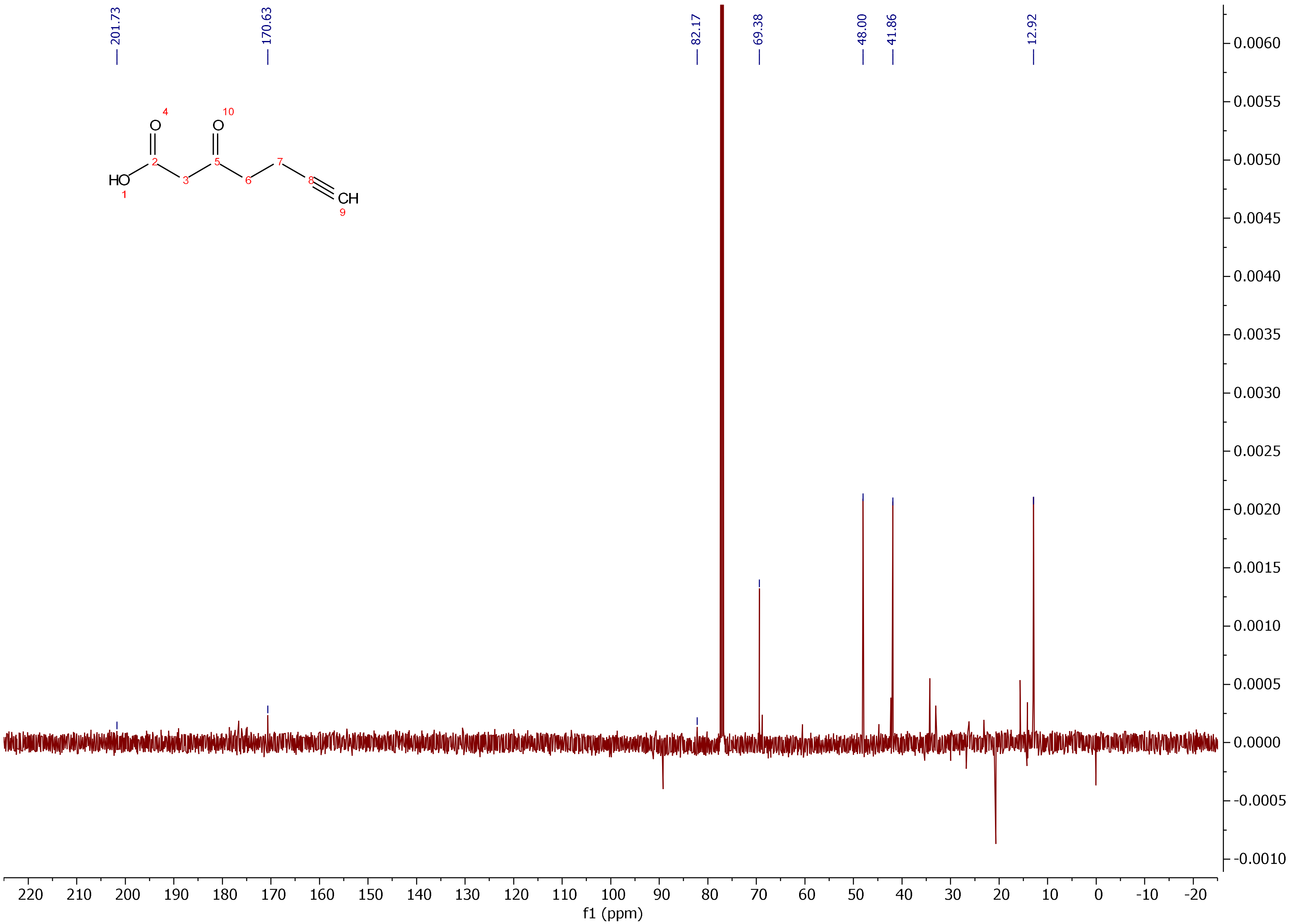
201.73
170.63
82.17
69.38
48.00
41.86
12.92
HO
CH
f1 (ppm)

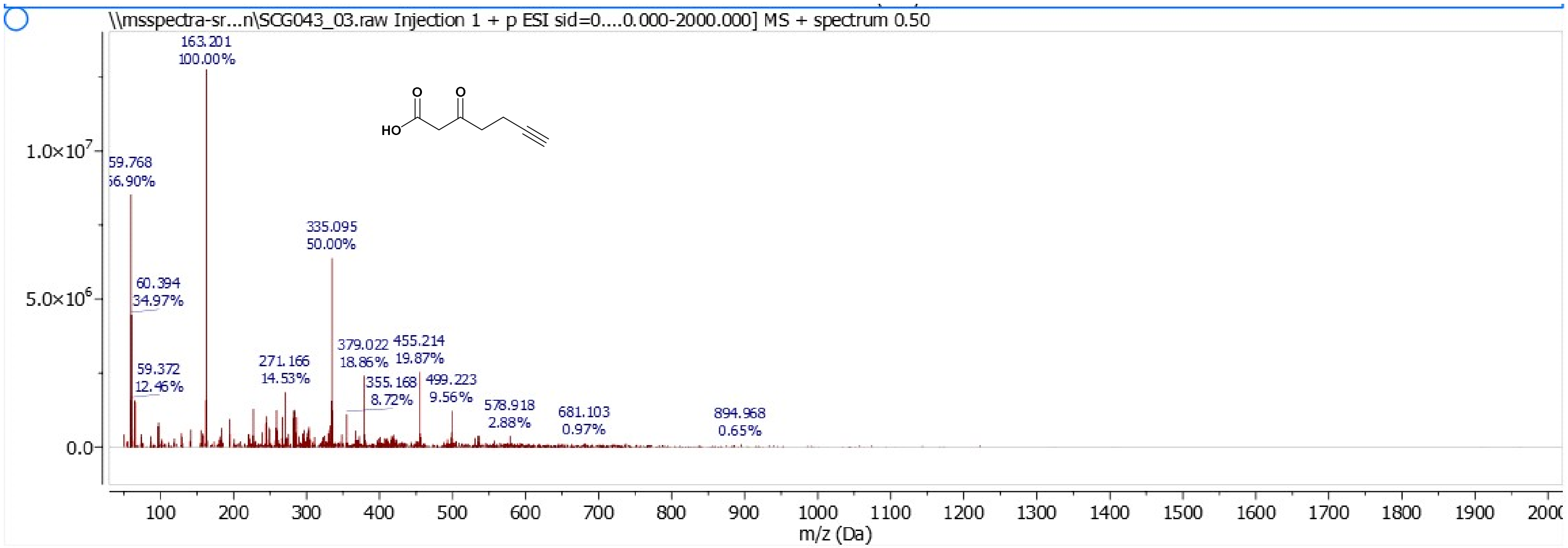
\\msspectra-sr...n\SCG043_03.raw Injection 1 + p ESI sid=0....0.000-2000.000] MS + spectrum 0.50
163.201
100.00%
HO
O
O
1.0×10^7
59.768
56.90%
335.095
50.00%
60.394
34.97%
5.0×10^6
59.372
12.46%
271.166
14.53%
379.022
18.86%
455.214
19.87%
355.168
8.72%
499.223
9.56%
578.918
2.88%
681.103
0.97%
894.968
0.65%
0.0
m/z (Da)

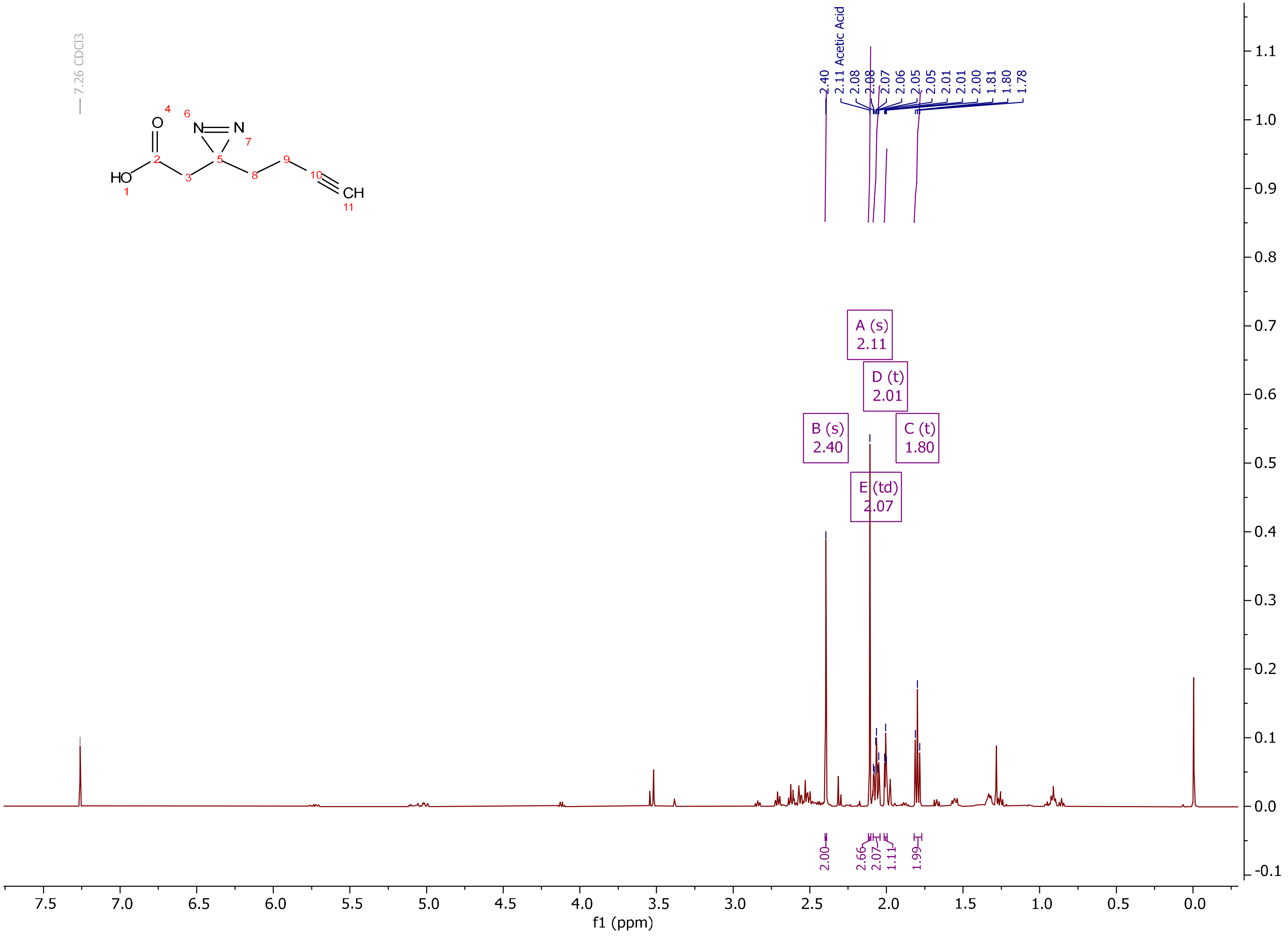
7.26 CDCl3
2.40
2.11 Acetic Acid
2.08
2.08
2.07
2.06
2.05
2.05
2.01
2.01
2.00
1.81
1.80
1.78
HO
O
N
N
CH
A (s)
2.11
D (t)
2.01
B (s)
2.40
C (t)
1.80
E (td)
2.07
2.00
2.66
2.07
1.11
1.99
f1 (ppm)

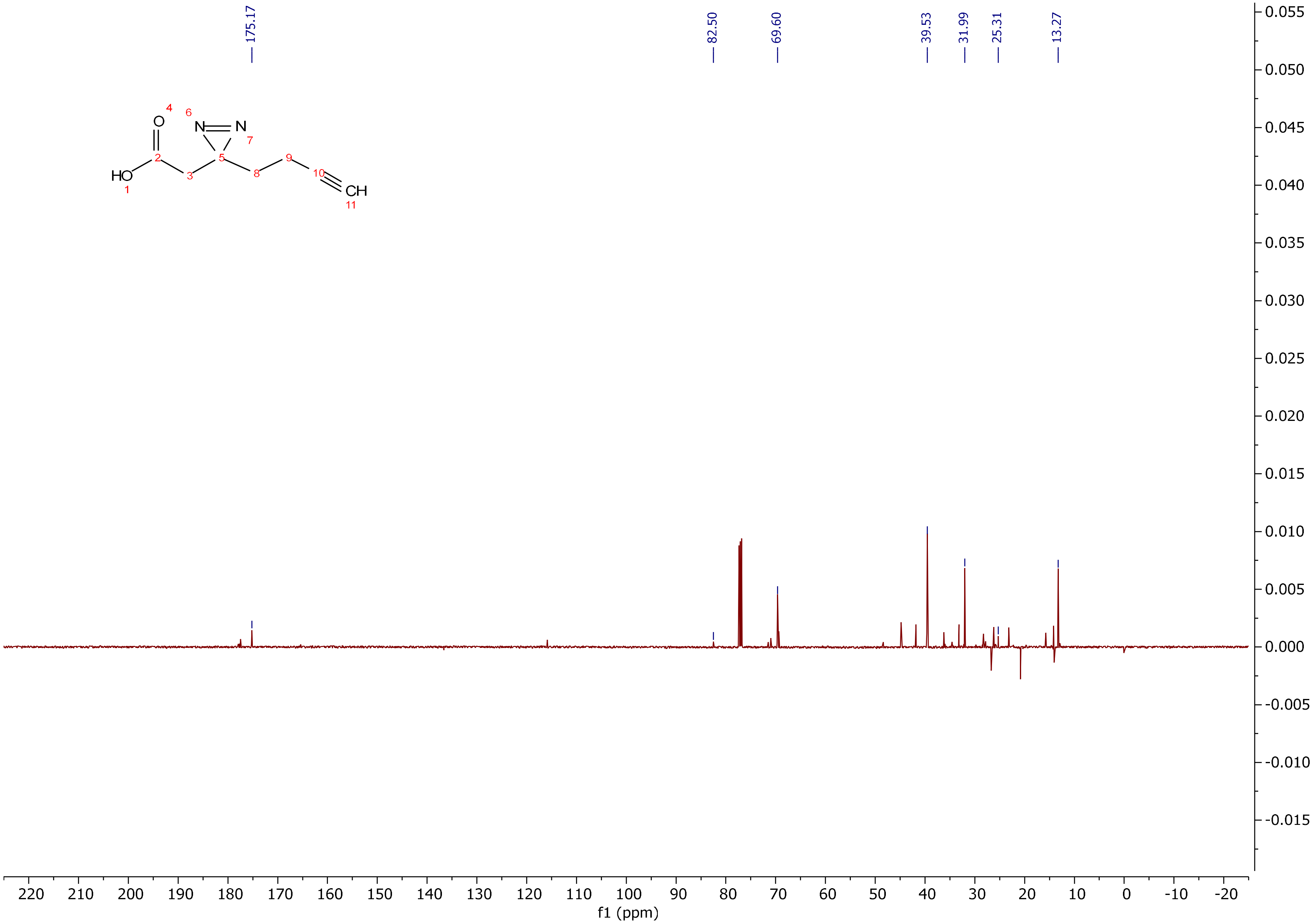
175.17
82.50
69.60
39.53
31.99
25.31
13.27
HO
O
N
N
CH
f1 (ppm)

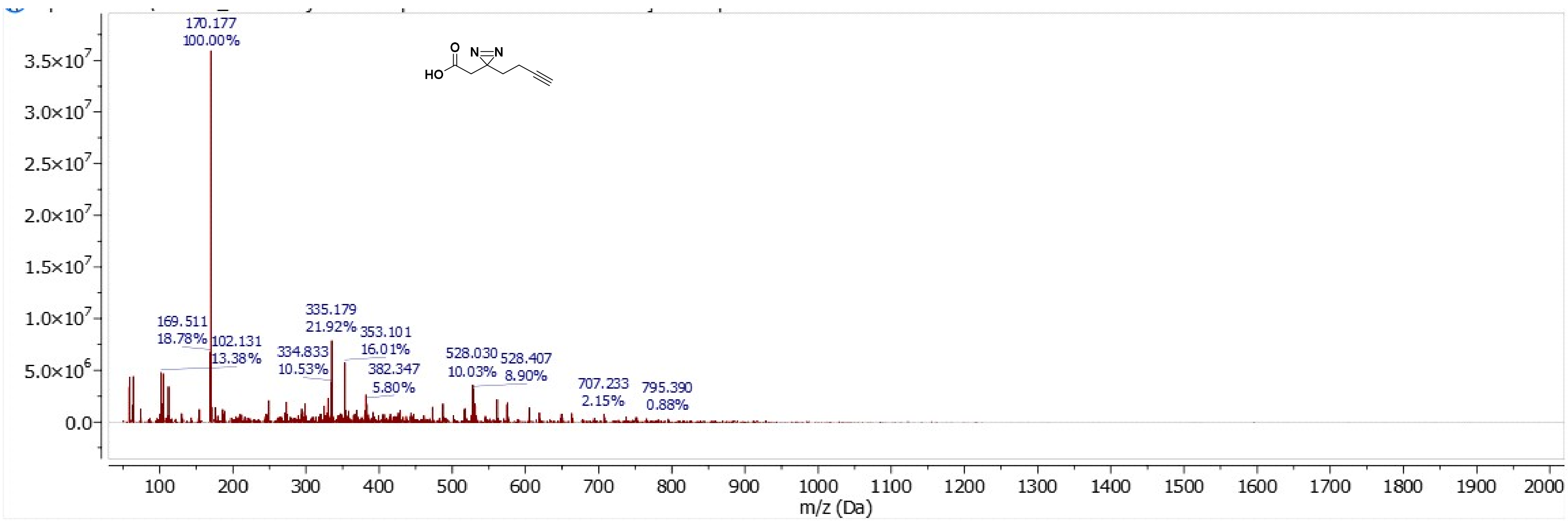
170.177
100.00%
HO
O
N=N
3.5×10^7
3.0×10^7
2.5×10^7
2.0×10^7
1.5×10^7
1.0×10^7
5.0×10^6
0.0
169.511
18.78%
102.131
13.38%
334.833
10.53%
335.179
21.92%
353.101
16.01%
382.347
5.80%
528.030
10.03%
528.407
8.90%
707.233
2.15%
795.390
0.88%
100 200 300 400 500 600 700 800 900 1000 1100 1200 1300 1400 1500 1600 1700 1800 1900 2000
m/z (Da)

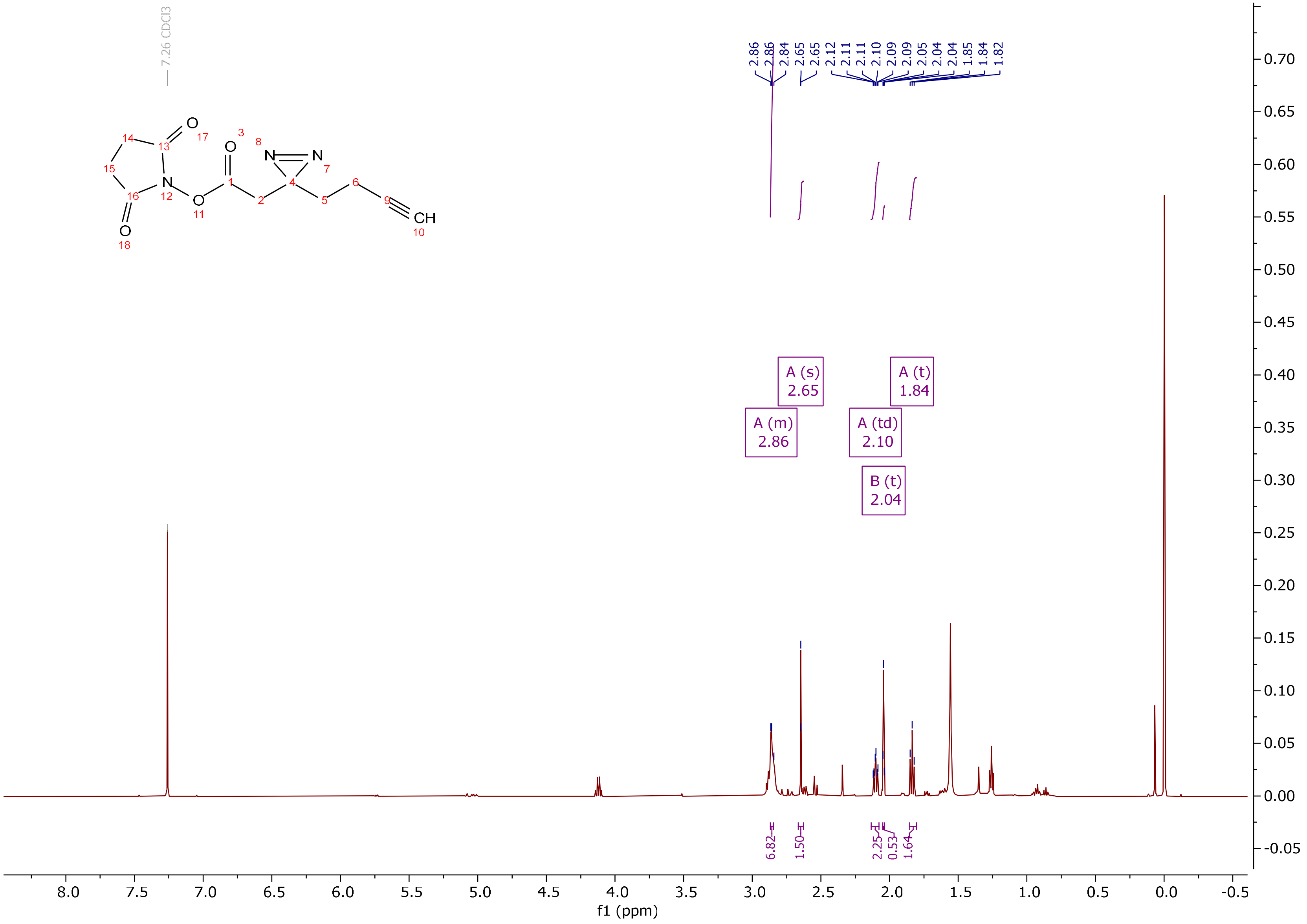

7.26 CDCl3
2.86
2.86
2.84
2.65
2.65
2.12
2.11
2.11
2.10
2.09
2.09
2.05
2.04
2.04
1.85
1.84
1.82
O
O
N
N
N
O
O
CH
O
A (s)
2.65
A (t)
1.84
A (m)
2.86
A (td)
2.10
B (t)
2.04
6.82
1.50
2.25
0.53
1.64
f1 (ppm)

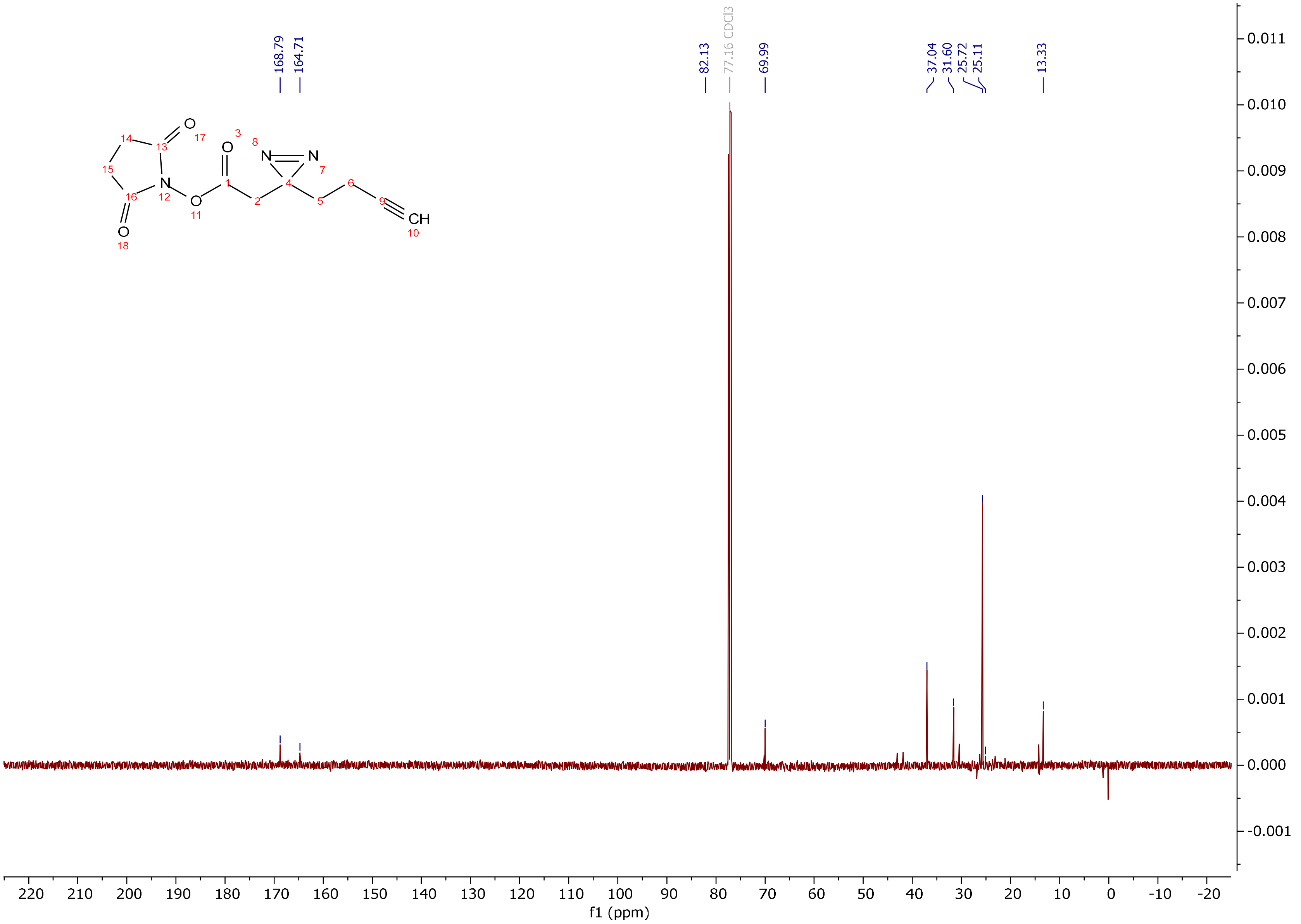
168.79
164.71
82.13
77.16 CDCl3
69.99
37.04
31.60
25.72
25.11
13.33
f1 (ppm)

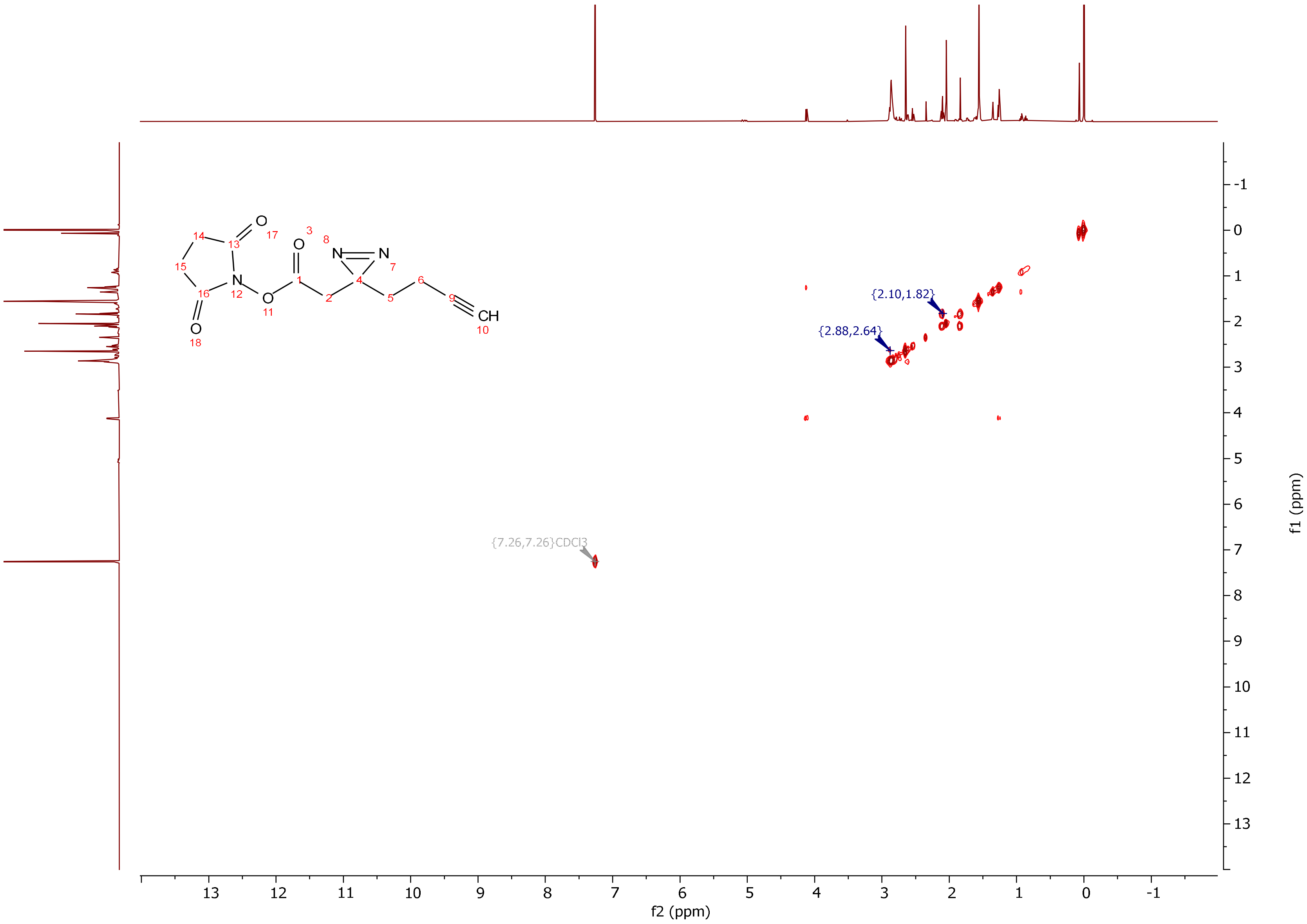

{2.10,1.82}
{2.88,2.64}
{7.26,7.26}CDCl3
f2 (ppm)
f1 (ppm)

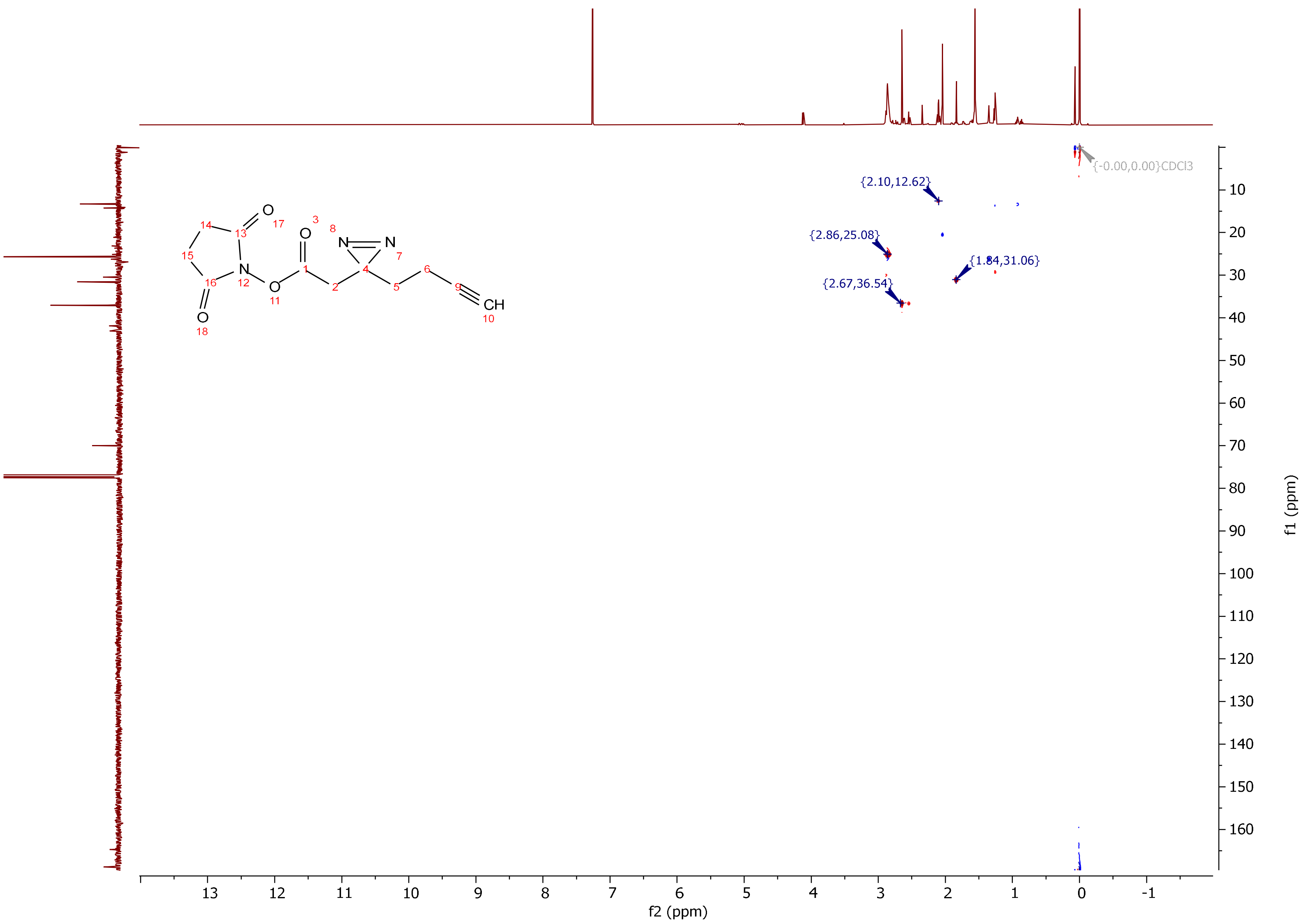
{-0.00,0.00}CDCl3
{2.10,12.62}
{2.86,25.08}
{1.84,31.06}
{2.67,36.54}
f2 (ppm)
f1 (ppm)
CH

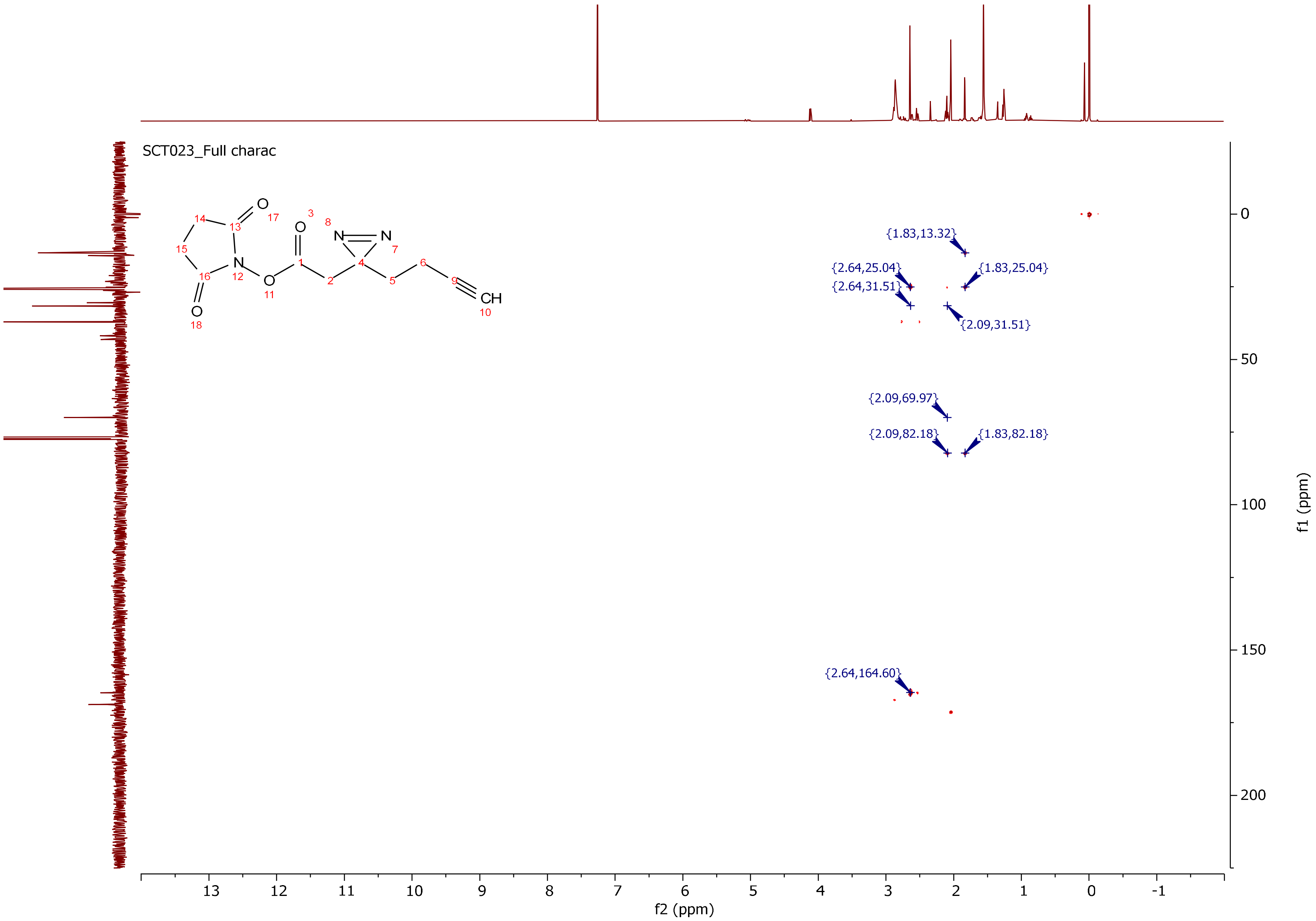
SCT023_Full charac
{1.83,13.32}
{2.64,25.04}
{1.83,25.04}
{2.64,31.51}
{2.09,31.51}
{2.09,69.97}
{2.09,82.18}
{1.83,82.18}
{2.64,164.60}
f2 (ppm)
f1 (ppm)

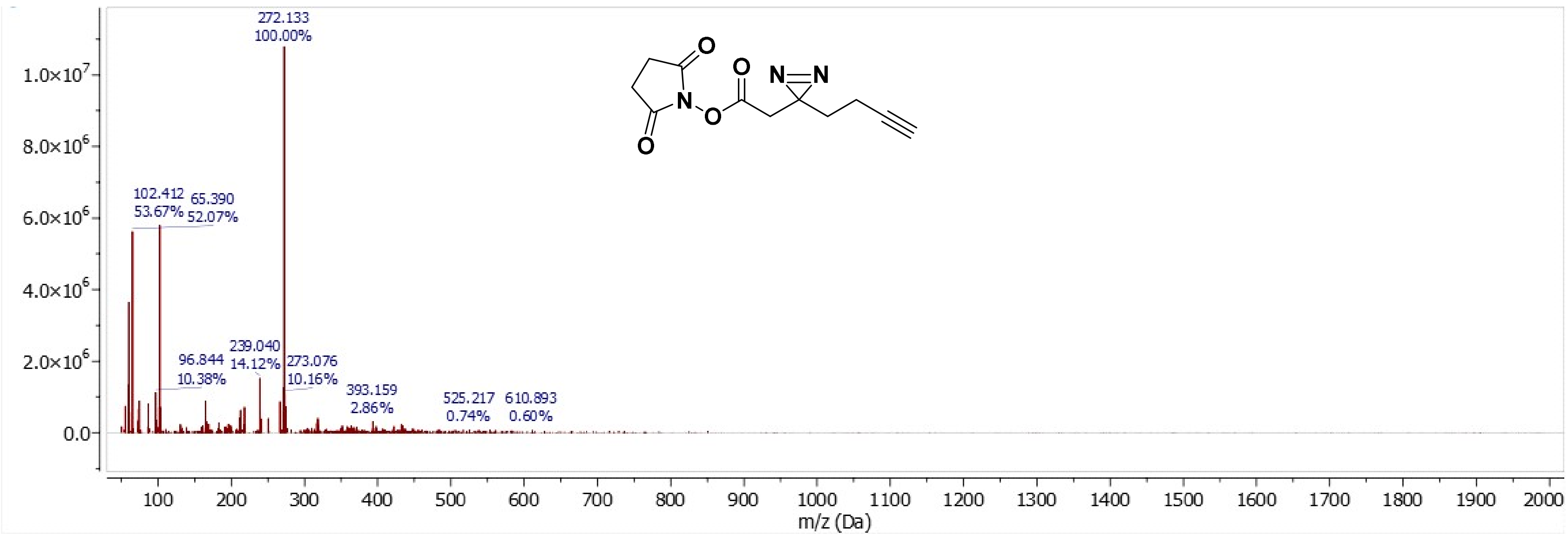

272.133
100.00%
102.412
53.67%
65.390
52.07%
96.844
10.38%
239.040
14.12%
273.076
10.16%
393.159
2.86%
525.217
0.74%
610.893
0.60%
1.0×10^7
8.0×10^6
6.0×10^6
4.0×10^6
2.0×10^6
0.0
100
200
300
400
500
600
700
800
900
1000
1100
1200
1300
1400
1500
1600
1700
1800
1900
2000
m/z (Da)

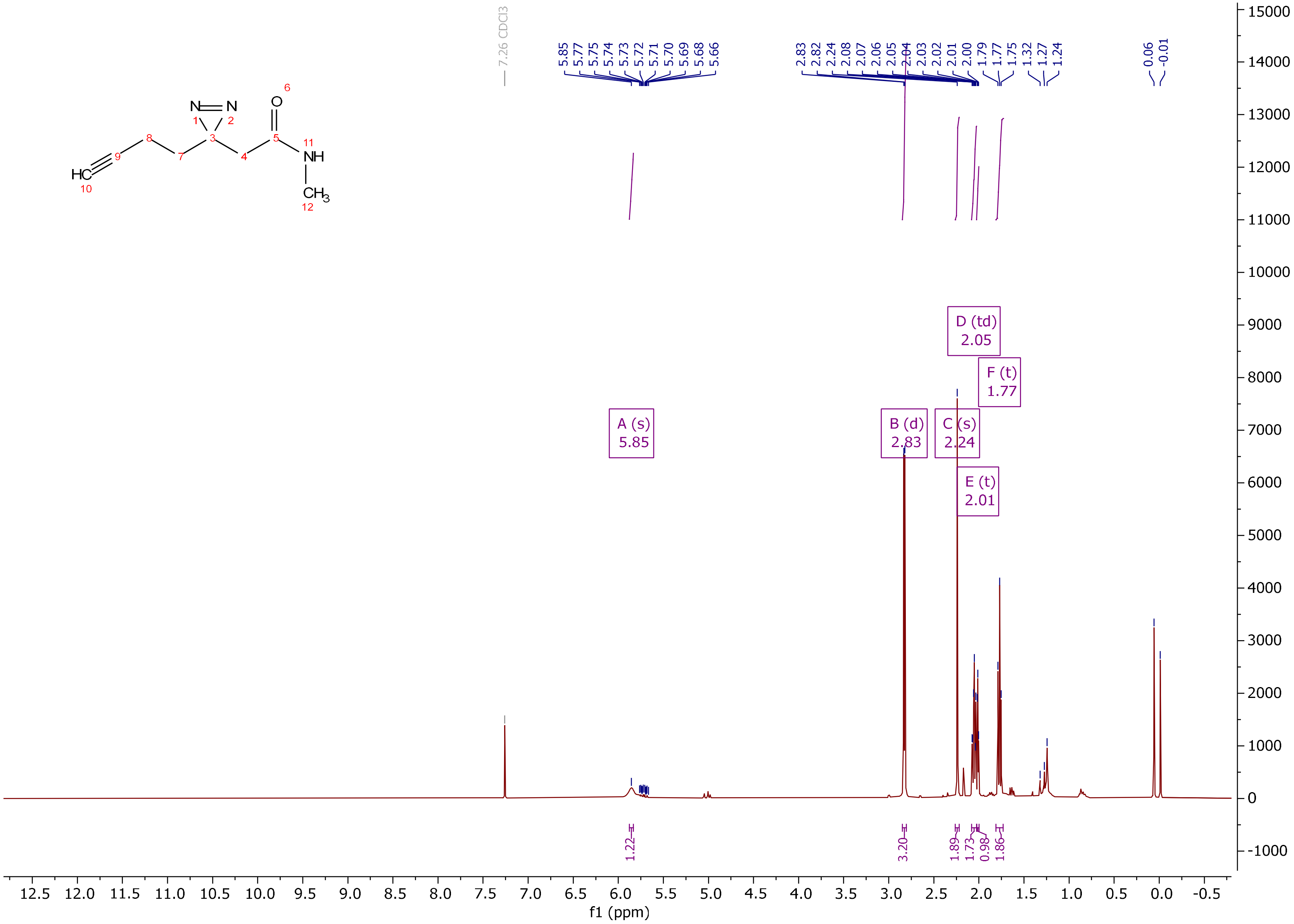

HC
NH
CH3
A (s)
5.85
B (d)
2.83
C (s)
2.24
D (td)
2.05
E (t)
2.01
F (t)
1.77
7.26 CDCl3
f1 (ppm)

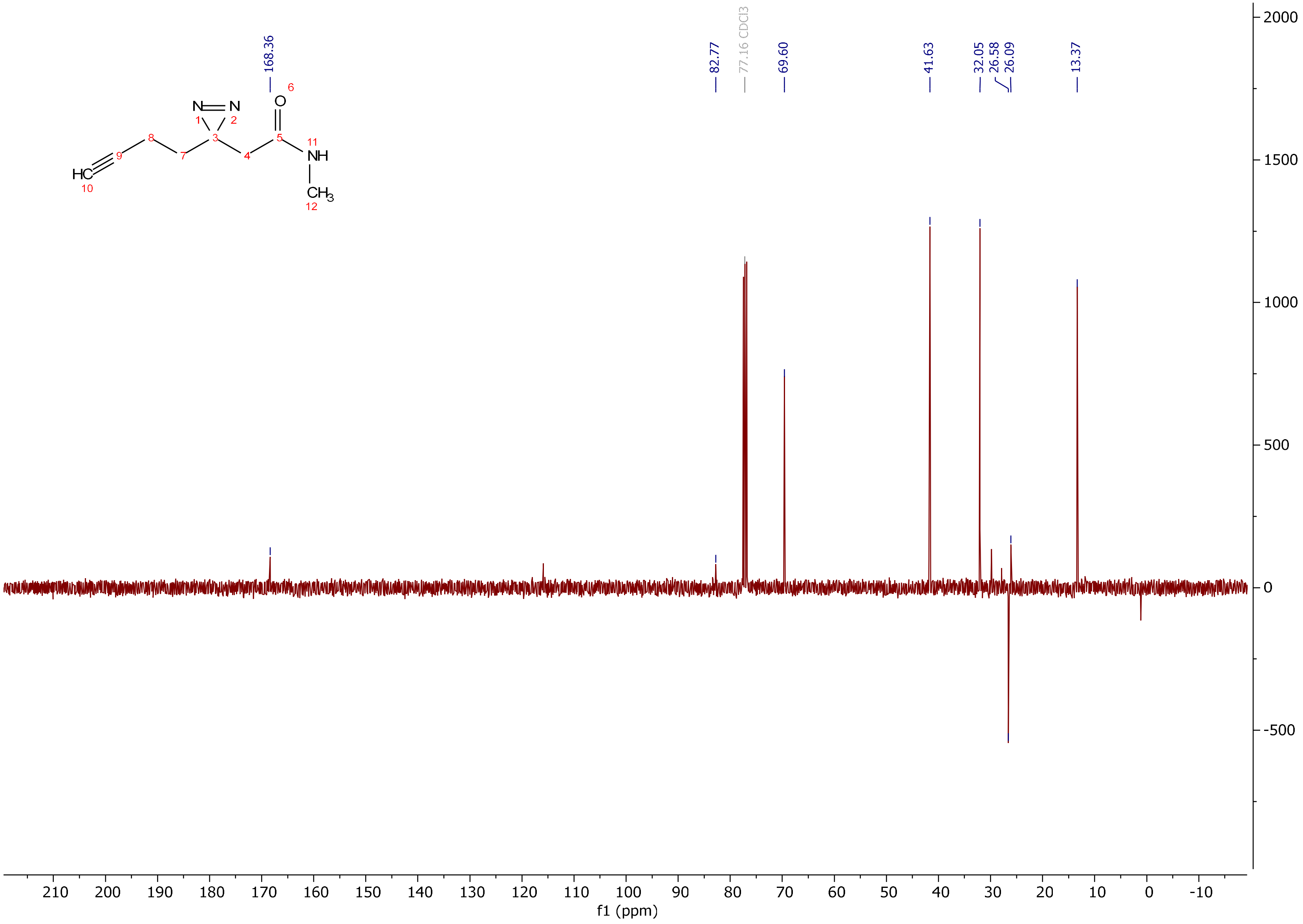
168.36
82.77
77.16 CDCl3
69.60
41.63
32.05
26.58
26.09
13.37
f1 (ppm)

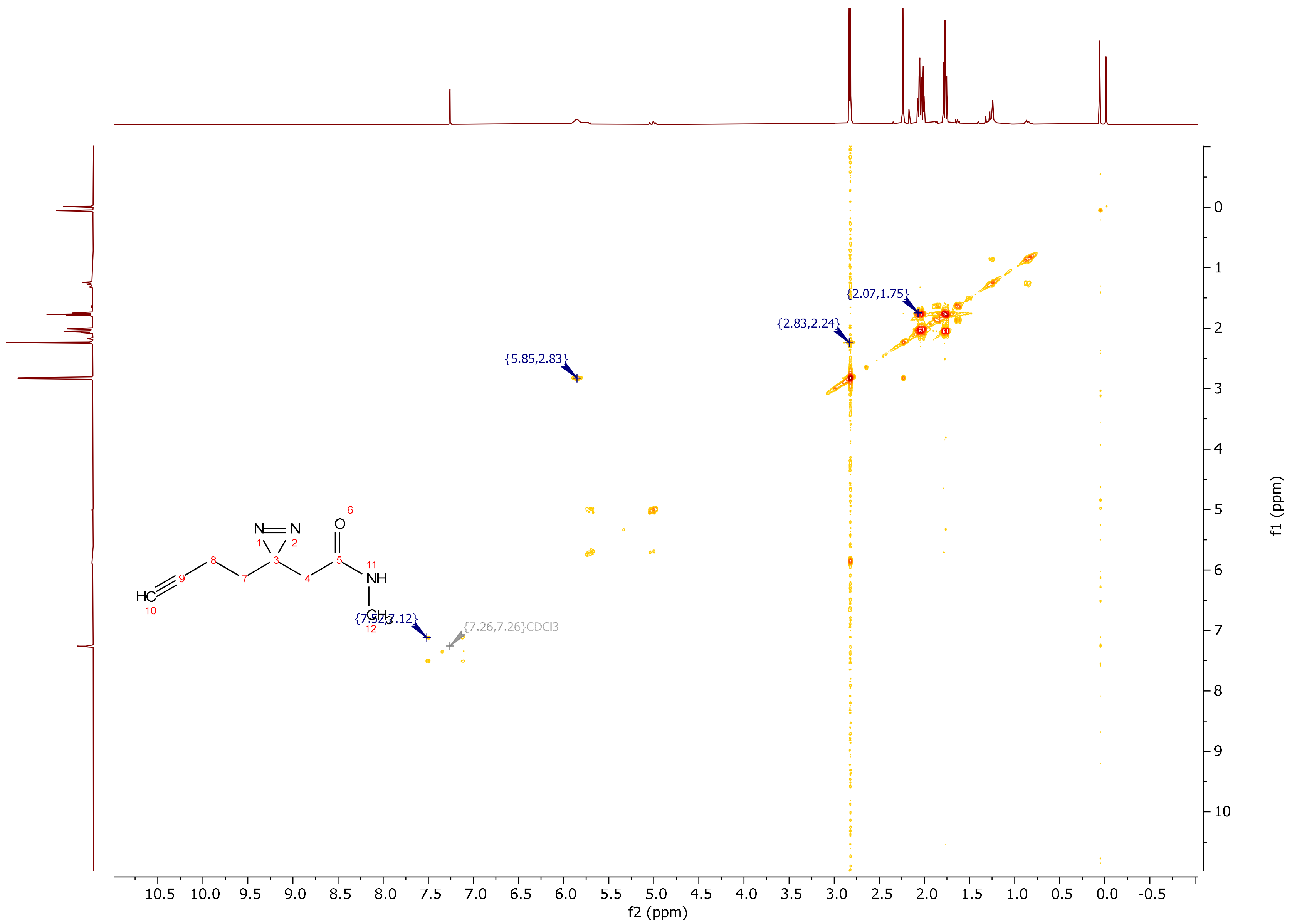
{2.07,1.75}
{2.83,2.24}
{5.85,2.83}
{7.26,7.26}CDCl3
f2 (ppm)
f1 (ppm)
NH
HC
O

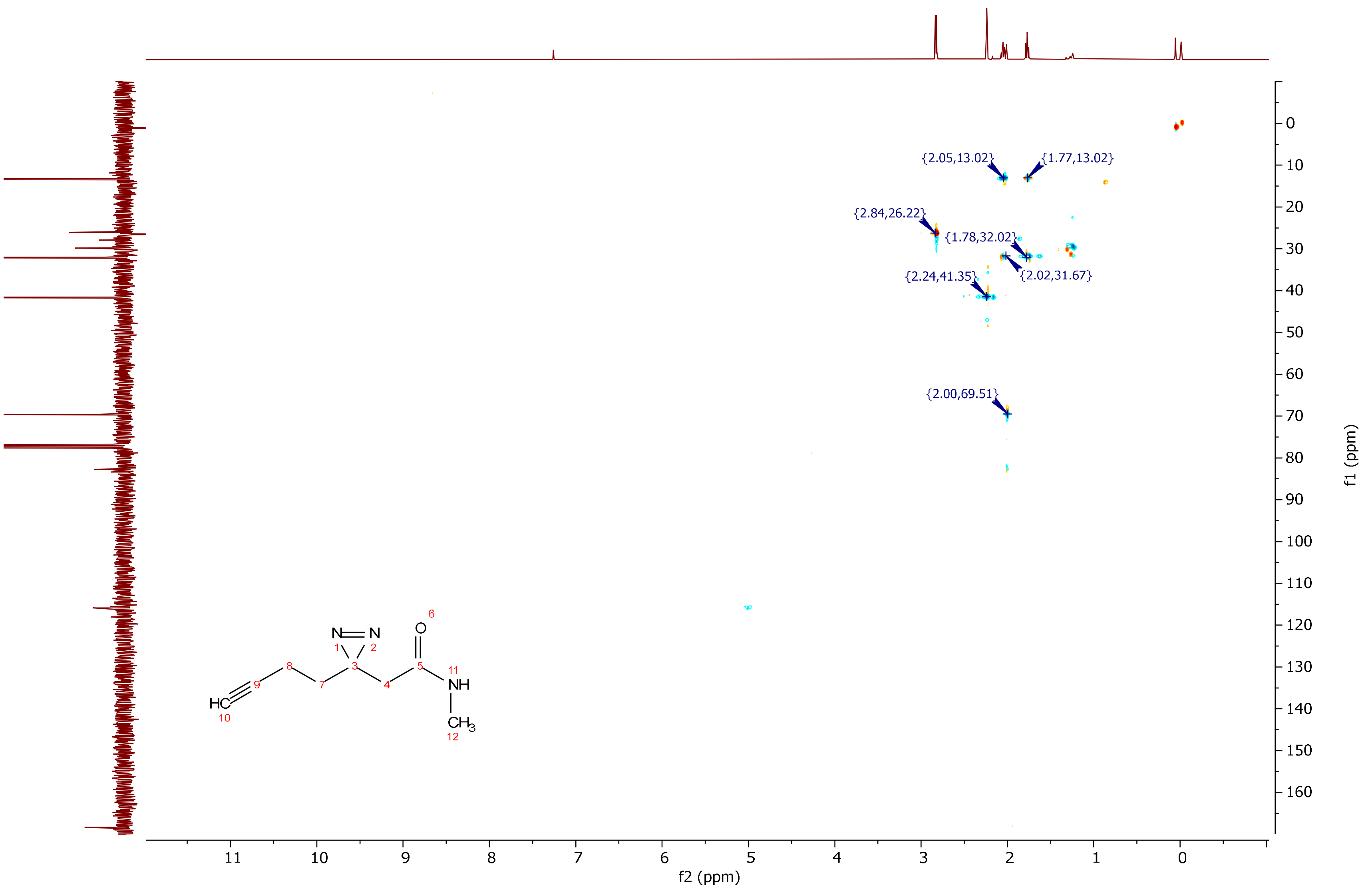
{2.05,13.02}
{1.77,13.02}
{2.84,26.22}
{1.78,32.02}
{2.02,31.67}
{2.24,41.35}
{2.00,69.51}
f2 (ppm)
f1 (ppm)

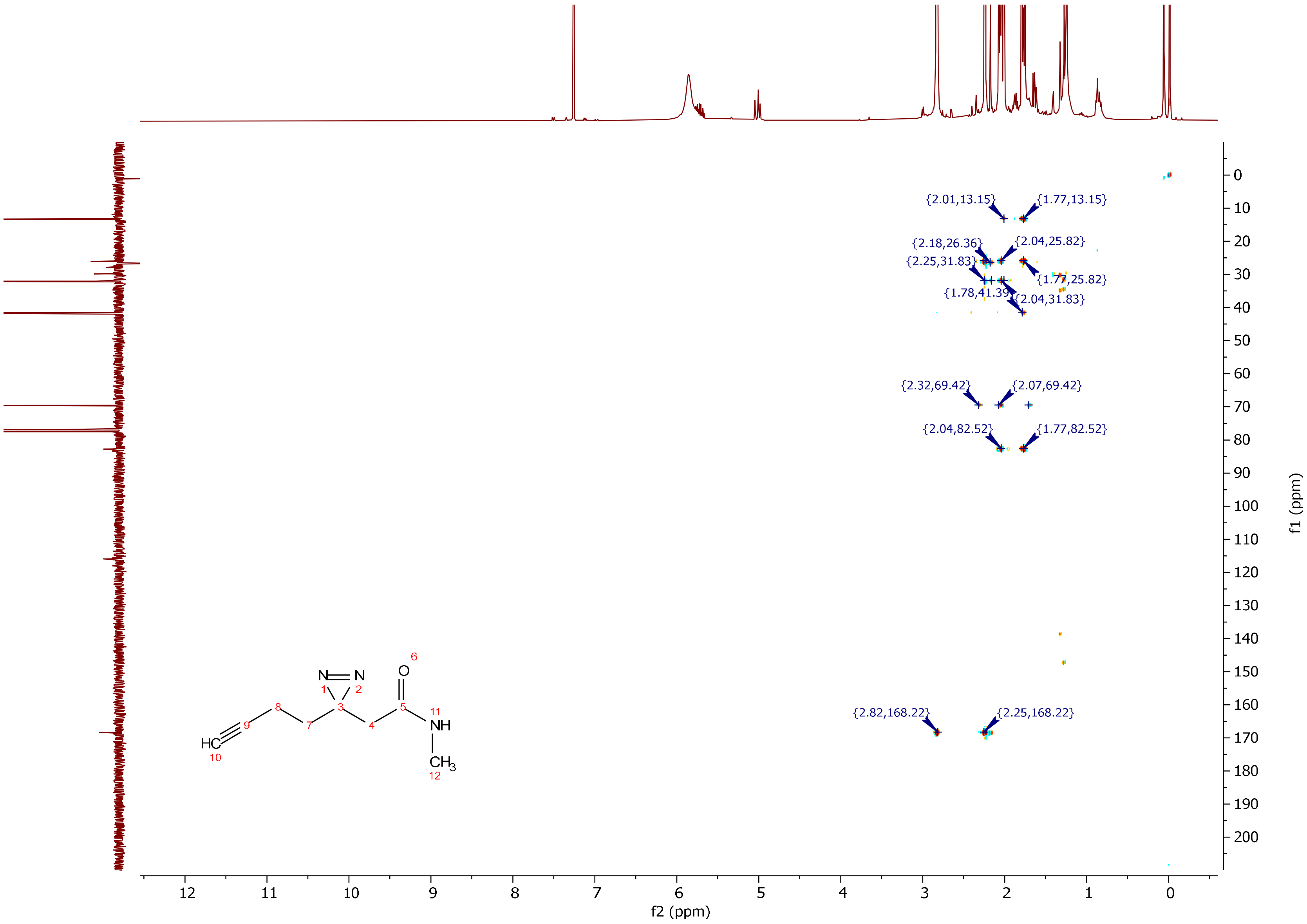
{2.01,13.15}
{1.77,13.15}
{2.18,26.36}
{2.04,25.82}
{2.25,31.83}
{1.77,25.82}
{1.78,41.39}
{2.04,31.83}
{2.32,69.42}
{2.07,69.42}
{2.04,82.52}
{1.77,82.52}
{2.82,168.22}
{2.25,168.22}
f2 (ppm)
f1 (ppm)
N N O HC NH CH3

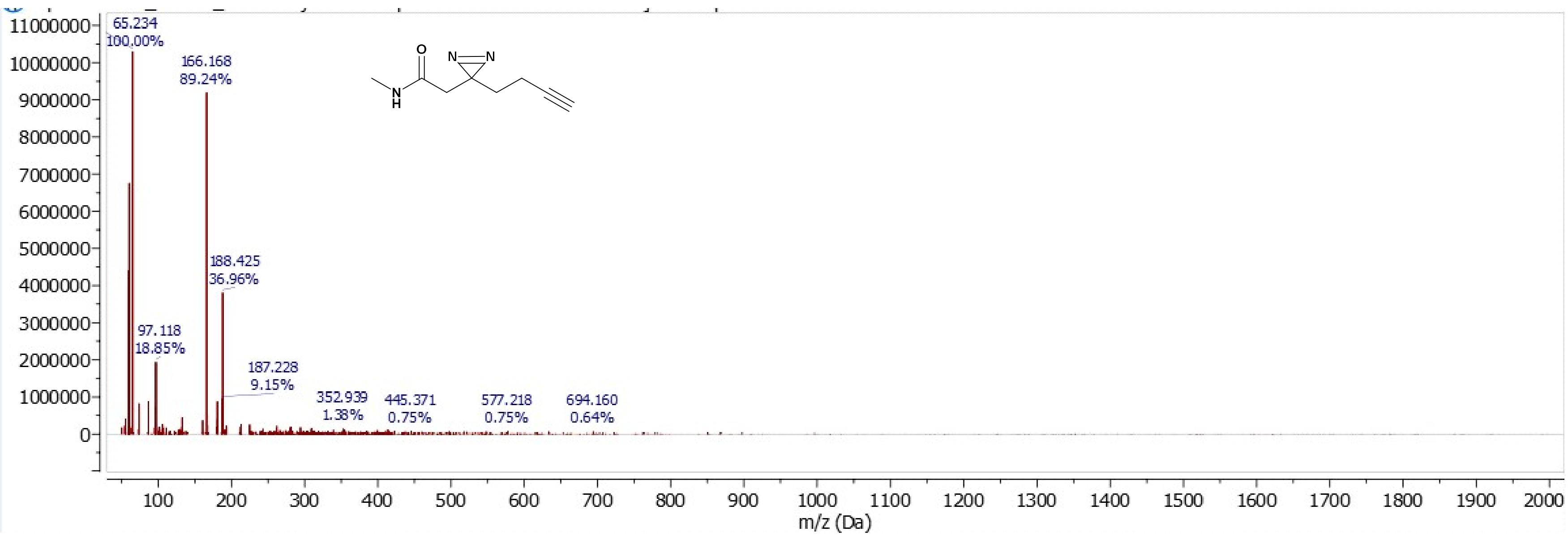
65.234
100.00%
166.168
89.24%
188.425
36.96%
97.118
18.85%
187.228
9.15%
352.939
1.38%
445.371
0.75%
577.218
0.75%
694.160
0.64%
O
N
H
N
N
m/z (Da)

REFERENCES

1 H. Salgado, S. Gama-Castro, P. Lara, C. Mejia-Almonte, G. Alarcón-Carranza, A. G. López-Almazo, F. Betancourt-Figueroa, P. Peña-Loredo, S. Alquicira-Hernández, D. Ledezma-Tejeida, L. Arizmendi-Zagal, F. Mendez-Hernandez, A. K. Diaz-Gomez, E. Ochoa-Praxedis, L. J. Muñiz-Rascado, J. S. García-Sotelo, F. A. Flores-Gallegos, L. Gómez, C. Bonavides-Martínez, V. M. del Moral-Chávez, A. J. Hernández-Alvarez, A. Santos-Zavaleta, S. Capella-Gutierrez, J. L. Gelpi and J. Collado-Vides, *Nucleic Acids Research*, 2024, **52**, D255–D264.

2 Q. Vicens, E. Mondragón and R. T. Batey, *Nucleic Acids Research*, 2011, **39**, 8586–8598.

3 H. Berman, J. Westbrook, Z. Feng, G. Gilliland, B. H. Bhat, H. Weissig, I. Shindyalov and P. E. Bourne, *Nucleic Acids Research*, 2000, **28**, 235–242.

4 M. Habibian, W. A. Velema, A. M. Kietrys, Y. Onishi and E. T. Kool, *Org. Lett.*, 2019, **21**, 5413–5416.

5 B. Langmead and S. L. Salzberg, *Nat Methods*, 2012, **9**, 357–359.

6 Y. Liao, G. K. Smyth and W. Shi, *Bioinformatics*, 2014, **30**, 923–930.

7 M. D. Robinson, D. J. McCarthy and G. K. Smyth, *Bioinformatics*, 2010, **26**, 139–140.

8 S. Crielaard, R. Maassen, T. Vosman, I. Rempkens and W. A. Velema, *Journal of the American Chemical Society*, 2022, **144**, 10462–10470.

9 K. A. Wilkinson, E. J. Merino and K. M. Weeks, *Nature Protocols*, 2006, **1**, 1610–1616.

10 T. E. Edwards and A. R. Ferré-D’Amaré, *Structure*, 2006, **14**, 1459–1468.

11 J. Schindelin, I. Arganda-Carreras, E. Frise, V. Kaynig, M. Longair, T. Pietzsch, S. Preibisch, C. Rueden, S. Saalfeld, B. Schmid, J. Y. Tinevez, D. J. White, V. Hartenstein, K. Eliceiri, P. Tomancak and A. Cardona, *Nature Methods*, 2012, **9**, 676-682.

12 Molecular Operating Environment (MOE) 2016, Chemical Computing Group Inc: 1010 sherbooke St. West, Suite #910, Montreal, QC, Canada, H3A 2R7.

13 A. Serganov, L. Huang and D. J. Patel, *Nature*, 2009, **458**, 233–237.

14 J. Yoo and A. Aksimentiev, *J. Phys. Chem. Lett.*, 2016, **7**, 3812–3818.

15 W. He, N. Naleem, D. Kleiman and S. Kirmizialtin, *J. Phys. Chem. Lett.*, 2022, **13**, 3400–3408.

16 S. Forli, R. Huey, M. E. Pique, M. F. Sanner, D. S. Goodsell and A. J. Olson, *Nat Protoc*, 2016, **11**, 905–919.

17 L. Kalinowsky, J. Weber, S. Balasupramaniam, K. Baumann and E. Proschak, *ACS Omega*, 2018, **3**, 5704–5714.

18 C. R. Corbeil, C. I. Williams and P. Labute, *J Comput Aided Mol Des*, 2012, **26**, 775–786.

19 E. C. Meng, T. D. Goddard, E. F. Pettersen, G. S. Couch, Z. J. Pearson, J. H. Morris and T. E. Ferrin, *Protein Science*, 2023, **32** (e4792), 1–13.

20 D. Pedrolli, S. Langer, B. Hobl, J. Schwarz, M. Hashimoto and M. Mack, *The FEBS Journal*, 2015, **282**, 3230–3242.

21 A. Chang Y., V. W. Chau, J. A. Landas and Y. Pang, *JEMI methods*, 2017, **1**, 22–25.

22 J. Chao, L. Yuanhai, N. Mengting, *Amino Acid Compound Containing Diaziridine Group and Synthesis Method Thereof*, **2019**, CN111087344A, 1-9.
